# Analysis of Nucleotide Variations in Human G-Quadruplex Forming Regions Associated with Disease States

**DOI:** 10.3390/genes14122125

**Published:** 2023-11-25

**Authors:** Aryan Neupane, Julia H. Chariker, Eric C. Rouchka

**Affiliations:** 1 School of Graduate and Interdisciplinary Studies, University of Louisville, Louisville, KY 40292, USA; aryan.neupane@louisville.edu; 2 Department of Neuroscience Training, University of Louisville, Louisville, KY 40292, USA; julia.chariker@louisville.edu; 3 Kentucky IDeA Network of Biomedical Research Excellence (KY INBRE) Bioinformatics Core, University of Louisville, Louisville, KY 40292, USA; 4 Department of Biochemistry and Molecular Genetics, University of Louisville, Louisville, KY 40292, USA

**Keywords:** G-quadruplex, SNV, G4, COSMIC, CLINVAR

## Abstract

While the role of G quadruplex (G4) structures has been identified in cancers and metabolic disorders, single nucleotide variations (SNVs) and their effect on G4s in disease contexts have not been extensively studied. The COSMIC and CLINVAR databases were used to detect SNVs present in G4s to identify sequence level changes and their effect on the alteration of the G4 secondary structure. A total of 37,515 G4 SNVs in the COSMIC database and 2378 in CLINVAR were identified. Of those, 7236 COSMIC (19.3%) and 457 (19%) of the CLINVAR variants result in G4 loss, while 2728 (COSMIC) and 129 (CLINVAR) SNVs gain a G4 structure. The remaining variants potentially affect the folding energy without affecting the presence of a G4. Analysis of mutational patterns in the G4 structure shows a higher selective pressure (3-fold) in the coding region on the template strand compared to the reverse strand. At the same time, an equal proportion of SNVs were observed among intronic, promoter, and enhancer regions across strands.

## 1. Introduction

**Figure 1:**
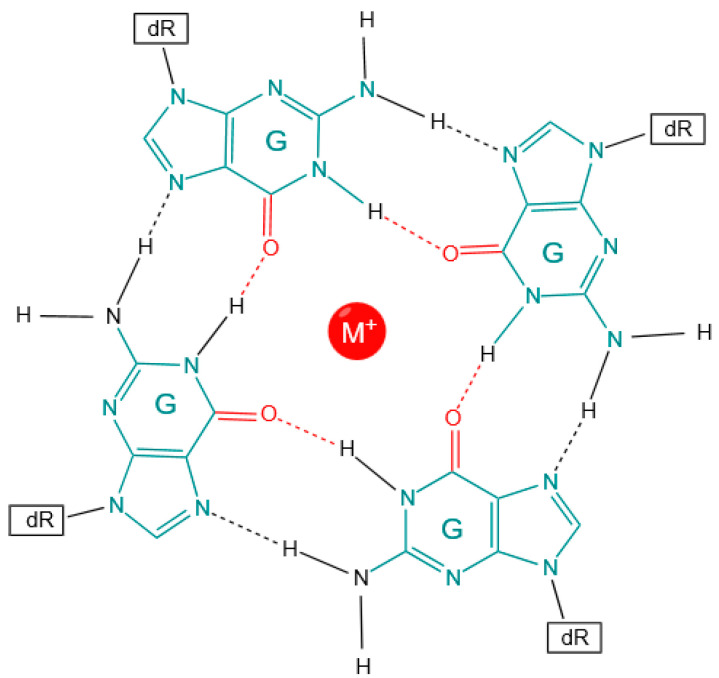
G4 structure of a guanine tetrad formed via Hoogsteen bond formation. dR: sugar–phosphate groups; cyan: guanine nucleotides; red: Hoogsteen bonds.

G-quadruplexes (G4s) are stranded secondary structures of nucleic acids rich in guanine. These nucleic acid sequences are characterized by four runs of at least three guanines separated by short loops, which can potentially fold into an intramolecular or intermolecular G4 structure [1]. The guanine tetrads are stacked on each other and held together by mixed loops of DNA, giving a four-stranded structure with nucleobases on the inside forming a Hoogsteen base pairing and the sugar-phosphate backbone on the outside (Figure 1). They are found in G-rich sequences of both DNA and RNA and are stabilized by metal cations such as potassium (K^+^) or sodium (Na^+^) [2]. The binding energy is held through the Hoogsteen hydrogen bonding between the guanines, stabilized by π–π and the charge interactions between the sixth position of oxygen (O6) and cations (K^+^, Na^+^) between the stacks. The structural architecture of a G4 is quite diverse and can form different topologies based on factors, including the chemical environment, loop length [3,4], and localization in the sequence or structure molecularity [5]. The stacking of the guanine tetrads is bound by the loops of nucleotide bases of variable sizes, which determine the folding of the secondary structure. 

### 1.1. Functional Role of G4 Regions

Guanine-rich sequences do not always form G4 structures, which can be dependent upon physiological conditions and methylation patterns guided by a chromatin structure for their formation [6]. However, when they do, they affect molecular function. One such perturbation is transcription, which is affected by stalling the replication fork [7,8,9]. In cells that do not have the normal DNA repair machinery, this causes the downregulation of several genes and cell cycle arrest [10].

Additionally, G4 structures, G4 stabilizing agents, and double-stranded breaks (DSBs) facilitate the homologous recombination repair pathway affecting genome instability. Based on the size of the G4, thermodynamically stable short-loop structures within the G4 have been extensively studied to cause instability in replication-dependent processes [11]. The alteration of the DNA polymerase function and helicases in sites of G4 formation has been well established and is used to identify G4s in vivo [12,13].

While some ligands have shown a binding affinity towards G4 structures for the treatment of cancer-specific cells and transcriptional alteration [14], the binding of other ligands that stabilize G4 leads to multiple DNA damage [14], micronuclei formation, delayed replication fork progression [15], and telomeric defects [16,17,18].

### 1.2. Mutations within G4 Regions

DNA lesions can be mutagenic or lethal, and when they are found in G4 regions, they can alter the secondary structure by changing the guanine tract base pairing or altering the composition of the loop region. A single nucleotide polymorphism (SNP) in the G4 present in the promoter region of c-MYC has been shown to change the transcription in vivo [19]. Mass spectroscopy studies using single-nucleotide substitution in the central block of parallel G4 forming sequences found a deleterious effect on G4 stability and association rate [20]. A trinucleotide CGG repeat expansion in the untranslated region of the *FMR1* gene has been linked with ataxias and fragile X syndrome [21]. A T→C SNP at the GC-rich apolipoprotein E (APOE) region is known to vary G4 structure and has been linked to the onset of Alzheimer’s disease [22].

It has been proposed that specific helicases promote genomic stability by actively resolving G4 structures, which can be altered by adding G4 stabilization ligands in the presence of specific DSBs [10,12,23]. Baral et al. identified several eQTL variants in potential G4 regions [24]. Changes in G4 loops led to a significant alteration in gene expression among individuals, further fueling the structural role of G4s in regulating and binding transcription factors [23].

A selective mutation of the G-rich region to disrupt the G4 structure has been found to alter transcription [25]. Mutations can hinder the recruitment of transcription factors that overlap the G-rich region and function as recognition motifs or bind to the G4 structure. Siddiqui-Jain et al. demonstrated that a single G→A mutation destabilizes the folding of G4 in the Pu27 region of MYC, which is otherwise repressed, resulting in a threefold increase in transcriptional activity of the gene in tumor cell lines [19]. Studies related to 8-oxoguanine (8-oxoG) in G4 established the presence of G-A and guanine abasic lesions, which can destabilize the secondary structure, leaving the unfolded sequence prone to cleavage [26].

### 1.3. Study Motivation

To date, the majority of G4 studies have focused on the identification of G4s [27,28], their association with different features of interest [6,16,29,30,31], and the determination of which putative genomic regions form structural G4s [32,33]. Few studies have looked at how single nucleotide polymorphisms affect G4 formation, although SNVs within G-quadruplexes have been shown to be less frequent than random, suggesting they are under selective pressure [34]. In most cases, these are focused on regions of interest, such as telomeres [35] or specific genes [36,37]. Previous efforts have determined that G4s, in general, are enriched around breakpoints associated with structural variants in cancer [38,39]. The most comprehensive study to date associated SNVs in general with G4 variations, resulting in the identification of more than 5 million gains or losses of G4s genome wide, with the majority occurring within genic regions and a specific enrichment in oncogenes [25].

Given the roles that G4 regions and mutations within them play in transcriptional and translational control and the lack of information concerning how SNVs in G4 regions affect specific disease states, we set out to identify the impacts of mutations in G4 regions and patterns associated with the variants in germline and somatic cells. We compared annotated G4 regions with overlapping variants annotated in the COSMIC [40] and CLINVAR [41] databases. These represent somatic mutations associated with cancers (COSMIC) or germline mutations with clinical relevance (CLINVAR). Because of their high stability and increased cellular uptake, G4 sequences have interesting diagnostic and therapeutic functions. Understanding how known variants in the genome confer stability or disrupt the G4 sequences will allow a better understanding of G4 structure and function.

## 2. Materials and Methods

### 2.1. Putative and Validated G4 Identification

Quadparser version 2 [28] with the default parameters was used to identify 175,778 putative G4 regions (pG4) in the human genome hg38.p13 assembly across both strands. Experimentally validated G4 regions were obtained from an experiment utilizing a method called G4 Seq (GEO accession GSE63874) previously performed by Chambers et al. [33]. The intersection between the putative and experimental G4s was found using BEDTOOLS v2.27.1 [42].

### 2.2. Somatic and Germline Variants in G4 Regions

Cancer-specific curated somatic mutations from the COSMIC database v96 [40] were used for the analysis. COSMIC contains 22,996,215 distinct single nucleotide variants (SNVs) (19,721,019 non-coding variants (NCV) and 5,977,977 coding) from 1.4 million tumor samples. An additional 550,239 germline SNVs from other clinically relevant diseases and disorders were obtained from CLINVAR [41] version 20200203.

For both sets of data, a two-pass analysis was performed. In the first pass, overlaps between the SNVs and putative G4 regions were found to determine the potential loss of a G4 structure due to mutations. In the second pass, mutations leading to a G in regions with flanking guanines that result in the gain of a G4 were detected. In each case, a variant call format (VCF) file describing the coding and non-coding mutations was obtained from COSMIC [40] and CLINVAR [41]. Using the VCF, SNVs were filtered using bcftools v1.8 [43], with insertion and deletion events (INDELs) removed (bcftools -I NCV.vcf) even though INDELS may introduce or remove G4 structures. Initially, we chose to focus on non-INDEL events since INDELS can have a higher false positive rate, particularly in homopolymeric regions [44,45].

### 2.3. Identification of SNPs Affecting G4 Formation

A window of 30 bases upstream and 30 bases downstream of each variant was used to search for putative G4 sequences. Prospective G4 regions were compared with the Vienna Package RNAfold v2.4.8 to predict changes in stability as a result of the variant [46]. The ∆MFE (minimum free energy) and the ∆ED (ensemble diversity) values were used as the determining metrics. The ∆MFE calculates the stability of the sequence structure based on the binding propensities, while the ∆ED provides the diversity of the sequence structure and alternate structures that can form. G4hunter v20150928 was used to compare the G4 scores and the formation of pG4 [27].

Based on the location of a specific SNV inside a G4 region, the relative location of the mutation was calculated as the position of the SNV in the G4 divided by the total length of the sequence. In terms of multiple potential G4 regions, the whole region was used as a single sequence, and the relative location of the mutation was calculated. Each SNV was converted into a 3-mer based on its location, and changes in the 3-mer resulting in a broken GGG quadruplex structure were calculated. For each 3-mer, the number of changes was calculated using one base before and after the variant’s location, respectively. In addition, SNVs occurring within loops were analyzed. The R package annotatr v1.1.6 was used for randomized background counts for each annotation [47].

### 2.4. Functional and Transcription Factor Enrichment Analysis

Based on the G4 identified, the hg38.p13 coordinates of the G4 were used to find the enrichment of transcriptional factors using the R package RemapEnrich v0.99.0 [48] for Hep-G2 (hepatocellular carcinoma), K562 (myelogenous leukemia), HEK293 (embryonic kidney), and HEK293T (T antigen–transformed embryonic kidney) cell lines. These cell lines were chosen as a comparative analysis of tumor vs. normal cells that could better elicit differences in the somatic mutations. All enrichment tests were calculated using hypergeometric testing. Further, an enrichment analysis of the genes with individual mutations was selected based on the number of SNVs per gene, the effect of SNVs on the G4, G4 per gene, and samples as specified in the result. The functional annotation enrichment of genes was carried out using g:Profiler functional annotation v0.2.1 [49], while the enrichment analysis of the transcription factors (TFs) involved was carried out using the STRINGdb database v11 [50]. In order to analyze the disruption of motifs by each SNV, the R package motifbreakR v2.10.0 [51] was used.

## 3. Results

### 3.1. COSMIC Somatic Mutations

**Figure 2:**
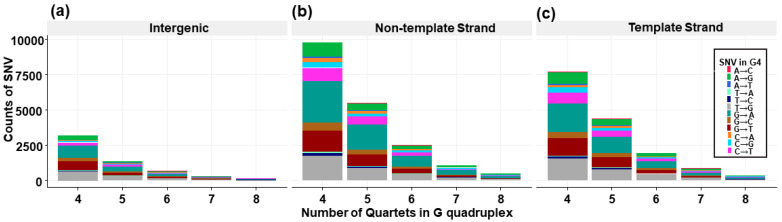
SNV mutation types found in quartets of different lengths for (**a**) intergenic regions, (**b**) non-template strand genic regions, and (**c**) template strand genic regions from the COSMIC database.

Using the COSMIC database, 37,515 (0.16% of all COSMIC mutations) distinct single nucleotide somatic mutations overlapping experimentally validated G4s were identified within 26,504 pG4 regions from 9693 genes, 8998 of which were determined to be protein coding according to ENSEMBL hg38 annotations. The remaining genes were identified as lncRNA (n = 557), miRNA (n = 111), or other (snoRNA, snRNA, pseudogenes, etc., n = 27). The most frequently observed mutation in the COSMIC-filtered dataset was the transition event G→A (28%), followed by the transversion event T→G (18%) (Figure 2a–c). We expected to see a high number of G→A and G→T (15%) mutations. However, we additionally observed T→G transversion events occurring at higher rates than A→G transitions. Comparatively, higher G/C→A/T variants in intragenic CpG islands have been observed due to the spontaneous deamination of the cytosine hypermethylated CpGs within these regions [52,53]. However, the effect of these mutations is less studied across G4 regions.

We performed a Kruskal–Wallis rank sum test based on the ∆MFE for the G4 stabilizing and destabilizing variants based on the annotation of the G4 present in 3′ UTR, 5′ UTR, CDS, and promoter regions. The results show a significant difference among the groups (H = 13,498, df = 3, *p* < 0.001). A pairwise Dunn’s test with a Benjamini–Hochberg FDR correction showed that SNVs destabilizing the G4 in CDS regions have a lower ∆MFE compared to the 5′ UTR (FDR < 0.001) and promoters (FDR < 1 × 10^−25^) across both strands. We found a lower transition:transversion ratio (Χ^2^ test, *p* = 0.00001) occurring in the G4 region (1.02), compared to all mutations in the COSMIC database (1.146) (Supplemental Tables S1 and S2).

**Figure 3:**
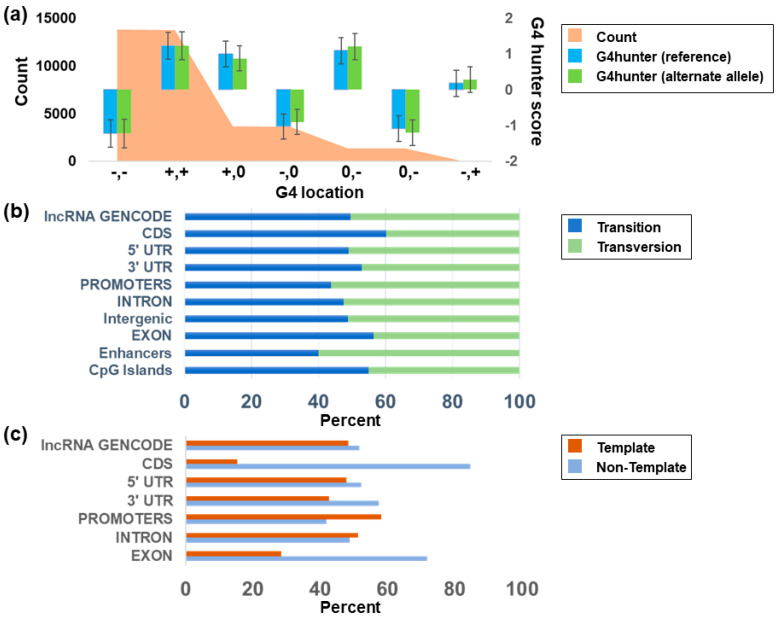
G4 variants detected within annotated functional regions (CDS, exons, 5′UTRs, 3′UTRs, CpG islands, lncRNA, introns, intergenic promoters, and enhancers). Shown are (**a**) the count of change in pG4 with a G4Hunter score across both strands before and after mutation (0: the absence of pG4; +: the presence of G4 in the forward strand; -: the presence of G4 in the reverse strand); (**b**) the percentage of the type of mutation across annotations from the COSMIC database; and (**c**) the percentage of SNVs that occur in a G4 region across the template and non-template strand for functional annotation groups within the CLINVAR database.

**Figure 4:**
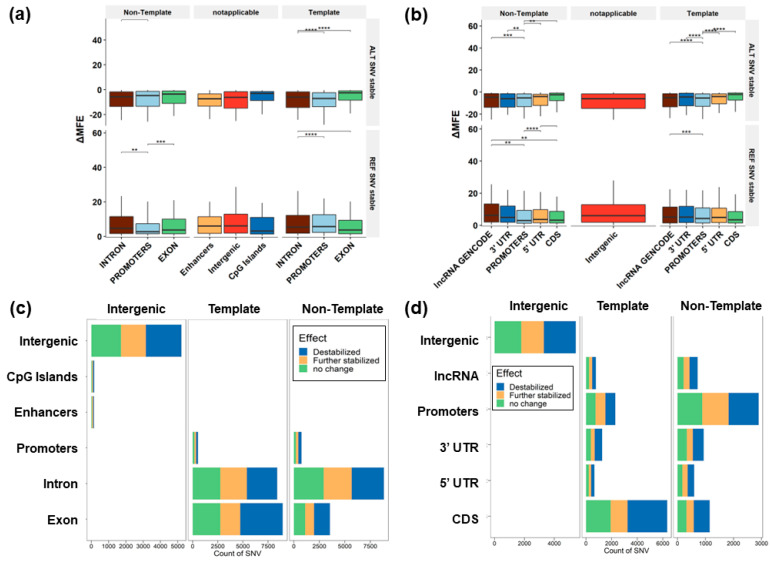
Thermodynamic changes associated with variants in various genomic features. (**a**) Non-zero DNA-based ∆MFE associated with variants in genomic features (introns, promoters, exons, enhancers, intergenic regions, and CpG islands) for the alternate allele (top) and the reference allele (bottom). (**b**) RNA-based ∆MFE associated with variants in genomic features (lncRNA, 3′UTR, promoters, 5′UTR, and intergenic regions) for the alternate allele (top) and the reference allele (bottom). (**c**) Stabilization effects for DNA-based annotations in non-gene regions (i.e., intergenic) or on the template or non-template strand for variants within gene regions. (**d**) Stabilization effects of gene-based annotations in intergenic, template, and non-template regions. **: Significant difference (*p* < 0.01 using Dunn’s test). ***: *p* < 0.001; ****: *p* < 0.0001.

Based on the G4Hunter [27] and RNAfold [46] results, we compared the number of SNV events that break the G4 structure and change the thermodynamic stability based on the MFE of each sequence. We found that 7236 (19.2% of variants in G4) of the SNVs within the G4Hunter-identified G4s resulted in the loss of a G4, while 2728 SNVs led to the gain (Figure 3a,b and Figure 4, Supplemental Table S3).

### 3.2. CLINVAR Germline Mutations

Using the CLINVAR database, 4999 SNVs were identified in pG4 regions, out of which 2378 intersect with experimental G4. Most of these G4 mutations occur in exons (65%, n = 1552). The remaining variants are found in introns (34%, n = 804) and promoters (0.8%, n = 20). In total, 97% (n = 2306) of the detected SNVs occur in protein-coding genes (Figure 3c, Supplemental Table S4).

### 3.3. Predicted Change to G4 Stability

RNAfold was used to differentiate the impact of the variant on the stacking. Variants were classified based on the change in stability and formation of available guanines for stacking by combining the sequence pattern analysis of G4Hunter with thermodynamic parameters from RNAfold (Figure 4a–d). The majority of the SNVs identified in COSMIC and CLINVAR (81%) did not affect the GGG stacking, so the formation of tetrads of guanine was impossible. Though complete structural breakage does not occur, 40% of the variants were predicted to have decreased stability in the G4 structure. This is due to the presence of additional guanines in the loop that aid the conformational diversity of the G4, which can act as an extra base for stacking. Using the combined COSMIC and CLINVAR mutations, 10,435 SNVs were predicted to increase the stability (lower the MFE relative to the reference sequence), while 12,061 SNVs had no predicted MFE change. An additional 15,019 variants were predicted to destabilize the G4. Transversions were more likely to change the structure of the G4 region without disrupting the G stacks and increasing the predicted thermodynamic stability of the structure (17%) compared to transitions (10%). Transition mutations were predicted to destabilize the G4 structure at a higher rate (22.9%) compared to transversions (17.6%) (Table 1).

### 3.4. G4 Variants in Transcript Regions

We find more G4 mutations in exonic regions, the 5′ untranslated region (UTR), the 3′ UTR, and coding sequence (CDS) regions of protein-coding genes when the G4 is formed in the strand opposite the transcribed gene (Figure 3c), consistent with previous studies on the role that G4s play in transcription [54,55,56,57,58,59,60,61,62,63,64,65]. The number of SNVs around G4 forming regions in introns and promoters was proportionate with the transcript in both the same and opposite strands. This suggests a selective pressure of variants around exon regions compared to non-coding regions. Previously, it was hypothesized that the formation of G4 in either strand within the transcribed region along with nascent RNA would lead to the formation of DNA:RNA hybrid R loops in the G4, which results in physically halting the polymerase movement inhibiting further rounds of transcription [66]. Additionally, G4 formation on the non-template strand could interfere with the reannealing of the DNA strands, increasing the stability of the R loop hybrid [66].

### 3.5. G4 Variants in Gene Features

G→A mutations are elevated in exons (35.21%) and show a decrease within promoters (22.2%). We find a lower percentage of T→G mutations in G4 regions occurring in exons (11.71%) compared to intron (22.23%), promoter (21.79%), enhancer (32.26%), and intergenic regions (18.29%). This pattern of low T→G variants coincides with counts in the CDS region, while the 5′ UTR shows increased T→G variants (17.94%). G→A SNVs are found less in enhancers (21.94%) distant from the transcription site. Deamination occurring upstream of the transcription site does not appear to affect the G4 region. These regions show the highest proportion of T→G (32.26%) mutations (Table 2).

Previously, higher counts of C→T over G→A variants were identified in the non-template strand, which was hypothesized due to cytosine deamination in the nearby 2 kb downstream of the 5′ end of genes due to a higher exposure of single-stranded DNA [67]. However, we hypothesize that these variants cause a conformational shift in the G4 structure, leading to an alteration in expression and binding patterns across these regions. Additionally, 8-oxoG formation, which is formed via the oxidation of guanine and frequently leads to G→A base pairing, leading to an eventual G→T transversion, binds Sp1 proteins in G4s and is an important regulator for adipose tissue development. The GC-rich promoter region with Sp1 transcription factor sites activates proportional to increasing 8-oxoG abundance [68].

**Figure 5:**
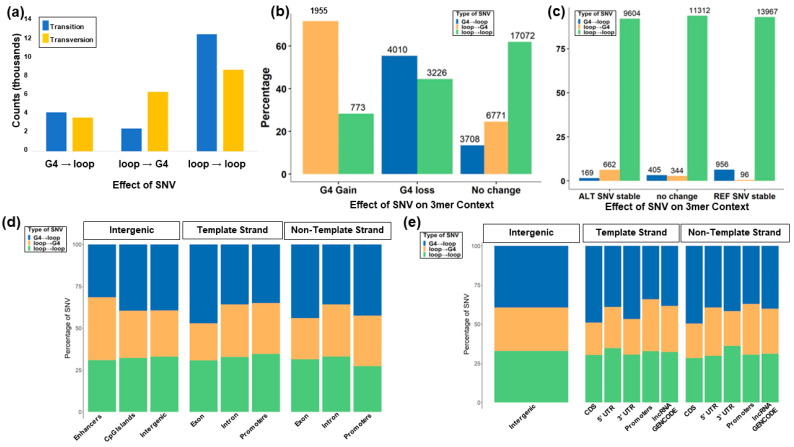
Effect of variants causing a gain or loss of a G4. (**a**) Effects of transition versus transversion mutations indicate that transitions are more likely to lead to a G4 loss, while transversions are more likely to lead to a G4 gain. (**b**) Effect of SNVs in a 3-mer context leading to G4 gain (left), loss (center), or no change (right). (**c**) Effect of SNVs in a 3-mer context shows that variants are more likely to be found in loop regions (green). (**d**) Breakdown of mutations causing different conformations in enhancers, CpG islands, intergenic regions, exons, introns, and promoters on both the template and non-template DNA strands. (**e**) Breakdown of mutations causing different conformations in intergenic regions, CDS, 5′UTR, 3′UTR, promoters, and lncRNA on both the template and non-template DNA strands.

G quadruplexes in the 3′ UTR occurring on the same strand of the coding region are prone to variants cataloged in COSMIC that are predicted to either increase or decrease stabilization based on MFE measures. We investigated which SNVs in each annotation had the highest change in terms of specific nucleotide substitutions. The 3′ UTR has a higher incidence of T→G versus A→G SNVs. This suggests that T→G mutations are more likely to stabilize 3′UTR G4s. Putative G4s in CDS and CpG regions are least prone to variants, while enhancers and the intergenic G4 show higher changes in stabilization (both stabilizing and destabilizing) due to the SNVs (Figure 5a,b, Supplemental Figure S1).

### 3.6. Enrichment Analysis

#### 3.6.1. Gene Ontology

A Gene Ontology (GO) enrichment analysis was performed for biological processes (GO:BP) and cellular components (GO:CC). A total of 424 GO:BP categories were determined to be significant (FDR ≤ 0.05) using both datasets (Supplemental Figure S2; Supplemental Table S5), while 425 significant GO:BP enrichments were found for COSMIC alone (Supplemental Figure S3; Supplemental Table S6) and 48 were found for CLINVAR (Supplemental Figure S4; Supplemental Table S7). When this was further broken down into mutations resulting in a loss of a G4, we found 205 significant GO:BP for both (Supplemental Table S8), 75 for COSMIC (Supplemental Table S9), and 25 for CLINVAR (Supplemental Table S10). Among the COSMIC enrichments were synapse organization, axonogenesis, neuron projection guidance, axon guidance, cell–substrate adhesion, neuromuscular process, regulation of neuron projection development, and xenobiotic glucuronidation. One example gene is the App transcript, which is involved in synapse formation and function in the developing brain. This transcript is transported to neuronal dendrites, where the transmembrane APP protein plays an integral role in synapse formation and function. However, the translation of App is repressed by the binding of the fragile X mental retardation protein (FMRP) to G4s in the App coding region. This repression is thought to occur through a direct interaction with the ribosomes, resulting in stalled ribosomal progression on the mRNA [69]. Past studies have also shown that this repression can be relieved via the synaptic activation of metabotropic glutamate receptors, specifically mGluR5 receptors. This results in the release of FMRP and an increase in APP translation [70].

The complete set of CLINVAR enrichments included muscular-related processes, such as striated muscle contraction, a neuromuscular process, cardiac muscle cell contraction, muscle tissue morphogenesis, regulation of action potential, cell communication involved in cardiac conduction, regulation of heart rate by cardiac conduction, musculoskeletal movement, cardiac muscle tissue morphogenesis, skeletal muscle contraction, and ventricular cardiac muscle cell action potential. Variants leading to a gain of a G4 result in 115 GO:BP enrichments for both (Supplemental Table S11), 22 for COSMIC (Supplemental Table S12), and 2 for CLINVAR (Supplemental Table S13). Among the COSMIC enrichments from the G4 gain are the positive regulation of transcription by the RNA polymerase II and actin cytoskeleton organization, while the CLINVAR enrichments based on a G4 loss include system development, action potential, and cardiac muscle cell action potential. The loss of the G4 using COSMIC resulted in similar enriched GO terms as did G4 loss in CLINVAR, including muscle contraction, the muscle system process, the heart process, the cardiac muscle cell action potential involved in contraction, actin-mediated cell contraction, the regulation of heart contraction, and action potential.

Among the enriched categories detected were PDZ domain proteins (GIPC2, GRIDZIP, LIMK2, PDLIM7, PDZD7, WHRN, SIPA1L3, PRX, MYO1BA, MAGI2, and MAST) with the G4 in coding regions and variants that negatively affect the RGG (arginine–glycine–glycine) domain or the G4 stability. Proteins with RGG repeats have been known to bind to G4 structures [71]. Variants in these regions affecting the G4 stability could further affect downstream binding.

GO:CC enrichments yield 128 significant categories for both datasets combined (129 for COSMIC only and 14 for CLINVAR only) (Supplemental Figures S5–S7; Supplemental Tables S14–S16). Among the enriched GO:CC categories detected in COSMIC are the collagen-containing extracellular matrix and the cell–cell contact zone, indicating that mutations in these genes affect the adhesion of cells to the extracellular matrix. Other enriched GO:CC terms in CLINVAR include I band, sarcolemma, and the myofilament Z disc. Enriched GO:CC terms from a loss of the G4 using the CLINVAR database include a collagen trimer and a PCSK9-LDLR complex.

#### 3.6.2. KEGG Metabolic Pathways

KEGG enrichment yielded 96 significant pathways for the combined datasets, 91 COSMIC, and 11 CLINVAR (Supplemental Figures S8–S10; Supplemental Tables S17–S19). Those leading to a loss of the G4 yielded 33 significant categories, including 12 and 5 for COSMIC and CLINVAR, respectively (Supplemental Tables S20–S22). Among the enriched categories for genes with a loss of the G4 within CLINVAR are hypertrophic cardiomyopathy, dilated cardiomyopathy, arrhythmogenic right ventricular cardiomyopathy, adrenergic signaling in cardiomyocytes, and acute myeloid leukemia. KEGG enrichments for a gain of the G4 resulted in 31, 3, and 0 for combined, COSMIC only, and CLINVAR only, respectively (Supplemental Tables S23 and S24). The enriched terms from the gain of the G4 in CLINVAR variants are melanoma, the phospholipase D signaling pathway, and cocaine addiction.

#### 3.6.3. INTERPRO Protein Domains

INTERPRO enrichment yielded 23, 23, and 2 enrichments for combined CLINVAR and COSMIC variants, COSMIC only, and CLINVAR only, respectively (Supplemental Tables S25–S27). Included were the Src homology-3 domain (n = 69 FDR = 3.73 × 10^−3^) and the Pleckstrin homology-like domain (PH) (n = 147, FDR = 1.30 × 10^−10^). The binding affinity of PH domains has unique recognition sites and is known for functional plasticity [72,73].

#### 3.6.4. Transcription Factors

**Figure 6:**
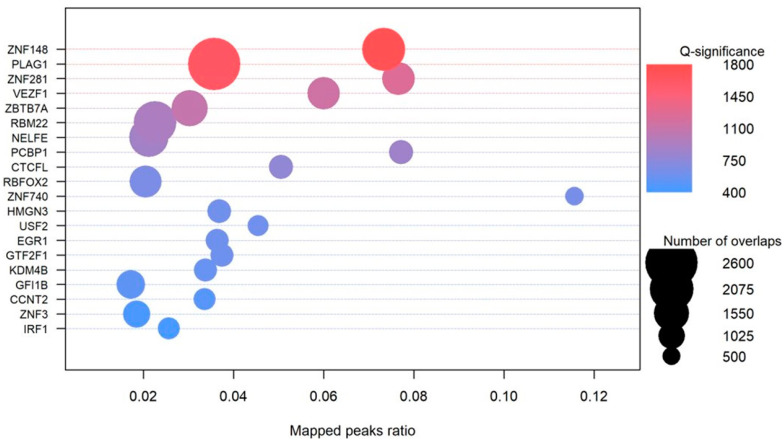
Significance of the top 20 transcription factors and their genome-wide binding sites.

We identified the enrichment of TFs including NFKB1, ZFX, MBD3, ASX1, SUZ12, NCOR1, HMGN3, USF2, EGR1, GTF2F1, KDM4B, HNRNPH1, HNRNPL, NONO, TARDBP, NFATC3, KDM3A, and HOXA3, among others (Figure 6; Supplemental Figures S11–S13; Supplemental Table S28). The majority (92%) of these had at least one G4 in their gene structure working in a feed-forward regulation of genes. A number of the SNPs found in these regions disrupt the recognition motifs for transcription-binding sites.

### 3.7. Trinucleotide Context Mutation in the G4 Sequence

**Figure 7:**
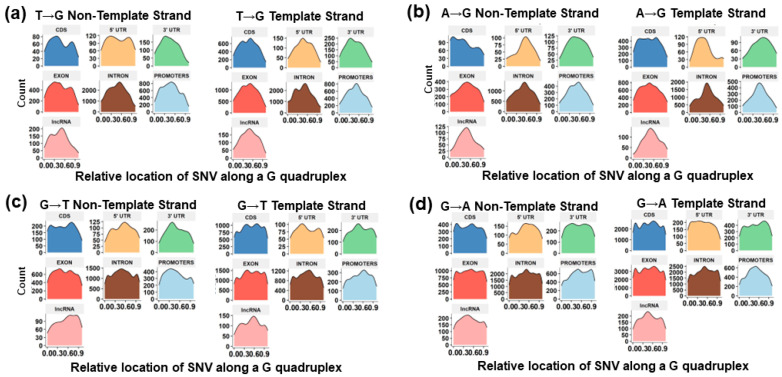
Distribution of SNVs across the G4 regions on the non-template and template strands. Shown are the results for (**a**) T→G variants, (**b**) A→G variants, (**c**) G→T variants, and (**d**) G→A variants.

**Figure 8:**
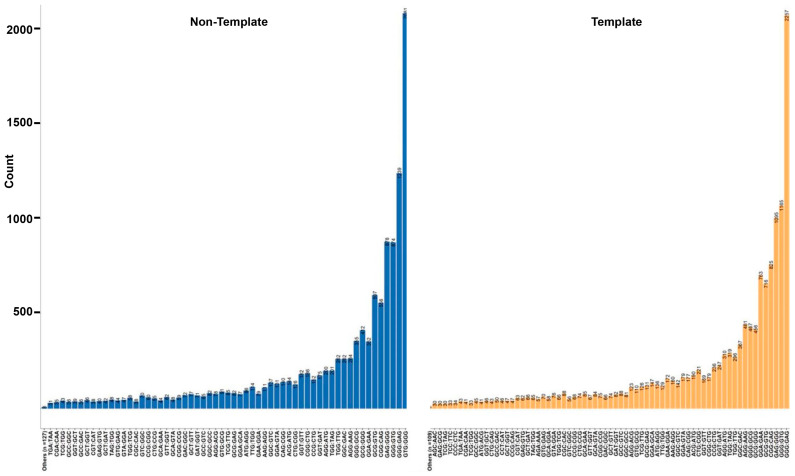
Distribution of SNVs in trinucleotide contexts relative to the opposite or same strand as the corresponding gene.

Based on the nucleotide context of one base pair before and after the mutation, we identified 79% of the variants to be affecting the loop region and 23% of the SNV after the change leads to the formation of GGG in regions with G(A|C|T)G (Figure 4 and Figure 7, Supplemental Figure S14). We find that 36% (n = 6810) of the transversion mutations are T→G, while 21% (n = 4070) of the transitions are A→G (Figure 7a–d). T→G mutations occurring in the context of GTG→GGG occur in 14% of the SNVs, leading to the formation of a stable G tetrad, while GAG→GGG occurs in 6% of the variants. Interestingly, the destabilization of the GGG region occurs via the GGG→GAG transition in 11% of SNVs (Figure 8). Previously, it has been reported that the GGG exhibits context-dependent specific mutational patterns that preserve the potential for G4 formation [74]. We find G→A mutations to be approximately 29% of the total SNVs in the selected G4 regions, with 26% (n = 4144; 11% of the total) of those variants occurring in the context of GGG→GAG (Figure 8). We observe these patterns in non-coding annotations with the exception of exonic and CDS regions. We identify an increased propensity to form stable multiple conformations with de-stabilized structures for 25% of the sequences with variants, while 14% of the variants have no predicted change in stability (Supplemental Table S29). This approach of analyzing the probable base pairing alternatives for additional guanine Hoogsteen base pairing can help identify the effects of variants within the G4 structure and hence predict the change in structure and functionality of G4s in various molecular processes.

Based on the position of the mutation in the G4, the normalized position for each variant was calculated. The relative location of a variant in a G4 is defined as the position of the variant divided by the length of the G4. For single nucleotide variants mutating to G either from A or T, we find similar elevated patterns in the middle of the G4. T|A→G mutations show conservation of guanine in the center position except for CDS and exons in both the template and non-template strand across both COSMIC and CLINVAR databases (Figure 8, Supplemental Figures S15–S18). These changes are stricter for SNVs within the 5′UTR across the template and non-template strand in the CLINVAR database, where we observe mutations in the center of the G4 for T→G variants. The 5′ UTR COSMIC mutations show mutations across the two extreme loops compared to the center. A→G mutations are observed in a higher proportion at the beginning of G4s in the CDS region, which provides evidence for selective pressure in the coding region preferentially protecting the coding sequence. G4s in UTRs have been reported to be under selection pressure, and variants in the G4 can account for the instability in the G4 and diseases [30], indicating an important functional role leading to conservation both within [63,75] and across species [34,56,76,77].

### 3.8. Role of the Location of SNVs in G4s

The relative position of G→T substitutions along G4 sequences is shown in Figure 7c. The location of this mutation at the beginning of the G4 can disrupt the structural formation; however, further elevated peaks at varying locations leading to additional guanines across the G4 may introduce additional tetrads in introns and exons (Figure 5c,d).

The observation of increased guanine stacks resulting from G→T|A substitutions that break up longer guanine runs is consistent with studies where the oxidation of multiple G’s occurs at the start of the guanine tetrads [29,78]. Our results help establish that the location of mutations and the type of mutation in G-rich regions likely alter the shape and stability of the G4 structure. Previously, it has been established that the most sensitive sites are located at the center tetrad [79]. For mutations in CLINVAR, we observe a higher mutation rate at the start of the G4 (Supplemental Figure S19). The A→G mutations associated with COSMIC variants show a considerable difference in their location relative to the G4 position (Supplemental Figure S20).

## 4. Discussion

### 4.1. Molecular Mechanisms for Promoting Mutations in G4 Regions

High occurrences of oxidized guanines in G4 structures have been previously established [29]. This type of mutation is thought to occur around the external tetrads due to radical-trapping antioxidants that slow mutation efficiency [78]. The oxidative stress occurring due to the reactive oxygen species (ROS) affects the genome stability and promotes mutagenesis, senescence, and other age-related diseases [80]. Mutations in GGG regions can destabilize the stacking of guanines, altering the ionization potential and affecting the ability of the G region to be further oxidized. G→A, T, or C mutations can disrupt the stacking, while mutations to G can further stabilize the G4 or allow additional conformations for the stacking. We investigated the change of each type of SNV in each annotation to have the highest change. Based on the absolute ∆MFE, we find that G4s in the CDS and CpG regions are the least likely regions to be affected by mutations, while enhancers and intergenic G4s are prone to higher variant-induced stabilization and destabilization (Figure 5a,b).

The escape of 8-oxoG from DNA repair during DNA replication can cause the misincorporation of adenine opposite 8-oxoG, leading to the addition of T in place of G (OG mutations) [81,82]. For instance, in a sequence with GTTAGGG with 8-oxoG at its fifth position, a mis-incorporation of the A occurs opposite G. Due to the presence of consistent Gs in the region, the true proportions of change in these regions can be hard to monitor over a range of replications. The methylation of cytosine leads to the formation of 5-methyl cytosine, which is a residue for spontaneous transitions [83]. Cytosine deamination might be the primary cause of C→T transition. Further, based on the context, a high proportion of T→G mutations lead to a GTG→GGG structure, supporting the stability of the G4. It presents a question of whether T→G mutations confer additional stability of the G4 in cancer cells. Past studies have highlighted the conditional impact of OG mutations in a base pairing with A in mutagenic MutY homolog harboring increased G→T transversions in MUTYH, leading to a higher incidence rate of colorectal cancer [84,85,86]. Thymine glycol is a lesion that is highly mutagenic and cytotoxic in regions of DSBs. In vitro studies have shown it to block replicative and repair DNA polymerases [87]. The OG, thymine glycol, and abasic sites formed are repaired by the excision repair pathway. The difference in the repair of 8-oxoG sites has been observed in NEIL glycolysis, which has been known to remove guanidinohydantoin (Gh) and spiroiminodihydantoin (Sp) from G4 structures in the promoter region over parallel conformation [31]. However, the glycolysases were not able to remove the 8-oxoG structure from the telomeric G4 or the same G4 structure in antiparallel structures.

We identified an increase in the presence of A|T→G mutations in the middle stacking of the G4, suggesting a functional impact for specific variants in G4 formation. G4s with spare tires (i.e., additional guanine tracts [88]) allow for alternate G4 structures to form, as does the exclusion of certain guanosines due to lesions or substitution in one tetrad region that might break apart longer guanine runs. A base excision repair with APE1 and OGG1 in the promoter region of VEGF has been implicated in G4 formation and may be involved in other genes as well [89,90].

### 4.2. TERT G4 Mutations

A prior study highlighted that the entire 67 bp G4 associated with the TERT promoter was protected from DNase cleavage, while the version containing G→T variants was found to be degraded into discrete segments [32,91]. Additionally, this region folds into a compact G4 structure without any hairpins in between the guanine stacks. However, based on DMS footprinting studies, the formation of hairpins has been predicted [92]. We identified 52 possible SNVs in 39 base pair locations in this 67 bp G4. The SNV chr5:1,295,113 (G→T) located in the TERT region is present around a G4 in the non-template strand. The SNV was associated with more than twenty-two cancer types, including the central nervous system, liver, bladder, ovarian, breast, kidney lung, bone, and pancreatic, among others. Many of these SNVs destabilize G4s. Further, with nine tetrads (GGG repeats present), multiple G4s can potentially be formed. With a SNV (G→A), we find the stability with the variant to differ if alternate G4 tetrads are used for the stacking.

### 4.3. G4 Mutations Disrupting the Transcription Factor Binding

Transcription factor proteins (TFs) known to bind G-rich regions, including SP1 [93], NF-κB [94], CREB [95], and the methyl-CpG binding domain MBD of methyl-CpG binding protein 2 (MeCP2) [96] had decreased association constants up to 10-fold for transcription factor sites with a change of guanine to 8-oxoguanine in model duplex DNA with the donor–acceptor pattern change on the imidazole ring in guanine compared to OG. The structure change for guanine in association with CREB was found to have a role in epigenetic repression [95]. This is supported by our results highlighting the reversal of these sequences to a stabilized G4 via a change through the T→G region in cancer cells. For instance, the variant chr10:122,143,482: G→A significantly affects the binding sites of TFs NHLH1, FOXO3, TAL1, TP53, HES5, HES7, USF2, EGR3, ZNF740, and SP1, among others (Supplemental Table S30). We observe similar observations for an additional 424 SNVs, which occur in at least five cancer types in the COSMIC database and disrupt the TF binding site with an average of 15.1 TF per variant.

A local network cluster (STRING) analysis of the enriched TFs yielded terms related to the Polycomb repressive complex (PRC1) (4/12 FDR 0.00049), the PcG protein complex (6/25, FDR 6.22 × 10^−6^), and the positive regulation of histone H3-K27 methylation (11/59 FDR 1.82 × 10^−10^). The PRC1 engages in transcriptional control through chromatin modification with histone 2A through a protein ligase ubiquitylation [97,98]. Although the mechanism of the PRC1 is under active investigation, recent evidence suggests that G tracts selectively remove the PCR2 complex from genes during gene activation [99]. Polycomb complexes have been associated with repression to maintain cell identity but are associated with actively transcribed loci, and this evidence suggests a direct role of G4s across cell types to regulate expression through structural variation [99,100].

Repair mechanisms, including BER and mismatch repair, are required to protect non-canonical or mismatched base pairs due to a polymerase error [101,102,103,104]. Neurogenerative disorders occurring through the expansion of CAG→CTG repeats have been associated with MutSβ, a heterodimer involved in mismatch repair [105,106]. Though the involvement of G4s in gene transcription [58,61,62,64,65,89,107] and telomere regulation [16,17,29,35,108] is well studied, the mechanism of base excision repair by DNA glycosylases in G4s and other non-canonical structures is poorly understood [109,110]. We identified G4s with SNVs in the genes of CHRNG, GRIN2C, CHAT, ADCY1, GABRG3, CACNG3, PPFIA3, LRTOMT, VAMP2, TSPOAP1, MAPK3, GABRR2, KCNJ6, PICK1, and STX1A, among others. These genes have been associated with several psychiatric disorders, including schizophrenia, bipolar disorder, tobacco use disorder, Parkinson’s disease, and autism [111,112,113,114,115,116].

Previous research has shown the presence of G4 sequences in various untranslated dendritic mRNAs, suggesting the role of G4s as a neurite localization signal [117,118]. The deletion of different putative G4 sequences led to a severe loss of signal in neurites [119]. It has been hypothesized that the G4 structure, being sensitive to cationic, can function in correlation to the neuronal activity in localization and transport as activity-dependent changes [120,121]. Cationic sensitivity could influence the stability and structure and regulate the binding of trans-acting factors [118].

## 5. Conclusions

G4s are formed due to the intricate balance between the folding energy by a nick in the DNA [122], methylated guanines [123], and guanines available for stacking [124]. The balance between the hypomethylated and hypermethylated G-rich regions near promoters (despite cytosine deamination and cytosine methylation) results in preserved CpG islands across mammalian genomes [125], which are maintained by G4 structures that act to sequester DNMT1, contributing to the CpG hypomethylation [107]. These methylation and oxidation patterns result in G4 sequence preservation. In addition, cytosine deamination has been shown to have important roles in the G4 structure, including a destabilization in telomeres [126] and a class switch recombination in mature B cells [127].

With the introduction of next-generation techniques for the identification of G4s, an analysis of variants in these complex regions and the mechanism of formation of a G4 in different cell types remains uncertain. Our study points out genes and G4 sequences that are affected by either somatic or germline mutations. Of those variants identified, 37,515 are observed as somatic mutations associated with cancer, while 4999 are germline mutations of clinical significance. We identify the possible effects of these single nucleotide variants occurring within coding and non-coding regions on the stability of G4s.

## Figures and Tables

**Table 1:** Count and proportion of the effect of the type of mutation on the stability of G4 (COSMIC database).

Type of SNV	Effect of SNV on MFE	Freq	Percentage
Transition	Destabilized	8600	22.93
Transversion	Further stabilized	6603	17.60
Transition	No change	6552	17.46
Transversion	Destabilized	6419	17.11
Transversion	No change	5509	14.68
Transition	Further stabilized	3832	10.21

**Table 2:** Proportion of SNV via annotation.

SNV	3′ UTR	5′ UTR	CDS	CpG Islands	Enhancers	Exon	Intergenic	Intron	lncRNA GENCODE	Promoter
G→A	34.82	31.23	39.34	27.58	19.01	35.18	27.84	26.74	28.27	26.87
G→T	18.3	14.82	15.84	12.01	12.08	16.6	17.15	14.56	14.89	13.64
C→T	11.13	8.24	12.19	12.23	5.68	11.35	7.94	9.06	10.46	8.89
T→G	12.67	16.48	8.59	16.78	29.84	11.74	18.38	19.91	18.15	18.95
A→G	7.62	10.83	6.91	9.56	12.61	8	11.38	11.49	9.89	10.98
G→C	5.16	6.05	4.69	6.27	7.28	5.09	6.89	6.3	6.23	6.51
C→G	3.66	4.85	4.47	7.69	6.75	4.33	3.47	4.59	4.56	6.34
C→A	2.76	2.99	4.11	3.85	1.42	3.65	1.95	2.6	2.73	3.3
T→C	2.15	1.66	1.79	2	2.13	1.96	1.67	2.1	2.13	2.07
T→A	0.7	0.86	0.84	0.67	0.89	0.81	1.27	1	1.17	0.82
A→T	0.59	1.2	0.72	0.75	1.6	0.76	1.12	0.94	1	0.85
A→C	0.45	0.8	0.51	0.62	0.71	0.52	0.94	0.72	0.53	0.77

## Data Availability

All code and resulting data are available in the GitHub repository (https://github.com/UofLBioinformatics/G4_SNV (accessed on 24 November 2023)). UCSC Genome Brower tracks are available at https://bit.ly/G4_SNV (accessed on 24 November 2023).

## Supplementary Materials

The following supporting information can be downloaded at: https://www.mdpi.com/article/10.3390/genes14122125/s1, Figure S1: Effect of each SNV on ∆MFE of G4 on different annotations with percentage of the counts shown in the secondary y axis; Figure S2: Top 25 enriched GO:BP terms for COSMIC and CLINVAR G4 mutations; Figure S3: Top 25 enriched GO:BP terms for COSMIC G4 mutations; Figure S4: Top 25 enriched GO:BP terms for CLINVAR G4 mutations; Figure S5: Top 25 enriched GO:CC terms for COSMIC and CLINVAR G4 mutations; Figure S6: Top 25 enriched GO:CC terms for COSMIC G4 mutations; Figure S7: Top 25 enriched GO:CC terms for CLINVAR G4 mutations; Figure S8: Top 25 enriched KEGG terms for COSMIC and CLINVAR G4 mutations; Figure S9: Top 25 enriched KEGG terms for COSMIC G4 mutations; Figure S10: Top 25 enriched KEGG terms for CLINVAR G4 mutations; Figure S11: Top 20 enriched transcription factors with overlapping ChIP-seq peaks for COSMIC G4 SNVs in the HEK293 cell line; Figure S12: Top 20 enriched transcription factors with overlapping ChIP-seq peaks for COSMIC G4 SNVs in the K562 cell line; Figure S13: Top 20 enriched transcription factors with overlapping ChIP-seq peaks for COSMIC G4 SNVs in the Hep-G2 cell line; Figure S14: Frequency of SNVs across G-quadruplex regions within trinucleotide contexts for the CLINVAR database; Figure S15: Distribution of A→G SNVs across the G4 region for different features on (A) the non-template and (B) template strand for CLINVAR variants; Figure S16: Distribution of G→T SNVs across the G4 region for different features on (A) the non-template and (B) template strand for CLINVAR variants; Figure S17: Distribution of G→A SNVs across the G4 region for different features on (A) the non-template and (B) template strand for CLINVAR variants; Figure S18: Distribution of T→G SNVs across the G4 region for different features on (A) the non-template and (B) template strand for CLINVAR variants; Figure S19: Distribution of SNVs across G-quadruplex regions for the (A) forward and (B) reverse strands for SNVs detected in the CLINVAR database; Figure S20: Distribution of SNVs across G-quadruplex regions for the (A) forward and (B) reverse strands for SNVs detected in the CLINVAR database by specific SNV substitution; Table S1: Count of all observed SNVs in the COSMIC database; Table S2: Counts of SNVs in G4 regions from the COSMIC database; Table S3: Changes in putative G4 from the COSMIC database across both strands before and after mutation; Table S4: Count and proportion of variants in experimentally validated G4 regions for different functional regions; Table S5: Significant GO:BP enrichments for all COSMIC and CLINVAR G4 mutations; Table S6: Significant GO:BP enrichments for all COSMIC G4 mutations; Table S7: Significant GO:BP enrichments for all CLINVAR G4 mutations; Table S8: Significant GO:BP enrichments for COSMIC and CLINVAR G4 mutations leading to the loss of a G4; Table S9: Significant GO:BP enrichments for COSMIC G4 mutations leading to the loss of a G4; Table S10: Significant GO:BP enrichments for CLINVAR G4 mutations leading to the loss of a G4; Table S11: Significant GO:BP enrichments for COSMIC and CLINVAR G4 mutations leading to the gain of a G4; Table S12: Significant GO:BP enrichments for COSMIC G4 mutations leading to the gain of a G4; Table S13: Significant GO:BP enrichments for CLINVAR G4 mutations leading to the gain of a G4; Table S14: Significant GO:CC enrichments for COSMIC and CLINVAR G4 mutations; Table S15: Significant GO:CC enrichments for COSMIC G4 mutations; Table S16: Significant GO:CC enrichments for CLINVAR G4 mutations; Table S17: Significant KEGG enrichments for COSMIC and CLINVAR G4 mutations; Table S18: Significant KEGG enrichments for COSMIC G4 mutations; Table S19: Significant KEGG enrichments for CLINVAR G4 mutations; Table S20: Significant KEGG enrichments for COSMIC and CLINVAR G4 mutations leading to a G4 loss; Table S21: Significant KEGG enrichments for COSMIC G4 mutations leading to a G4 loss; Table S22: Significant KEGG enrichments for CLINVAR G4 mutations leading to a G4 loss; Table S23: Significant GO:CC enrichments for COSMIC and CLINVAR G4 mutations leading to a G4 gain; Table S24: Significant KEGG enrichments for COSMIC G4 mutations leading to a G4 gain; Table S25: Significant INTERPRO enrichments for COSMIC and CLINVAR G4 mutations; Table S26: Significant INTERPRO enrichments for COSMIC G4 mutations; Table S27: Significant INTERPRO enrichments for CLINVAR G4 mutations; Table S28: Top 50 significant transcription factor enrichments for COSMIC and CLINVAR G4; Table S29: Count and percentage of effect of SNV calculated by thermodynamic MFE and ED changes in the G quadruplex sequence; Table S30: Effect of transition mutation G→A in chr10:122,143,482 on potential binding for multiple transcription factors.
