# Supplementary material for: Analysis of Nucleotide Variations in Human G-Quadruplex Forming Regions Associated with Disease States
Source: Genes (Basel). 2023 Nov 25;14(12):2125. doi: 10.3390/genes14122125 (PMC10742762; doi:10.3390/genes14122125)
Supplement: Supplementary file 1 [file genes-14-02125-s001.zip › SupplementalFigures.pdf]

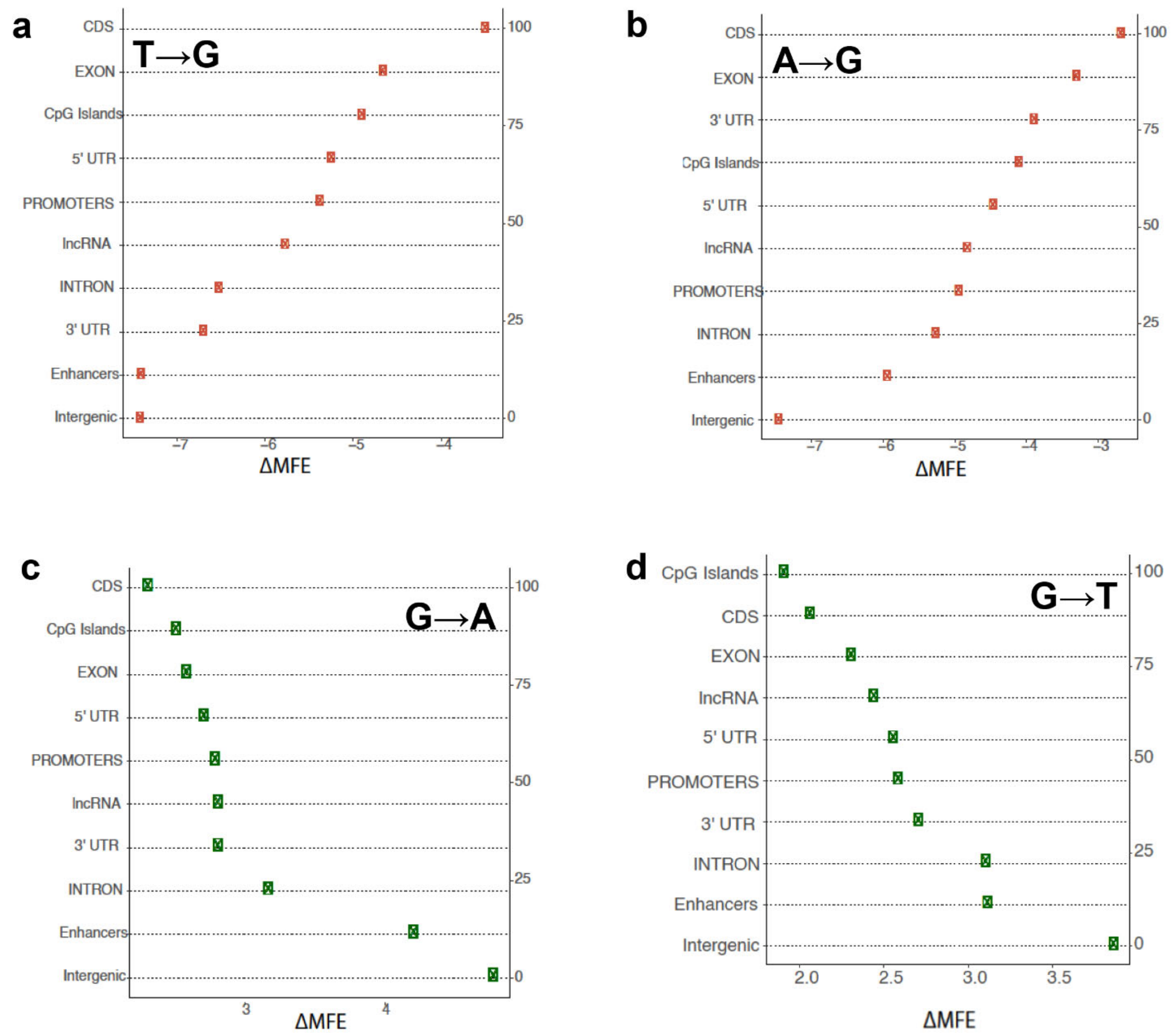

Figure S1. Effect of each SNV on ∆ MFE of G4 on different annotations with percentage of the counts shown in the secondary y axis. Shown is (a) T→G SNVs; (b) A→G SNVs; (c) G→A SNVs; and (d) G→T SNVs.

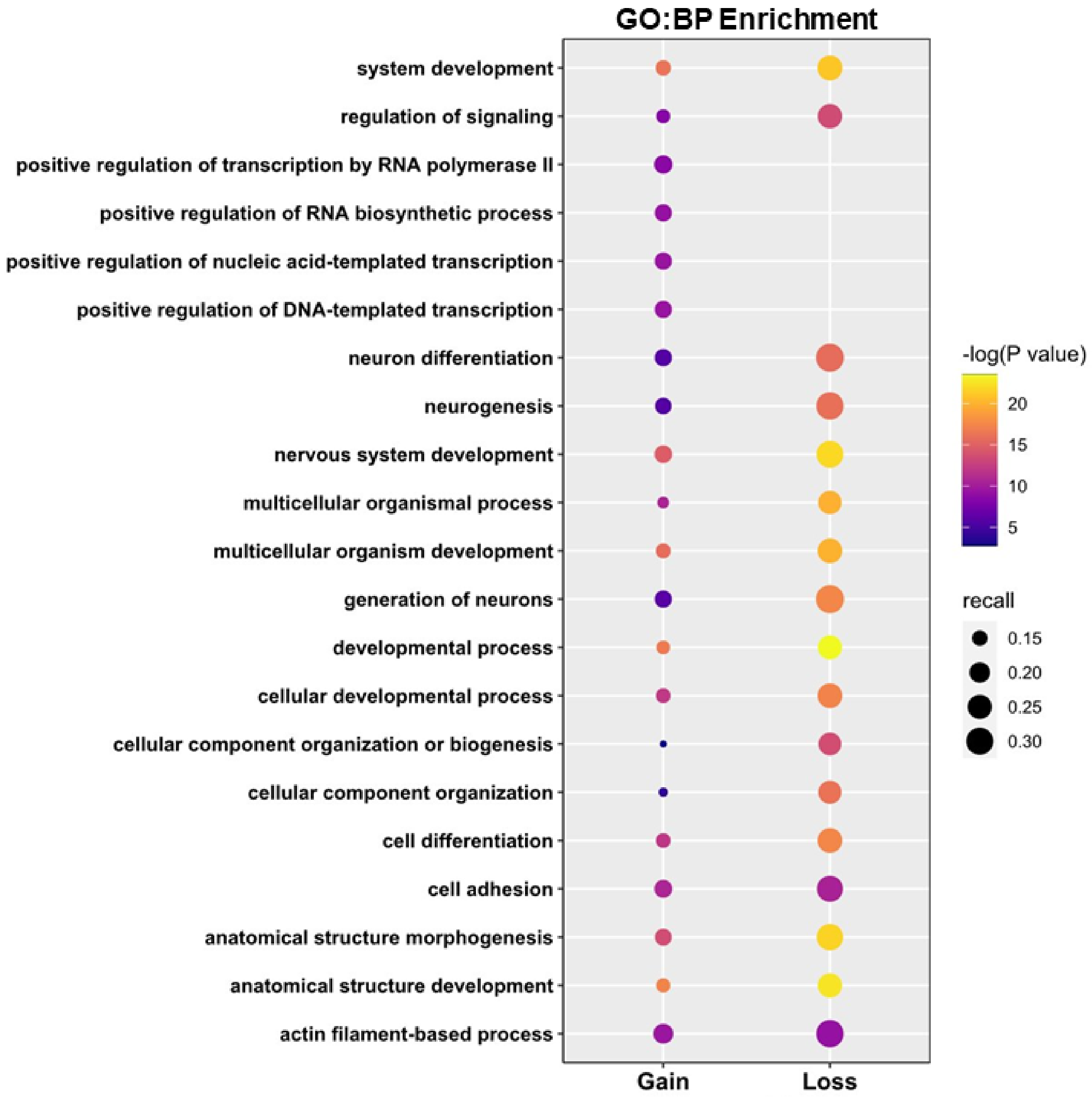

Figure S2. Top 25 enriched GO:BP terms for COSMIC and CLINVAR G4 mutations.

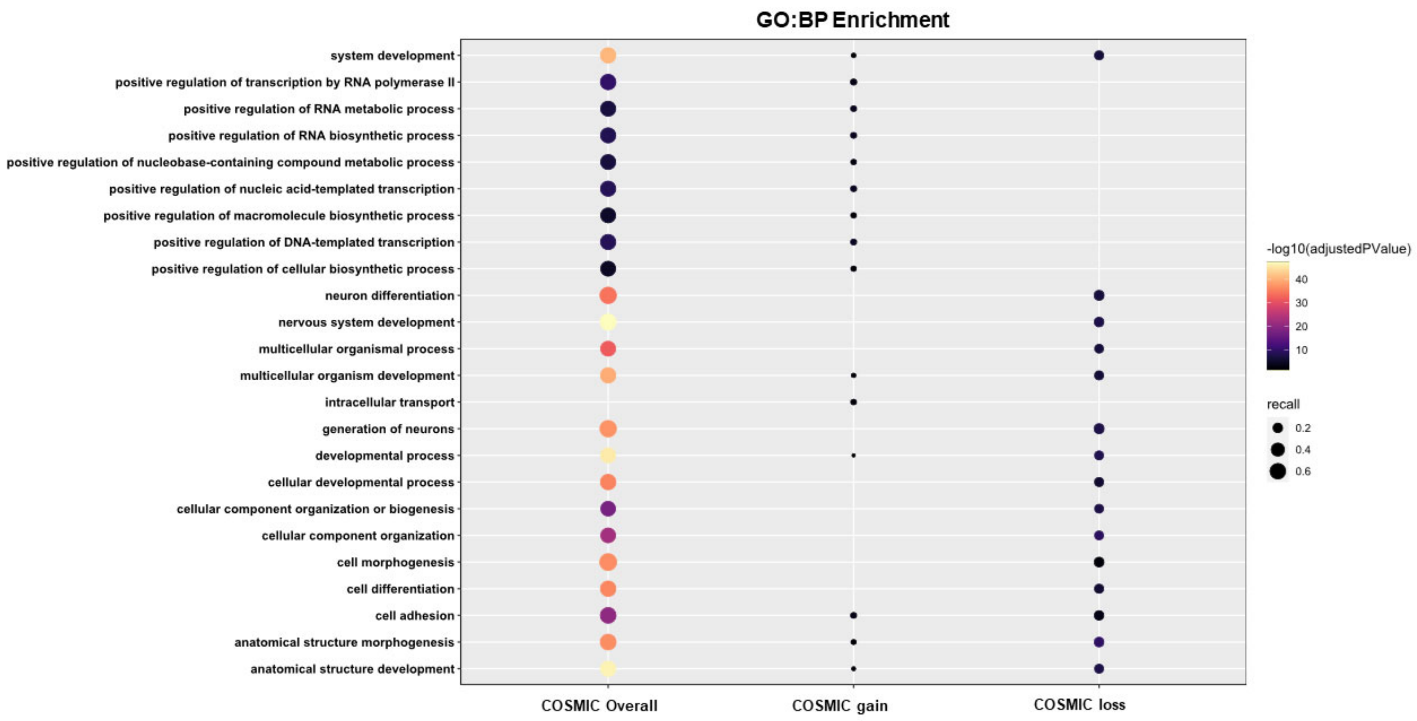

Figure S3. Top 25 enriched GO:BP terms for COSMIC G4 mutations.

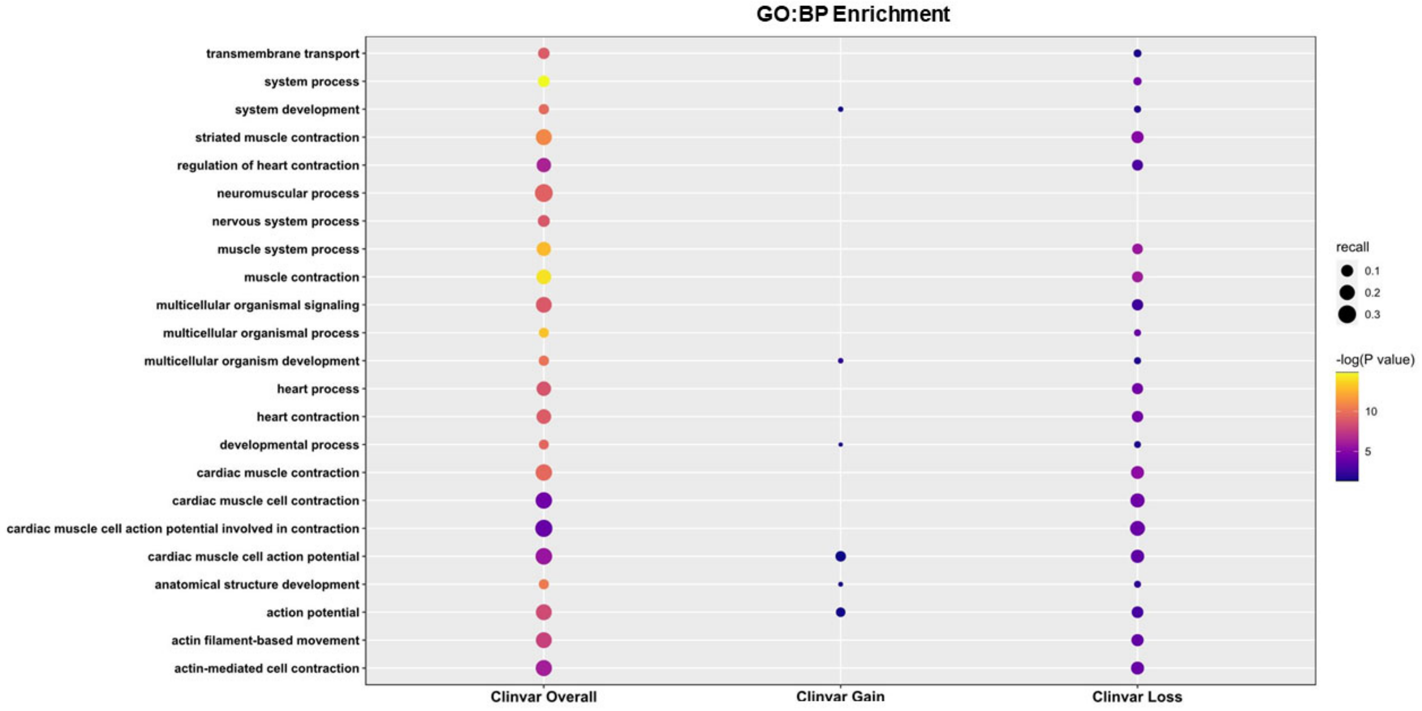

Figure S4. Top 25 enriched GO:BP terms for CLINVAR G4 mutations.

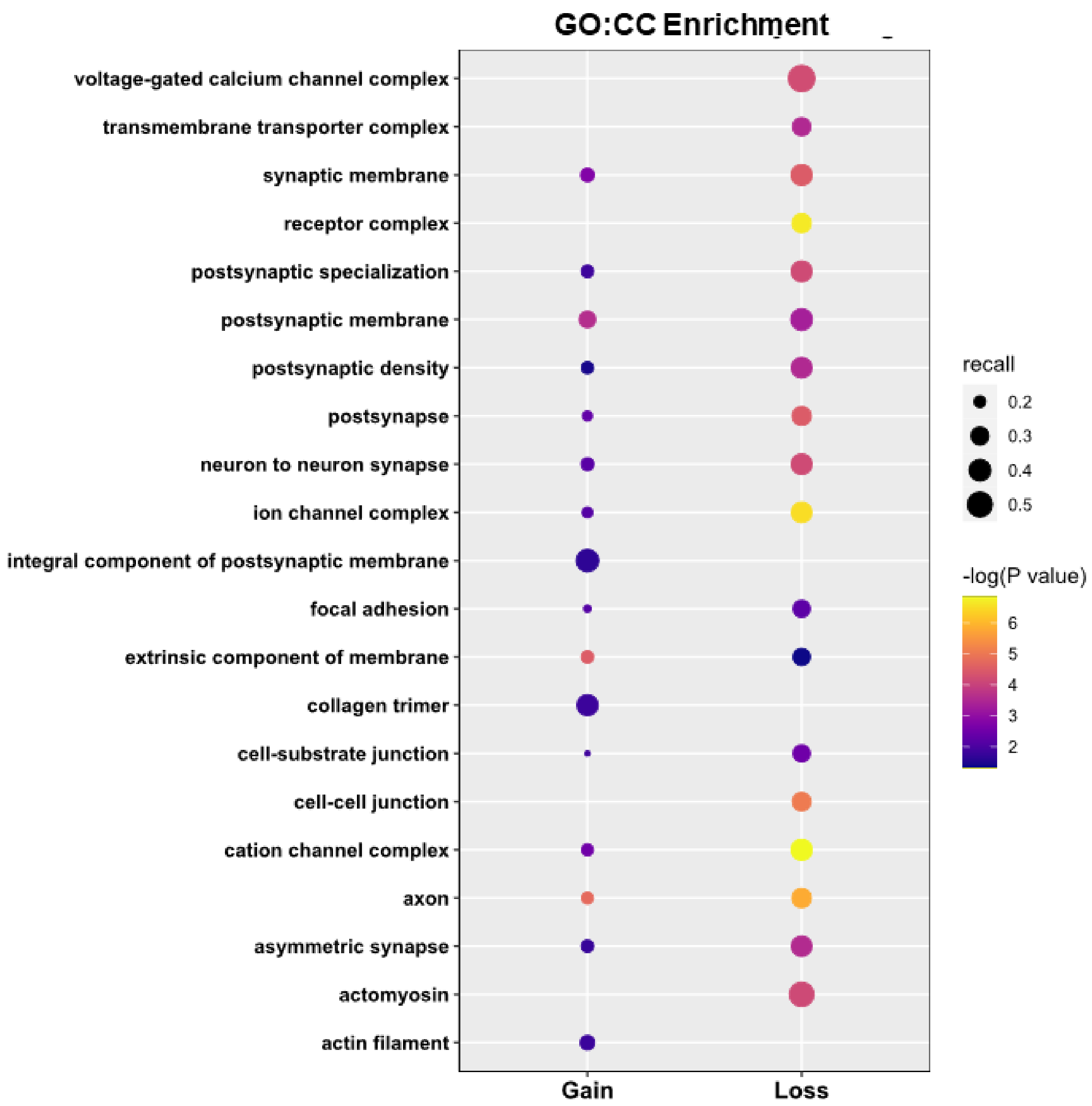

Figure S5. Top 25 enriched GO:CC terms for COSMIC and CLINVAR G4 mutations.

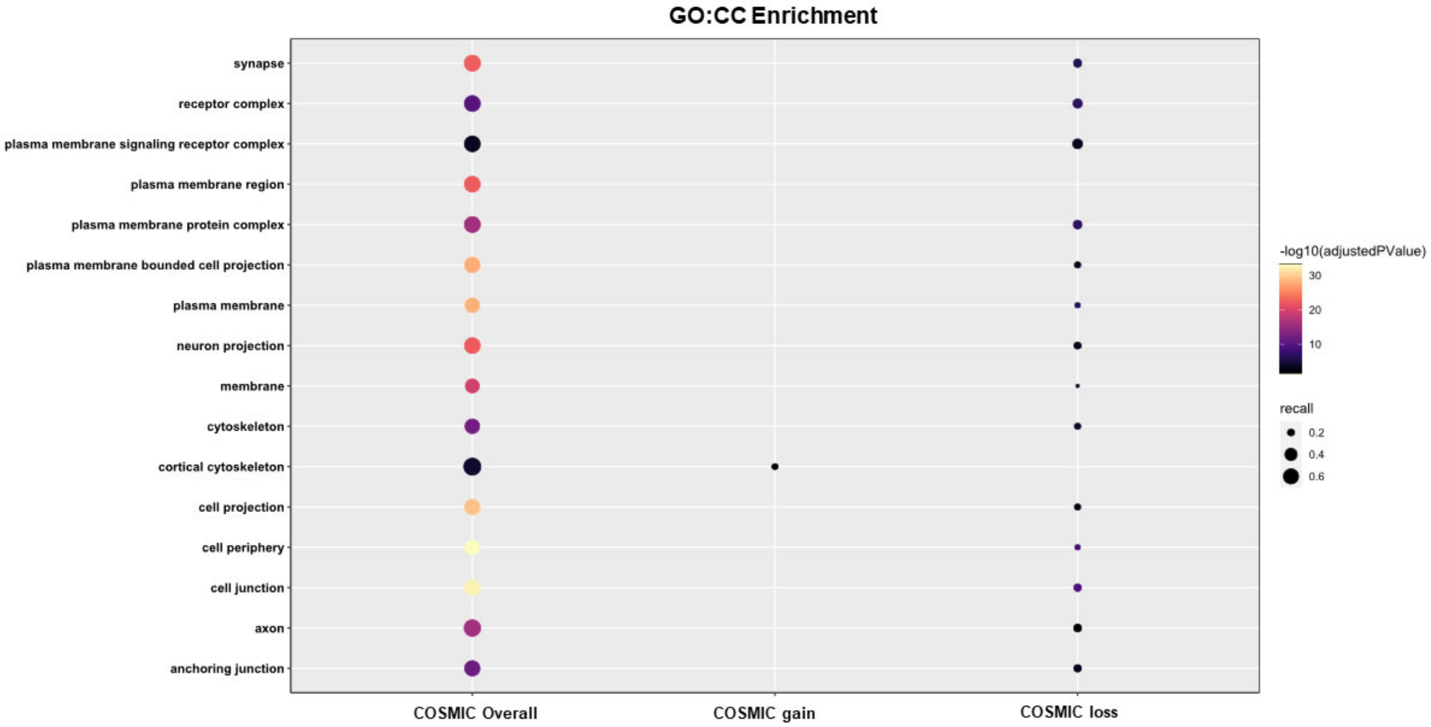

Figure S6. Top 25 enriched GO:CC terms for COSMIC G4 mutations.

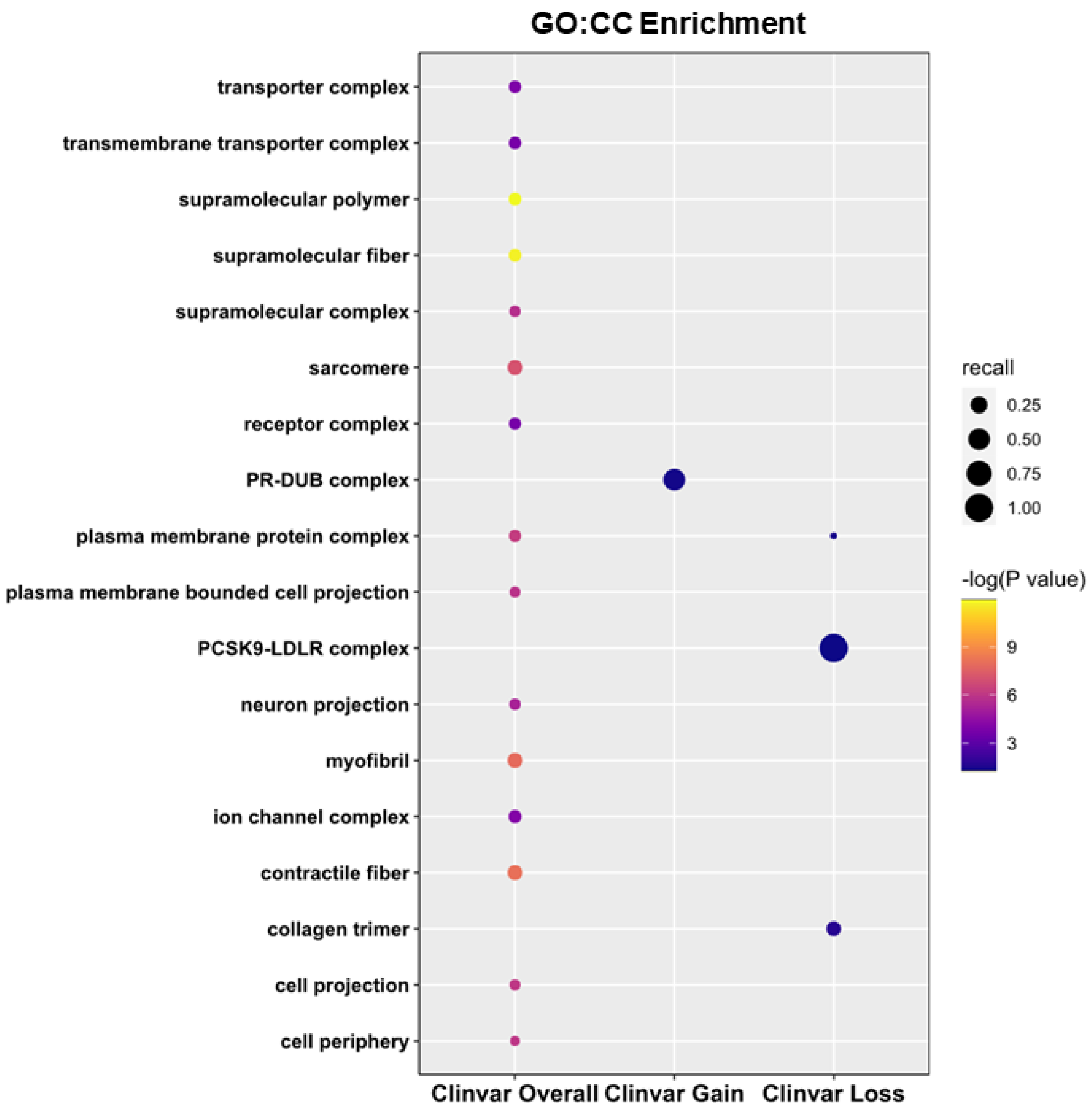

Figure S7. Top 25 enriched GO:CC terms for CLINVAR G4 mutations.

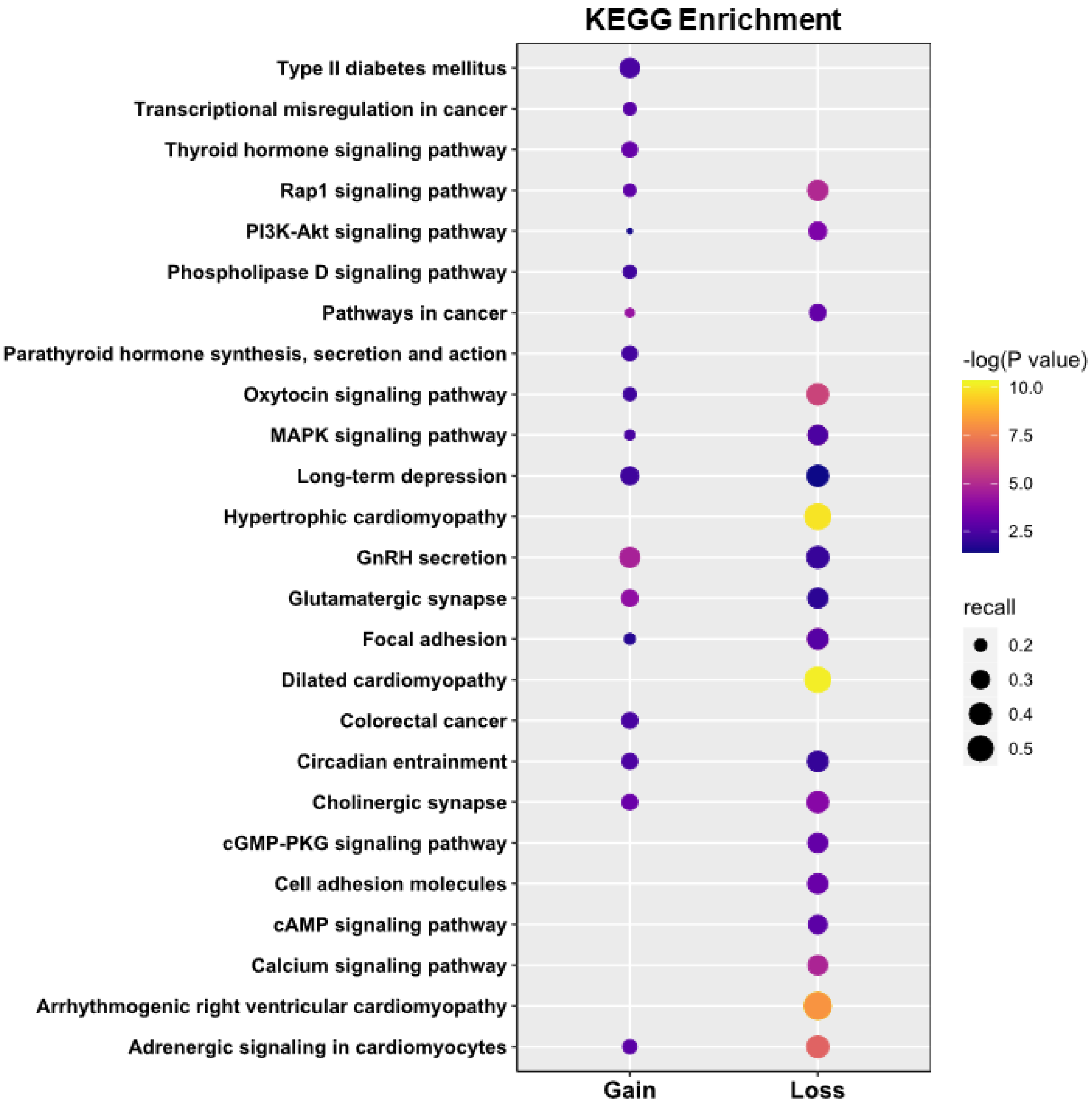

Figure S8. Top 25 enriched KEGG terms for COSMIC and CLINVAR G4 mutations.

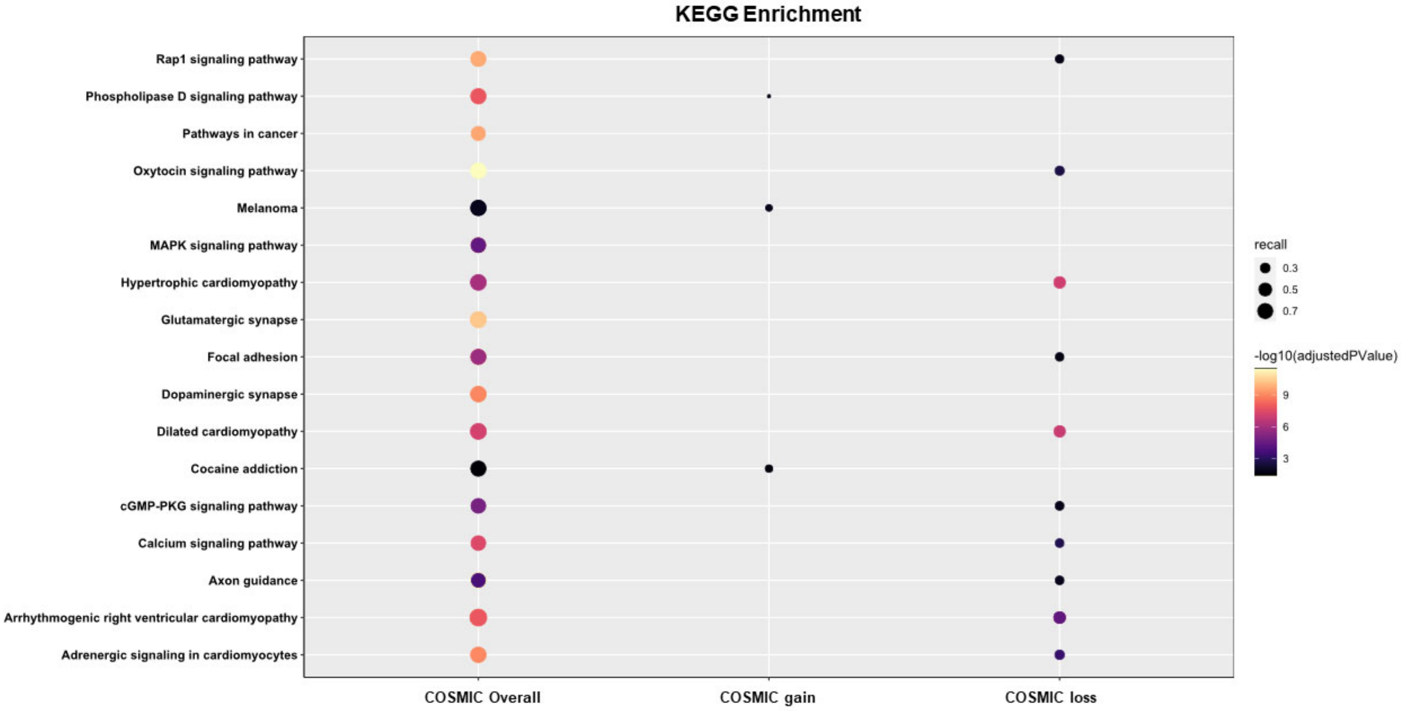

Figure S9. Top 25 enriched KEGG terms for COSMIC G4 mutations.

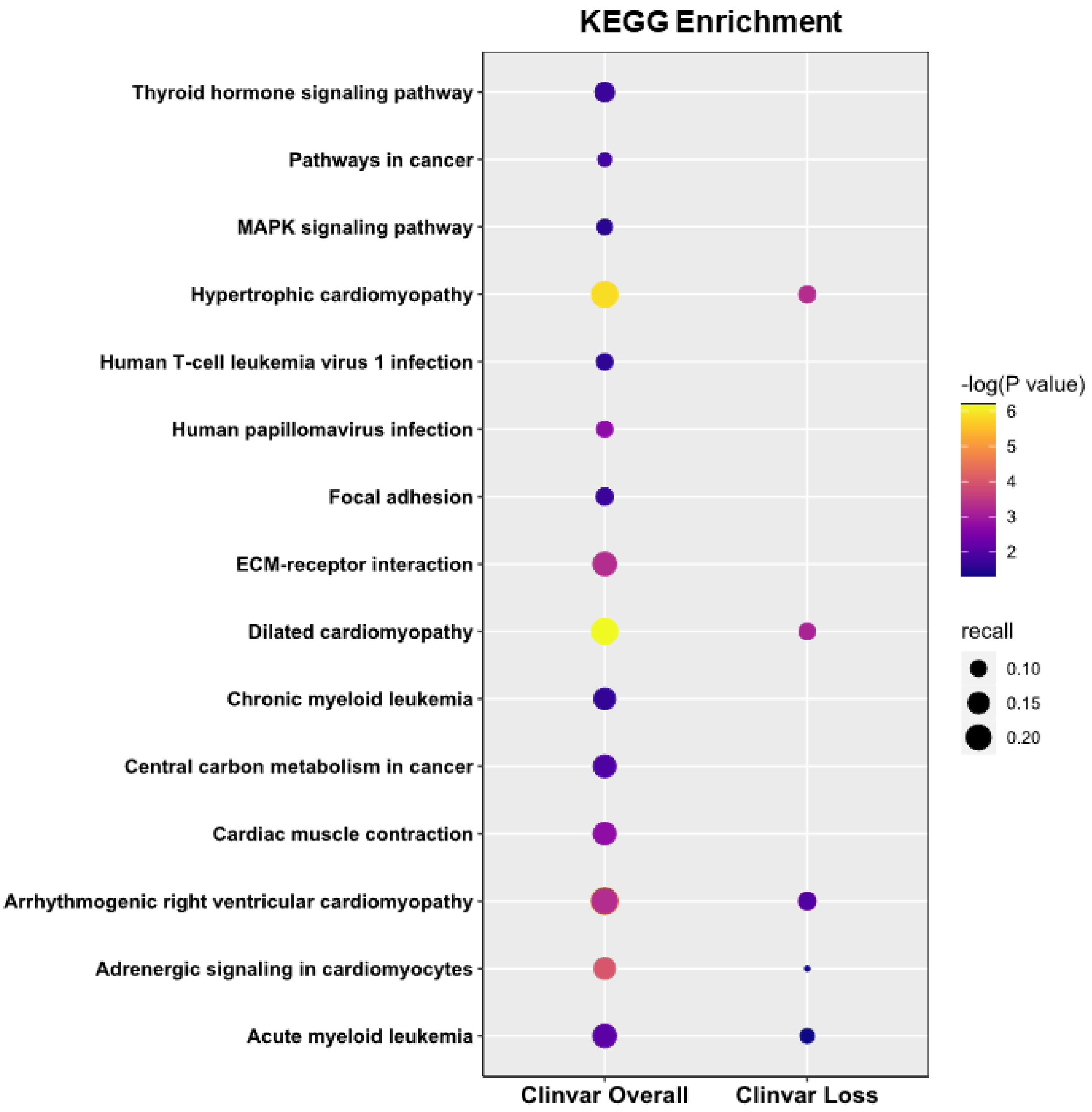

Figure S10. Top 25 enriched KEGG terms for CLINVAR G4 mutations.

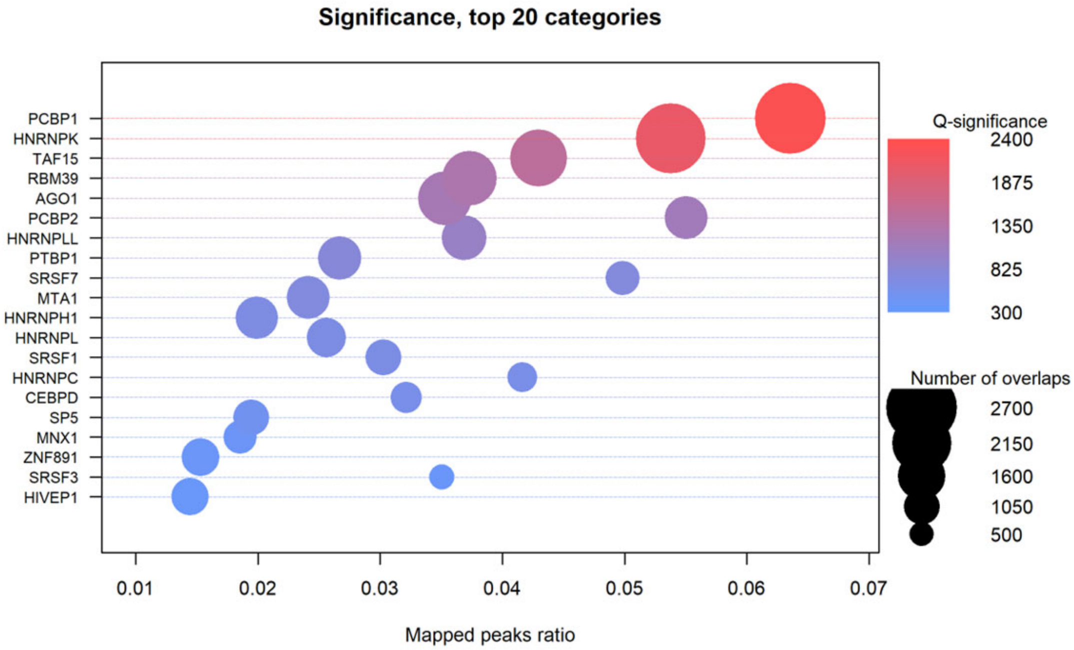

Figure S11. Top 20 enriched transcription factors with overlapping ChIP-seq peaks for COSMIC G4 SNVs in the HEK293 cell line.

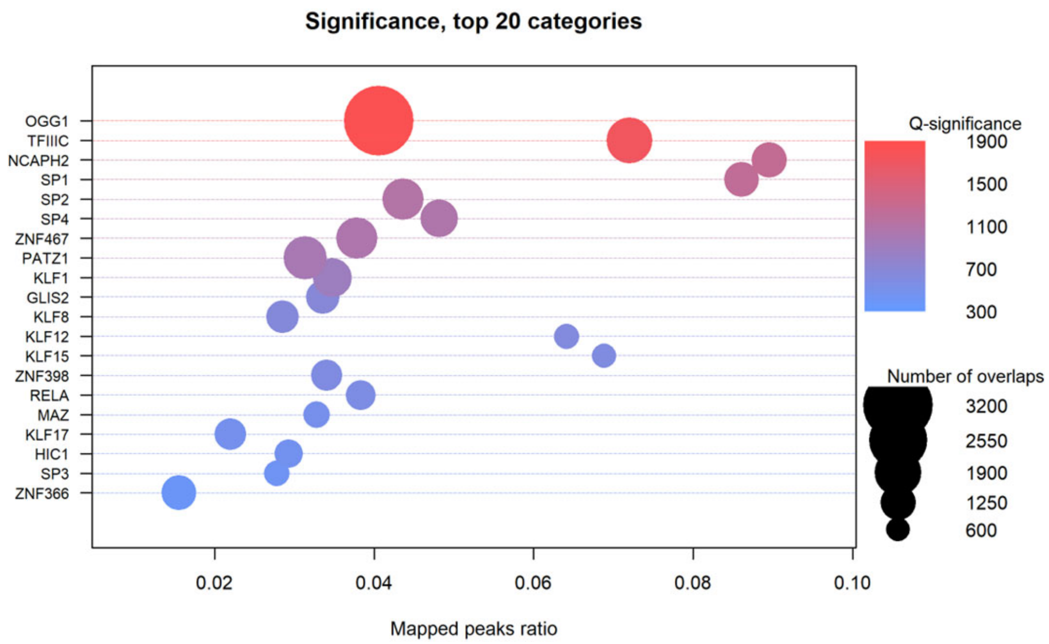

Figure S12. Top 20 enriched transcription factors with overlapping ChIP-seq peaks for COSMIC G4 SNVs in the K562 cell line.

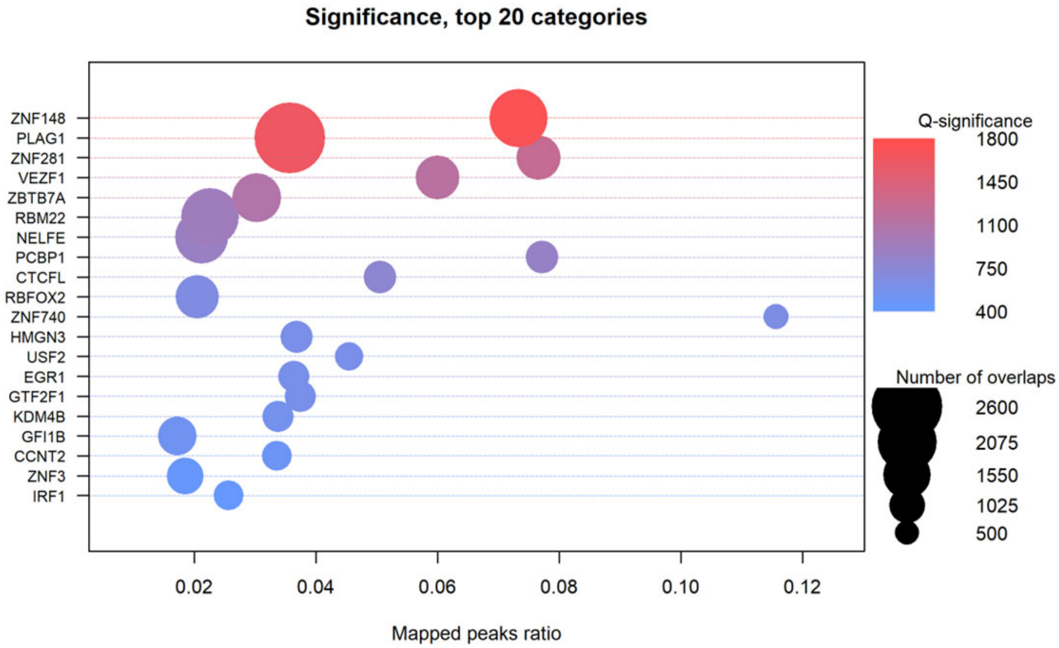

Figure S13. Top 20 enriched transcription factors with overlapping ChIP-seq peaks for COSMIC G4 SNVs in the Hep-G2 cell line.

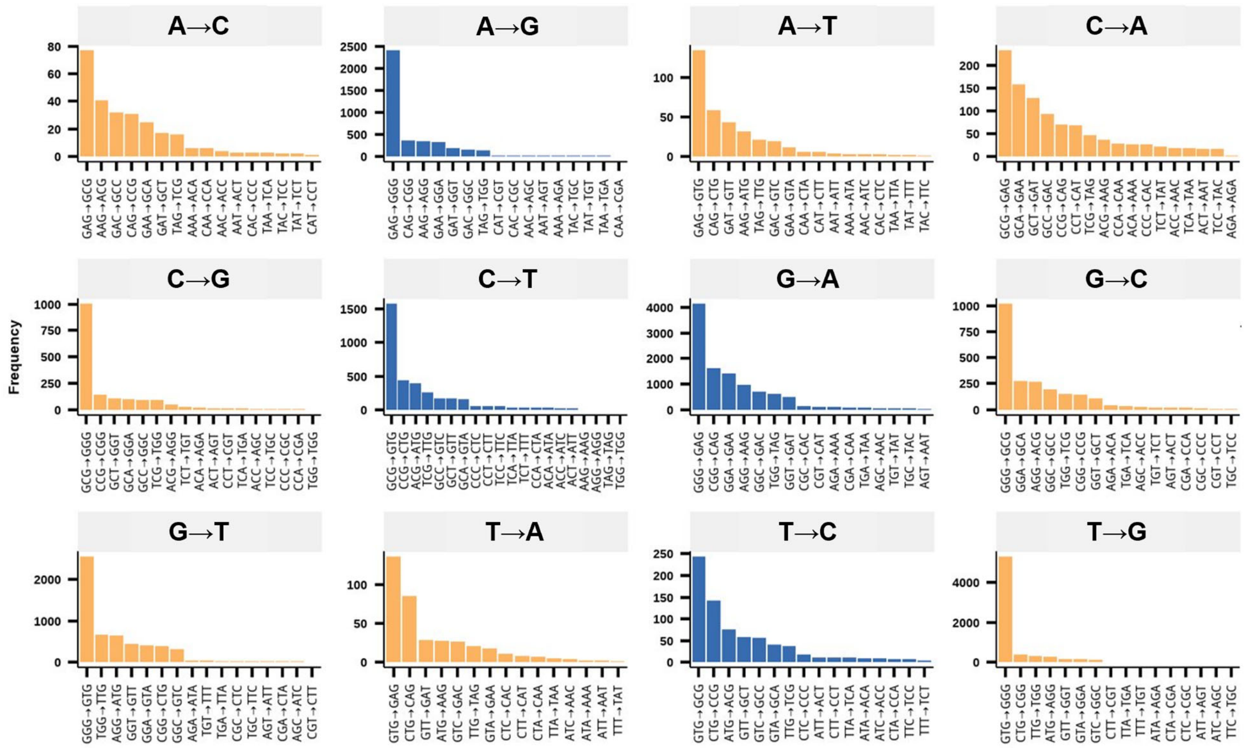

Figure S14. Frequency of SNVs across G-quadruplex regions within trinucleotide contexts for the CLINVAR database.

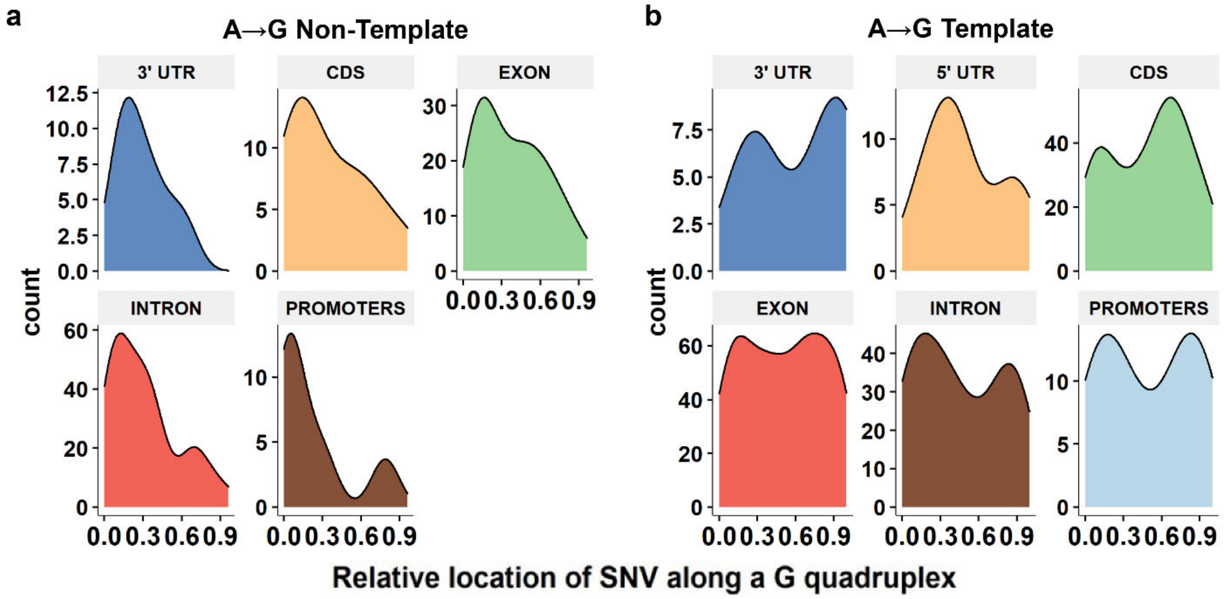

Figure S15. Distribution of A→G SNVs across the G4 region for different features on (a) the non-template and (b) template strand for CLINVAR variants.

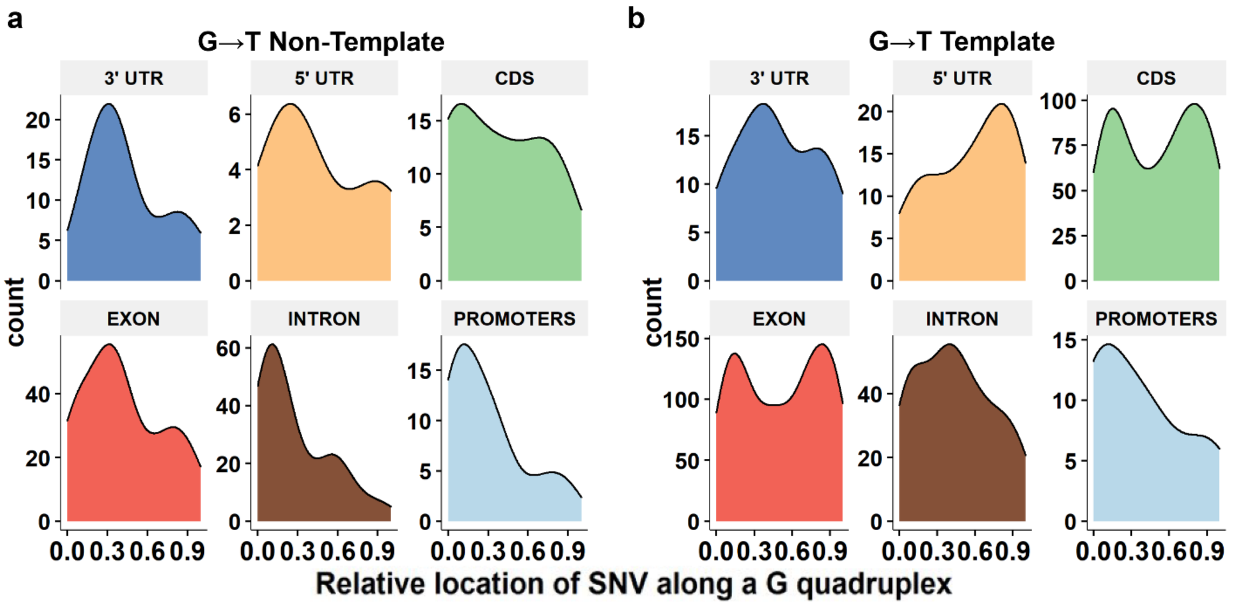

Figure S16. Distribution of G→T SNVs across the G4 region for different features on (a) the non-template and (b) template strand for CLINVAR variants.

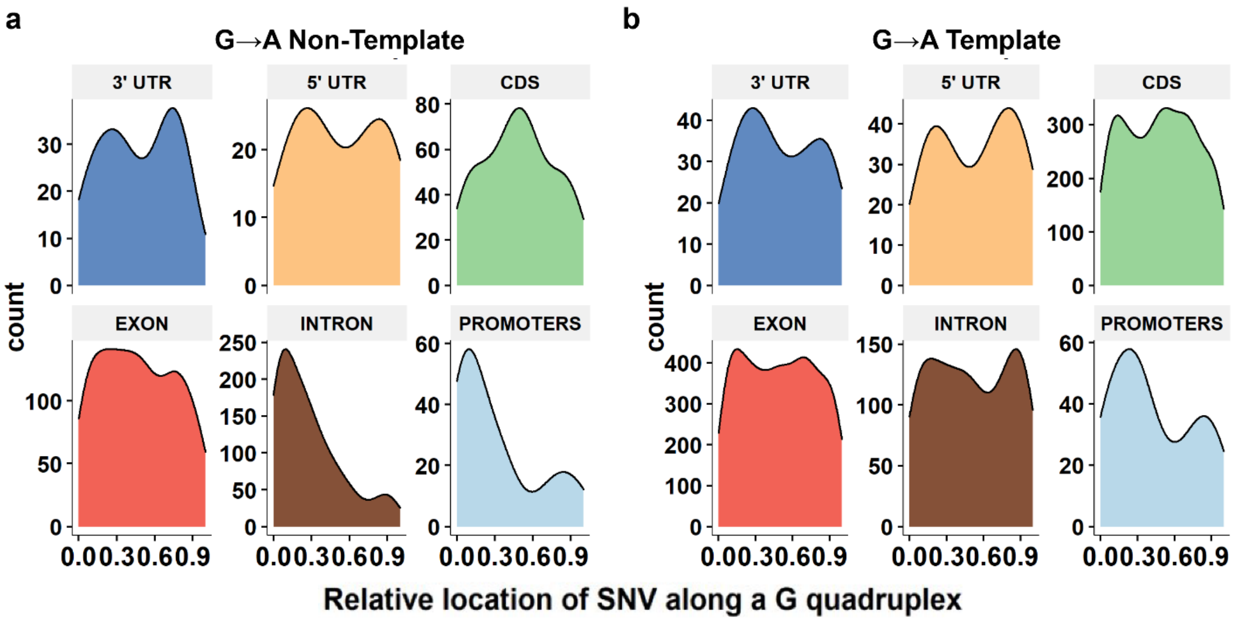

Figure S17. Distribution of G→A SNVs across the G4 region for different features on (a) the non-template and (b) template strand for CLINVAR variants.

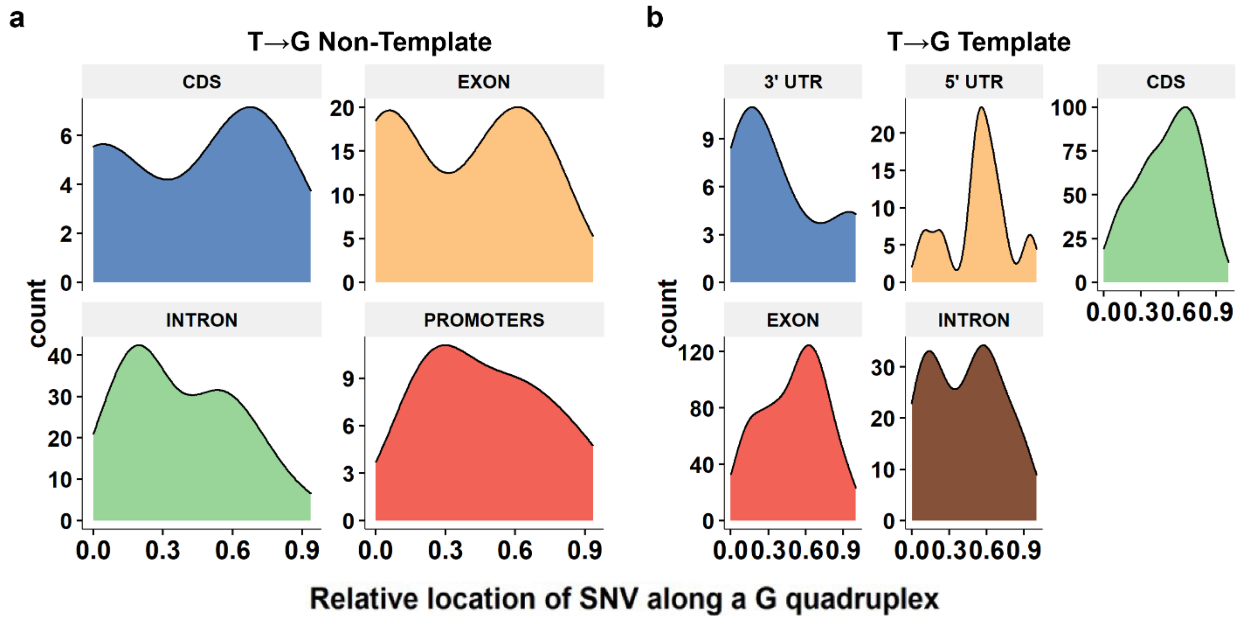

Figure S18. Distribution of T→G SNVs across the G4 region for different features on (a) the non-template and (b) template strand for CLINVAR variants.

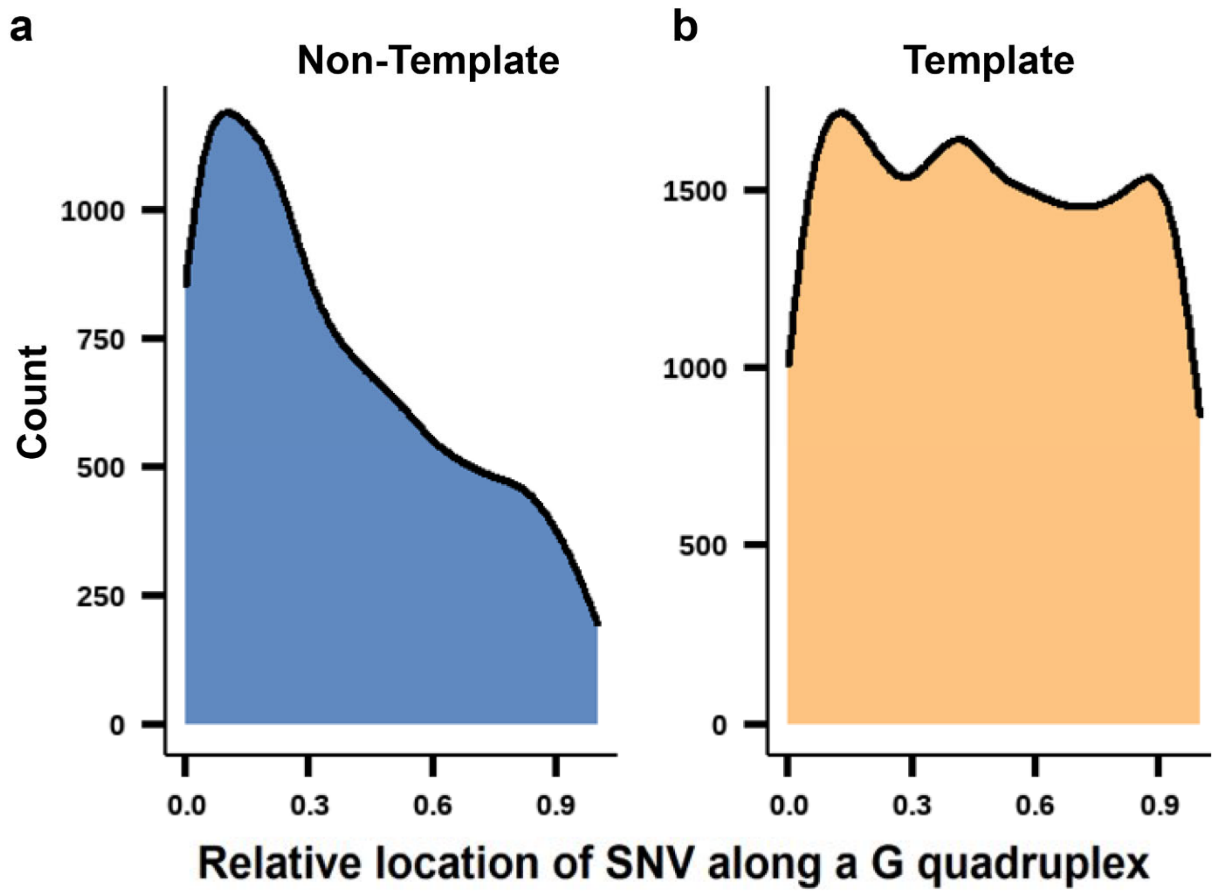

Figure S19. Distribution of SNVs across G-quadruplex regions for the (a) forward and (b) reverse strands for SNVs detected in the CLINVAR database.

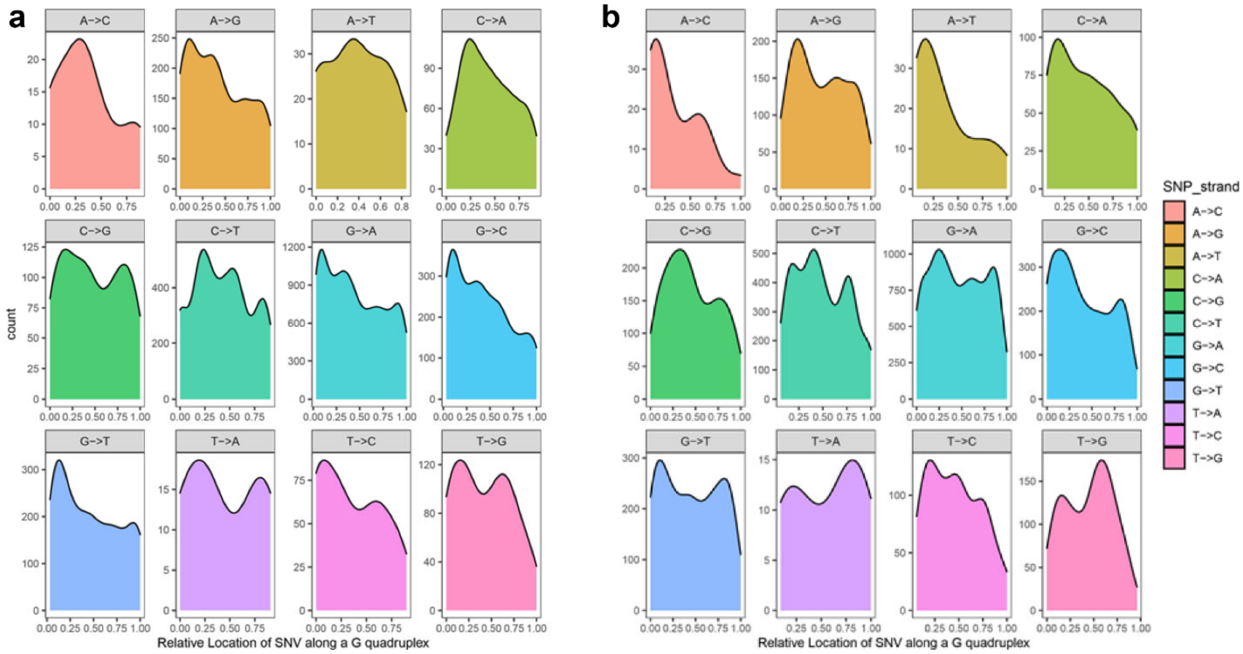

Figure S20. Distribution of SNVs across G-quadruplex regions for the (a) forward and (b) reverse strands for SNVs detected in the CLINVAR database by specific SNV substitution.
