# Supplementary material for: Analysis of Nucleotide Variations in Human G-Quadruplex Forming Regions Associated with Disease States
Source: Genes (Basel). 2023 Nov 25;14(12):2125. doi: 10.3390/genes14122125 (PMC10742762; doi:10.3390/genes14122125)
Supplement: Supplementary file 1 [file genes-14-02125-s001.zip › SupplementalTables.pdf]

Table S1. Count of all observed SNVs in the COSMIC database.

		TO			
		**A**	**C**	**G**	**T**
**FROM**	**A**	0	94,513	2,006,240	1,121,852
	**C**	1,965,512	0	990,818	3,788,310
	**G**	3,788,966	989,193	0	2,017,502
	**T**	1,124,289	2,007,011	951,101	0

Table S2. Counts of SNVs in G4 regions from the COSMIC database.

		TO			
		**A**	**C**	**G**	**T**
**FROM**	**A**	0	601	7,191	768
	**C**	2,529	0	2,937	8,094
	**G**	21,349	4,450	0	10,791
	**T**	742	1,608	8,861	0

Table S3. Changes in putative G4 from the COSMIC database across both strands before and after mutation. (0: absence of pG4; +: presence of pG4 in forward strand; -: presence of pG4 in reverse strand)

G4 with reference allele	G4 with alternate allele	SNV locations	%	G4hunter score (reference)	Sd (reference)	G4hunter score (alternate)	Sd (alternate)
-	-	13,793	36.77	-1.222	0.387	-1.219	0.4
-	0	3,581	9.55	-1.026	0.351	-0.901	0.345
-	+	6	0.02	0.175	0.37	0.279	0.36
0	-	1,354	3.61	-1.082	0.356	-1.196	0.359
0	+	1,374	3.66	1.085	0.368	1.201	0.371
+	0	3,655	9.74	0.993	0.359	0.871	0.355
+	+	13,753	36.66	1.222	0.376	1.219	0.391

Table S4. Count and proportion of variants in experimentally validated G4 regions for different functional regions. Note that annotation regions may overlap, resulting in a single SNV assigned to multiple annotations.

	COSMIC		CLINVAR	
**Annotation**	**Count**	**Frequency**	**Count**	**Frequency**
**CDS**	7,495	32.49	1,031	61.41
**5' UTR**	1,254	5,44	198	11.79
**3' UTR**	2,197	9.52	210	12.51
**EXON**	12,498	34.16	1,552	65.26
**INTRON**	17,264	47.19	804	33.81
**PROMOTER**	1,288	3,52	20	0.84
**ENHANCER**	155	0.42	--	--
**CpG ISLAND**	164	0.45	--	--
**GENCODE lncRNA**	1,492	6.47	--	--
**INTERGENIC**	5,441	23.586	--	--

Table S5. Significant GO:BP enrichments for all COSMIC and CLINVAR G4 mutations.

GO ID	GO Description	Universe	COSMIC and CLINVAR	Adjusted P-value
**GO:0007399**	nervous system development	1648	1087	3.01E-48
**GO:0048856**	anatomical structure development	4152	2427	6.54E-48
**GO:0032502**	developmental process	4584	2644	1.02E-46
**GO:0048731**	system development	2976	1790	1.56E-42
**GO:0007275**	multicellular organism development	3266	1938	2.46E-41
**GO:0009653**	anatomical structure morphogenesis	1871	1181	1.16E-38
**GO:0048699**	generation of neurons	969	670	1.07E-37
**GO:0000902**	cell morphogenesis	718	520	4.07E-37
**GO:0030154**	cell differentiation	2860	1706	5.88E-37
**GO:0048869**	cellular developmental process	2879	1715	1.04E-36
**GO:0022008**	neurogenesis	1096	736	6.89E-35
**GO:0030182**	neuron differentiation	923	636	7.71E-35
**GO:0048468**	cell development	1355	877	4.69E-33
**GO:0032501**	multicellular organismal process	5319	2948	8.54E-33
**GO:0048858**	cell projection morphogenesis	459	352	1.36E-32
**GO:0048666**	neuron development	729	516	2.16E-32
**GO:0032989**	cellular component morphogenesis	549	407	3.74E-32
**GO:0030030**	cell projection organization	1164	766	7.19E-32
**GO:0120039**	plasma membrane bounded cell projection morphogenesis	455	348	9.12E-32
**GO:0031175**	neuron projection development	656	471	9.40E-32
**GO:0120036**	plasma membrane bounded cell projection organization	1144	754	1.22E-31
**GO:0032990**	cell part morphogenesis	469	355	8.10E-31
**GO:0048812**	neuron projection morphogenesis	441	337	1.53E-30
**GO:0000904**	cell morphogenesis involved in differentiation	495	367	1.01E-28
**GO:0023051**	regulation of signaling	2733	1601	2.82E-28
**GO:0010646**	regulation of cell communication	2727	1596	7.06E-28
**GO:0051128**	regulation of cellular component organization	1929	1170	3.23E-27
**GO:0061564**	axon development	323	256	5.08E-27
**GO:0030029**	actin filament-based process	717	495	1.15E-26
**GO:0050793**	regulation of developmental process	1810	1101	5.46E-26
**GO:0048667**	cell morphogenesis involved in neuron differentiation	381	291	6.02E-26
**GO:0007409**	axonogenesis	298	237	3.00E-25
**GO:0023052**	signaling	5190	2837	8.54E-25
**GO:0007154**	cell communication	5221	2844	1.92E-23
**GO:0016043**	cellular component organization	5370	2912	1.84E-22
**GO:0051239**	regulation of multicellular organismal process	2117	1248	3.72E-22
**GO:0048513**	animal organ development	2176	1277	1.06E-21
**GO:0030036**	actin cytoskeleton organization	637	435	1.52E-21
**GO:0035556**	intracellular signal transduction	2168	1267	1.65E-20
**GO:0007155**	cell adhesion	1216	754	1.02E-19
**GO:0009966**	regulation of signal transduction	2464	1416	2.43E-19
**GO:0007010**	cytoskeleton organization	1309	799	2.62E-18
**GO:0031344**	regulation of cell projection organization	467	326	7.18E-18
**GO:0050804**	modulation of chemical synaptic transmission	252	195	8.78E-18
**GO:0120035**	regulation of plasma membrane bounded cell projection organization	453	317	1.42E-17
**GO:0071840**	cellular component organization or biogenesis	5539	2962	1.45E-17
**GO:0099177**	regulation of trans-synaptic signaling	253	195	1.99E-17
**GO:0048522**	positive regulation of cellular process	4704	2544	2.87E-17
**GO:0099536**	synaptic signaling	522	356	5.07E-17
**GO:0048523**	negative regulation of cellular process	3896	2135	6.91E-17
**GO:0034330**	cell junction organization	518	353	8.99E-17
**GO:0010975**	regulation of neuron projection development	288	215	1.91E-16
**GO:0022603**	regulation of anatomical structure morphogenesis	700	456	2.19E-16
**GO:0045595**	regulation of cell differentiation	1129	693	2.71E-16
**GO:0007417**	central nervous system development	584	389	4.80E-16
**GO:0050794**	regulation of cellular process	9523	4886	6.17E-16
**GO:0048518**	positive regulation of biological process	5294	2827	8.88E-16
**GO:0099537**	trans-synaptic signaling	501	340	1.25E-15
**GO:0098916**	anterograde trans-synaptic signaling	495	336	1.93E-15
**GO:0007268**	chemical synaptic transmission	495	336	1.93E-15
**GO:0007165**	signal transduction	4776	2566	2.30E-15
**GO:0009987**	cellular process	14783	7306	2.42E-15
**GO:0048583**	regulation of response to stimulus	3327	1835	3.42E-15
**GO:0097485**	neuron projection guidance	169	137	5.04E-15
**GO:0007411**	axon guidance	169	137	5.04E-15

Table S5 (continued).

GO ID	GO Description	Universe	COSMIC and CLINVAR	Adjusted P-value
**GO:0065007**	biological regulation	10721	5447	5.19E-15
**GO:0048519**	negative regulation of biological process	4381	2365	6.60E-15
**GO:0065008**	regulation of biological quality	2937	1634	6.88E-15
**GO:0032879**	regulation of localization	1615	947	7.50E-15
**GO:0051716**	cellular response to stimulus	5982	3159	9.70E-15
**GO:0050789**	regulation of biological process	10085	5143	1.21E-14
**GO:0007267**	cell-cell signaling	1269	761	1.63E-14
**GO:0051094**	positive regulation of developmental process	967	595	6.68E-14
**GO:0051179**	localization	4343	2335	1.81E-13
**GO:0050770**	regulation of axonogenesis	100	88	2.65E-13
**GO:0051049**	regulation of transport	1327	786	3.15E-13
**GO:0048870**	cell motility	1362	804	4.26E-13
**GO:0007167**	enzyme-linked receptor protein signaling pathway	795	497	5.75E-13
**GO:0009887**	animal organ morphogenesis	582	378	7.07E-13
**GO:0050808**	synapse organization	275	198	3.52E-12
**GO:0051960**	regulation of nervous system development	257	187	3.93E-12
**GO:0016477**	cell migration	1210	717	7.09E-12
**GO:0009888**	tissue development	1239	732	8.83E-12
**GO:0003012**	muscle system process	305	215	8.94E-12
**GO:0055085**	transmembrane transport	1060	635	1.60E-11
**GO:0051130**	positive regulation of cellular component organization	844	518	1.69E-11
**GO:0006810**	transport	3620	1956	1.92E-11
**GO:0065009**	regulation of molecular function	2121	1192	2.01E-11
**GO:0040011**	locomotion	1075	641	4.38E-11
**GO:0098660**	inorganic ion transmembrane transport	622	394	5.04E-11
**GO:0042391**	regulation of membrane potential	319	221	7.21E-11
**GO:0051234**	establishment of localization	3775	2029	7.97E-11
**GO:0007015**	actin filament organization	395	265	8.57E-11
**GO:0045944**	positive regulation of transcription by RNA polymerase II	952	573	1.11E-10
**GO:0007420**	brain development	384	258	1.44E-10
**GO:0098655**	cation transmembrane transport	656	411	1.57E-10
**GO:0023057**	negative regulation of signaling	1067	634	1.61E-10
**GO:0061061**	muscle structure development	407	271	1.72E-10
**GO:0007169**	transmembrane receptor protein tyrosine kinase signaling pathway	511	330	1.77E-10
**GO:0034220**	ion transmembrane transport	816	498	2.25E-10
**GO:0016310**	phosphorylation	1444	833	2.28E-10
**GO:1902531**	regulation of intracellular signal transduction	1429	825	2.39E-10
**GO:0007264**	small GTPase mediated signal transduction	389	260	2.86E-10
**GO:0010648**	negative regulation of cell communication	1060	629	2.89E-10
**GO:0050767**	regulation of neurogenesis	208	153	3.26E-10
**GO:0060322**	head development	402	267	4.05E-10
**GO:0040012**	regulation of locomotion	842	511	4.05E-10
**GO:0006936**	muscle contraction	260	184	4.79E-10
**GO:0022604**	regulation of cell morphogenesis	240	172	4.91E-10
**GO:0098662**	inorganic cation transmembrane transport	572	362	6.98E-10
**GO:0097435**	supramolecular fiber organization	710	438	7.39E-10
**GO:0006811**	ion transport	1187	694	9.42E-10
**GO:0030001**	metal ion transport	678	420	9.61E-10
**GO:0048167**	regulation of synaptic plasticity	122	98	1.02E-09
**GO:2000026**	regulation of multicellular organismal development	981	584	1.04E-09
**GO:0045597**	positive regulation of cell differentiation	624	390	1.13E-09
**GO:2000145**	regulation of cell motility	818	496	1.14E-09
**GO:0030334**	regulation of cell migration	767	468	1.35E-09
**GO:0048585**	negative regulation of response to stimulus	1287	745	2.06E-09
**GO:0072359**	circulatory system development	729	446	2.71E-09
**GO:1903508**	positive regulation of nucleic acid-templated transcription	1297	749	3.44E-09
**GO:0045893**	positive regulation of DNA-templated transcription	1297	749	3.44E-09
**GO:0051173**	positive regulation of nitrogen compound metabolic process	2565	1404	3.55E-09
**GO:0051962**	positive regulation of nervous system development	147	113	3.93E-09
**GO:0031346**	positive regulation of cell projection organization	247	174	3.98E-09
**GO:0006812**	cation transport	886	530	4.38E-09
**GO:0009968**	negative regulation of signal transduction	1009	595	6.51E-09
**GO:0032970**	regulation of actin filament-based process	330	222	6.52E-09

Table S5 (continued).

GO ID	GO Description	Universe	COSMIC and CLINVAR	Adjusted P-value
**GO:0006468**	protein phosphorylation	1268	732	6.97E-09
**GO:0071805**	potassium ion transmembrane transport	185	136	7.67E-09
**GO:1902680**	positive regulation of RNA biosynthetic process	1303	750	8.22E-09
**GO:0031325**	positive regulation of cellular metabolic process	2522	1378	1.21E-08
**GO:0044087**	regulation of cellular component biogenesis	774	467	1.61E-08
**GO:0044057**	regulation of system process	392	256	1.94E-08
**GO:0060560**	developmental growth involved in morphogenesis	135	104	2.36E-08
**GO:0048588**	developmental cell growth	129	100	3.06E-08
**GO:0032535**	regulation of cellular component size	256	177	3.24E-08
**GO:0060284**	regulation of cell development	315	211	4.14E-08
**GO:0051056**	regulation of small GTPase mediated signal transduction	238	166	4.61E-08
**GO:0098609**	cell-cell adhesion	743	448	5.00E-08
**GO:0031589**	cell-substrate adhesion	290	196	6.49E-08
**GO:0001667**	ameboidal-type cell migration	333	220	1.12E-07
**GO:0099587**	inorganic ion import across plasma membrane	112	88	1.40E-07
**GO:0098659**	inorganic cation import across plasma membrane	112	88	1.40E-07
**GO:0009893**	positive regulation of metabolic process	3177	1700	1.57E-07
**GO:0051254**	positive regulation of RNA metabolic process	1433	810	1.66E-07
**GO:0051240**	positive regulation of multicellular organismal process	1137	655	1.76E-07
**GO:0050769**	positive regulation of neurogenesis	119	92	2.53E-07
**GO:0010604**	positive regulation of macromolecule metabolic process	2889	1553	2.57E-07
**GO:0044093**	positive regulation of molecular function	1305	742	2.64E-07
**GO:0031324**	negative regulation of cellular metabolic process	1858	1028	2.90E-07
**GO:0060627**	regulation of vesicle-mediated transport	417	266	2.97E-07
**GO:0045935**	positive regulation of nucleobase-containing compound metabolic process	1620	905	3.26E-07
**GO:0006793**	phosphorus metabolic process	2354	1281	3.36E-07
**GO:0051641**	cellular localization	2721	1467	3.45E-07
**GO:0035725**	sodium ion transmembrane transport	129	98	3.60E-07
**GO:0006813**	potassium ion transport	200	141	4.40E-07
**GO:0033043**	regulation of organelle organization	1015	588	5.17E-07
**GO:1990138**	neuron projection extension	112	87	5.18E-07
**GO:0048638**	regulation of developmental growth	167	121	5.36E-07
**GO:0006796**	phosphate-containing compound metabolic process	2336	1270	5.65E-07
**GO:0042221**	response to chemical	2943	1577	5.88E-07
**GO:0001508**	action potential	117	90	6.38E-07
**GO:0034329**	cell junction assembly	341	222	6.89E-07
**GO:0023056**	positive regulation of signaling	1380	778	7.95E-07
**GO:0032956**	regulation of actin cytoskeleton organization	296	196	9.18E-07
**GO:0030516**	regulation of axon extension	61	53	9.20E-07
**GO:1905114**	cell surface receptor signaling pathway involved in cell-cell signaling	388	248	9.30E-07
**GO:0003015**	heart process	193	136	9.57E-07
**GO:0048646**	anatomical structure formation involved in morphogenesis	751	446	1.03E-06
**GO:0016049**	cell growth	358	231	1.03E-06
**GO:0010647**	positive regulation of cell communication	1374	774	1.07E-06
**GO:0006996**	organelle organization	3147	1677	1.11E-06
**GO:0061387**	regulation of extent of cell growth	67	57	1.13E-06
**GO:0051093**	negative regulation of developmental process	619	374	1.58E-06
**GO:0007507**	heart development	338	219	1.67E-06
**GO:0048589**	developmental growth	273	182	1.68E-06
**GO:0003013**	circulatory system process	443	277	2.44E-06
**GO:0042127**	regulation of cell population proliferation	1218	690	2.85E-06
**GO:0071495**	cellular response to endogenous stimulus	974	562	2.94E-06
**GO:0007265**	Ras protein signal transduction	278	184	3.20E-06
**GO:0060047**	heart contraction	187	131	3.55E-06
**GO:0009719**	response to endogenous stimulus	1076	615	3.74E-06
**GO:0060828**	regulation of canonical Wnt signaling pathway	211	145	3.96E-06
**GO:0051493**	regulation of cytoskeleton organization	450	280	4.00E-06
**GO:0090066**	regulation of anatomical structure size	328	212	4.16E-06
**GO:0031327**	negative regulation of cellular biosynthetic process	1292	727	4.83E-06
**GO:0045892**	negative regulation of DNA-templated transcription	1051	601	5.22E-06
**GO:0003008**	system process	1358	761	5.26E-06
**GO:0006814**	sodium ion transport	166	118	5.64E-06

Table S5 (continued).

GO ID	GO Description	Universe	COSMIC and CLINVAR	Adjusted P-value
**GO:0010557**	positive regulation of macromolecule biosynthetic process	1492	830	5.80E-06
**GO:0009891**	positive regulation of biosynthetic process	1593	882	5.89E-06
**GO:0086001**	cardiac muscle cell action potential	73	60	6.04E-06
**GO:0051172**	negative regulation of nitrogen compound metabolic process	1927	1053	6.26E-06
**GO:1903507**	negative regulation of nucleic acid-templated transcription	1056	603	6.56E-06
**GO:0009890**	negative regulation of biosynthetic process	1315	738	6.62E-06
**GO:1902679**	negative regulation of RNA biosynthetic process	1057	603	7.97E-06
**GO:0098657**	import into cell	216	147	8.46E-06
**GO:0048675**	axon extension	81	65	8.67E-06
**GO:0050772**	positive regulation of axonogenesis	47	42	9.25E-06
**GO:0060537**	muscle tissue development	218	148	9.60E-06
**GO:0050807**	regulation of synapse organization	124	92	9.86E-06
**GO:0008283**	cell population proliferation	1383	772	9.86E-06
**GO:0001558**	regulation of cell growth	320	206	1.11E-05
**GO:0031328**	positive regulation of cellular biosynthetic process	1568	867	1.13E-05
**GO:0009790**	embryo development	505	308	1.26E-05
**GO:0007166**	cell surface receptor signaling pathway	2271	1225	1.38E-05
**GO:0035637**	multicellular organismal signaling	128	94	1.60E-05
**GO:0010631**	epithelial cell migration	261	172	1.68E-05
**GO:0060070**	canonical Wnt signaling pathway	254	168	1.68E-05
**GO:0010632**	regulation of epithelial cell migration	204	139	1.92E-05
**GO:0045596**	negative regulation of cell differentiation	439	271	2.07E-05
**GO:0050896**	response to stimulus	7117	3620	2.09E-05
**GO:0030111**	regulation of Wnt signaling pathway	274	179	2.22E-05
**GO:0010558**	negative regulation of macromolecule biosynthetic process	1251	701	2.32E-05
**GO:0098739**	import across plasma membrane	172	120	2.36E-05
**GO:0034762**	regulation of transmembrane transport	385	241	2.39E-05
**GO:0040007**	growth	515	312	2.61E-05
**GO:0071310**	cellular response to organic substance	1734	949	2.81E-05
**GO:0050803**	regulation of synapse structure or activity	129	94	3.02E-05
**GO:0000165**	MAPK cascade	599	357	3.11E-05
**GO:0014706**	striated muscle tissue development	144	103	3.19E-05
**GO:0035295**	tube development	616	366	3.29E-05
**GO:0090132**	epithelium migration	263	172	3.75E-05
**GO:0050806**	positive regulation of synaptic transmission	89	69	3.90E-05
**GO:0009892**	negative regulation of metabolic process	2418	1295	4.08E-05
**GO:0010033**	response to organic substance	2106	1137	4.17E-05
**GO:0046777**	protein autophosphorylation	199	135	4.63E-05
**GO:0048738**	cardiac muscle tissue development	138	99	4.67E-05
**GO:0001505**	regulation of neurotransmitter levels	143	102	4.68E-05
**GO:0061337**	cardiac conduction	91	70	5.39E-05
**GO:0016358**	dendrite development	155	109	5.39E-05
**GO:0090257**	regulation of muscle system process	157	110	6.38E-05
**GO:0034765**	regulation of ion transmembrane transport	325	206	6.51E-05
**GO:0051253**	negative regulation of RNA metabolic process	1157	649	6.64E-05
**GO:0006941**	striated muscle contraction	142	101	6.81E-05
**GO:0008361**	regulation of cell size	119	87	7.72E-05
**GO:0030155**	regulation of cell adhesion	614	363	7.77E-05
**GO:0035239**	tube morphogenesis	547	327	7.90E-05
**GO:0090130**	tissue migration	267	173	8.81E-05
**GO:0036211**	protein modification process	2994	1581	9.04E-05
**GO:0099003**	vesicle-mediated transport in synapse	121	88	9.62E-05
**GO:0050905**	neuromuscular process	73	58	1.07E-04
**GO:0030048**	actin filament-based movement	113	83	1.10E-04
**GO:0042692**	muscle cell differentiation	243	159	1.16E-04
**GO:0022607**	cellular component assembly	2547	1355	1.30E-04
**GO:0030900**	forebrain development	155	108	1.31E-04
**GO:0060078**	regulation of postsynaptic membrane potential	61	50	1.38E-04
**GO:0007517**	muscle organ development	179	122	1.38E-04
**GO:0009967**	positive regulation of signal transduction	1255	697	1.57E-04
**GO:0048639**	positive regulation of developmental growth	83	64	1.74E-04
**GO:0048729**	tissue morphogenesis	348	217	1.83E-04
**GO:0070887**	cellular response to chemical stimulus	2288	1223	1.83E-04
**GO:0043269**	regulation of ion transport	458	277	1.97E-04

Table S5 (continued).

GO ID	GO Description	Universe	COSMIC and CLINVAR	Adjusted P-value
**GO:0043269**	regulation of ion transport	458	277	1.97E-04
**GO:0035249**	synaptic transmission, glutamatergic	66	53	2.05E-04
**GO:0099504**	synaptic vesicle cycle	114	83	2.07E-04
**GO:0040008**	regulation of growth	416	254	2.14E-04
**GO:0051129**	negative regulation of cellular component organization	566	335	2.15E-04
**GO:0021537**	telencephalon development	101	75	2.19E-04
**GO:0098900**	regulation of action potential	48	41	2.21E-04
**GO:0045934**	negative regulation of nucleobase-containing compound metabolic process	1273	705	2.32E-04
**GO:0099565**	chemical synaptic transmission, postsynaptic	51	43	2.34E-04
**GO:0051241**	negative regulation of multicellular organismal process	755	435	2.53E-04
**GO:0010720**	positive regulation of cell development	177	120	2.71E-04
**GO:0140352**	export from cell	627	367	2.74E-04
**GO:1902532**	negative regulation of intracellular signal transduction	436	264	3.33E-04
**GO:0032409**	regulation of transporter activity	244	158	3.37E-04
**GO:0010605**	negative regulation of macromolecule metabolic process	2244	1198	3.49E-04
**GO:0008016**	regulation of heart contraction	164	112	3.83E-04
**GO:1904062**	regulation of cation transmembrane transport	291	184	4.13E-04
**GO:0070727**	cellular macromolecule localization	1880	1013	4.24E-04
**GO:0010594**	regulation of endothelial cell migration	154	106	4.36E-04
**GO:0043542**	endothelial cell migration	194	129	5.00E-04
**GO:0048598**	embryonic morphogenesis	308	193	5.37E-04
**GO:0008104**	protein localization	1874	1009	5.38E-04
**GO:0044089**	positive regulation of cellular component biogenesis	416	252	5.87E-04
**GO:0050790**	regulation of catalytic activity	1498	817	6.20E-04
**GO:0198738**	cell-cell signaling by wnt	343	212	6.32E-04
**GO:0040017**	positive regulation of locomotion	472	282	6.67E-04
**GO:0060048**	cardiac muscle contraction	111	80	6.71E-04
**GO:0008154**	actin polymerization or depolymerization	155	106	7.08E-04
**GO:1903522**	regulation of blood circulation	188	125	7.51E-04
**GO:0030335**	positive regulation of cell migration	443	266	7.90E-04
**GO:0051247**	positive regulation of protein metabolic process	1264	696	8.30E-04
**GO:0021953**	central nervous system neuron differentiation	74	57	8.76E-04
**GO:0030100**	regulation of endocytosis	159	108	9.23E-04
**GO:0016055**	Wnt signaling pathway	339	209	9.76E-04
**GO:0006836**	neurotransmitter transport	137	95	1.04E-03
**GO:0016192**	vesicle-mediated transport	1326	727	1.05E-03
**GO:2000147**	positive regulation of cell motility	463	276	1.13E-03
**GO:0010243**	response to organonitrogen compound	613	356	1.16E-03
**GO:0007163**	establishment or maintenance of cell polarity	177	118	1.25E-03
**GO:0030178**	negative regulation of Wnt signaling pathway	146	100	1.30E-03
**GO:0099173**	postsynapse organization	99	72	1.41E-03
**GO:0006937**	regulation of muscle contraction	119	84	1.42E-03
**GO:0007229**	integrin-mediated signaling pathway	104	75	1.44E-03
**GO:0070252**	actin-mediated cell contraction	86	64	1.50E-03
**GO:0008360**	regulation of cell shape	116	82	1.72E-03
**GO:0008015**	blood circulation	363	221	1.74E-03
**GO:0060429**	epithelium development	689	395	1.79E-03
**GO:0043087**	regulation of GTPase activity	301	187	1.79E-03
**GO:0032412**	regulation of ion transmembrane transporter activity	222	143	1.84E-03
**GO:0018193**	peptidyl-amino acid modification	1035	575	1.97E-03
**GO:0055001**	muscle cell development	118	83	2.04E-03
**GO:0070848**	response to growth factor	516	303	2.08E-03
**GO:0043085**	positive regulation of catalytic activity	960	536	2.08E-03
**GO:0031623**	receptor internalization	98	71	2.09E-03
**GO:0051050**	positive regulation of transport	709	405	2.15E-03
**GO:0010977**	negative regulation of neuron projection development	85	63	2.27E-03
**GO:0031345**	negative regulation of cell projection organization	125	87	2.31E-03
**GO:1901699**	cellular response to nitrogen compound	468	277	2.40E-03
**GO:0086065**	cell communication involved in cardiac conduction	58	46	2.49E-03
**GO:0031532**	actin cytoskeleton reorganization	105	75	2.56E-03
**GO:0051146**	striated muscle cell differentiation	170	113	2.56E-03
**GO:0051649**	establishment of localization in cell	1698	913	2.68E-03
**GO:0046903**	secretion	638	367	2.72E-03

Table S5 (continued).

GO ID	GO Description	Universe	COSMIC and CLINVAR	Adjusted P-value
**GO:0046578**	regulation of Ras protein signal transduction	139	95	2.74E-03
**GO:0031399**	regulation of protein modification process	1259	689	2.83E-03
**GO:0071417**	cellular response to organonitrogen compound	413	247	2.92E-03
**GO:0048813**	dendrite morphogenesis	97	70	3.06E-03
**GO:0060291**	long-term synaptic potentiation	49	40	3.09E-03
**GO:0071526**	semaphorin-plexin signaling pathway	40	34	3.13E-03
**GO:0050771**	negative regulation of axonogenesis	43	36	3.22E-03
**GO:0022898**	regulation of transmembrane transporter activity	229	146	3.28E-03
**GO:0032940**	secretion by cell	571	331	3.39E-03
**GO:0051174**	regulation of phosphorus metabolic process	1126	620	3.43E-03
**GO:0042325**	regulation of phosphorylation	1004	557	3.54E-03
**GO:0098901**	regulation of cardiac muscle cell action potential	30	27	3.62E-03
**GO:0040013**	negative regulation of locomotion	278	173	3.69E-03
**GO:0007416**	synapse assembly	126	87	3.77E-03
**GO:0019220**	regulation of phosphate metabolic process	1125	619	3.93E-03
**GO:0043408**	regulation of MAPK cascade	534	311	3.94E-03
**GO:0045229**	external encapsulating structure organization	237	150	4.35E-03
**GO:0051966**	regulation of synaptic transmission, glutamatergic	51	41	4.76E-03
**GO:0006898**	receptor-mediated endocytosis	214	137	4.77E-03
**GO:0086003**	cardiac muscle cell contraction	62	48	5.00E-03
**GO:0098703**	calcium ion import across plasma membrane	36	31	5.04E-03
**GO:0086002**	cardiac muscle cell action potential involved in contraction	48	39	5.09E-03
**GO:0033036**	macromolecule localization	2245	1186	5.15E-03
**GO:0001944**	vasculature development	492	288	5.17E-03
**GO:0050890**	cognition	156	104	5.19E-03
**GO:0030198**	extracellular matrix organization	234	148	5.31E-03
**GO:0060079**	excitatory postsynaptic potential	45	37	5.32E-03
**GO:1901888**	regulation of cell junction assembly	144	97	5.54E-03
**GO:0050678**	regulation of epithelial cell proliferation	236	149	5.67E-03
**GO:1902903**	regulation of supramolecular fiber organization	311	190	6.64E-03
**GO:0043062**	extracellular structure organization	235	148	7.38E-03
**GO:0002009**	morphogenesis of an epithelium	275	170	7.74E-03
**GO:0042592**	homeostatic process	1249	680	7.85E-03
**GO:0043412**	macromolecule modification	3198	1659	8.65E-03
**GO:0086091**	regulation of heart rate by cardiac conduction	41	34	9.11E-03
**GO:0110053**	regulation of actin filament organization	216	137	9.48E-03
**GO:0031400**	negative regulation of protein modification process	409	242	1.05E-02
**GO:0001568**	blood vessel development	469	274	1.06E-02
**GO:0090090**	negative regulation of canonical Wnt signaling pathway	118	81	1.13E-02
**GO:0048592**	eye morphogenesis	81	59	1.14E-02
**GO:0051246**	regulation of protein metabolic process	2072	1095	1.17E-02
**GO:0007423**	sensory organ development	271	167	1.19E-02
**GO:2001257**	regulation of cation channel activity	153	101	1.31E-02
**GO:0070588**	calcium ion transmembrane transport	235	147	1.31E-02
**GO:0007611**	learning or memory	115	79	1.37E-02
**GO:0048863**	stem cell differentiation	155	102	1.44E-02
**GO:0031401**	positive regulation of protein modification process	814	454	1.44E-02
**GO:0060341**	regulation of cellular localization	766	429	1.48E-02
**GO:0021954**	central nervous system neuron development	40	33	1.51E-02
**GO:0071363**	cellular response to growth factor stimulus	499	289	1.51E-02
**GO:1901379**	regulation of potassium ion transmembrane transport	70	52	1.53E-02
**GO:0030041**	actin filament polymerization	129	87	1.53E-02
**GO:0048514**	blood vessel morphogenesis	439	257	1.60E-02
**GO:0050773**	regulation of dendrite development	62	47	1.62E-02
**GO:0009792**	embryo development ending in birth or egg hatching	198	126	1.68E-02
**GO:0051961**	negative regulation of nervous system development	85	61	1.69E-02
**GO:0090596**	sensory organ morphogenesis	119	81	1.79E-02
**GO:0001501**	skeletal system development	289	176	1.82E-02
**GO:0120031**	plasma membrane bounded cell projection assembly	483	280	1.85E-02
**GO:0006816**	calcium ion transport	313	189	1.85E-02
**GO:0010634**	positive regulation of epithelial cell migration	133	89	1.92E-02
**GO:0031098**	stress-activated protein kinase signaling cascade	202	128	1.93E-02
**GO:0009628**	response to abiotic stimulus	712	400	1.97E-02
**GO:1990573**	potassium ion import across plasma membrane	48	38	1.99E-02

Table S5 (continued).

GO ID	GO Description	Universe	COSMIC and CLINVAR	Adjusted P-value
**GO:0150104**	transport across blood-brain barrier	87	62	2.03E-02
**GO:0010232**	vascular transport	87	62	2.03E-02
**GO:0007160**	cell-matrix adhesion	195	124	2.04E-02
**GO:0032880**	regulation of protein localization	672	379	2.06E-02
**GO:0043254**	regulation of protein-containing complex assembly	332	199	2.11E-02
**GO:0051258**	protein polymerization	233	145	2.14E-02
**GO:0000122**	negative regulation of transcription by RNA polymerase II	751	420	2.14E-02
**GO:0007612**	learning	53	41	2.48E-02
**GO:0045664**	regulation of neuron differentiation	96	67	2.77E-02
**GO:0010959**	regulation of metal ion transport	298	180	2.80E-02
**GO:0030010**	establishment of cell polarity	108	74	2.86E-02
**GO:0007269**	neurotransmitter secretion	91	64	2.87E-02
**GO:0099643**	signal release from synapse	91	64	2.87E-02
**GO:0051963**	regulation of synapse assembly	58	44	2.89E-02
**GO:0016079**	synaptic vesicle exocytosis	58	44	2.89E-02
**GO:0150063**	visual system development	207	130	3.03E-02
**GO:0051403**	stress-activated MAPK cascade	198	125	3.03E-02
**GO:0061572**	actin filament bundle organization	136	90	3.22E-02
**GO:0048762**	mesenchymal cell differentiation	191	121	3.23E-02
**GO:0007215**	glutamate receptor signaling pathway	44	35	3.51E-02
**GO:0086009**	membrane repolarization	44	35	3.51E-02
**GO:0048880**	sensory system development	213	133	3.64E-02
**GO:0032878**	regulation of establishment or maintenance of cell polarity	26	23	3.65E-02
**GO:0001654**	eye development	204	128	3.68E-02
**GO:0048640**	negative regulation of developmental growth	60	45	3.78E-02
**GO:0030031**	cell projection assembly	498	286	3.80E-02
**GO:0043549**	regulation of kinase activity	599	339	3.81E-02
**GO:0050881**	musculoskeletal movement	41	33	3.82E-02
**GO:0050879**	multicellular organismal movement	41	33	3.82E-02
**GO:1990778**	protein localization to cell periphery	279	169	3.89E-02
**GO:0051017**	actin filament bundle assembly	133	88	3.94E-02
**GO:0086005**	ventricular cardiac muscle cell action potential	29	25	4.10E-02
**GO:0050919**	negative chemotaxis	35	29	4.26E-02
**GO:0030517**	negative regulation of axon extension	32	27	4.29E-02
**GO:0048878**	chemical homeostasis	769	427	4.30E-02
**GO:0042327**	positive regulation of phosphorylation	663	372	4.31E-02
**GO:0050768**	negative regulation of neurogenesis	80	57	4.32E-02
**GO:0031323**	regulation of cellular metabolic process	4966	2520	4.38E-02
**GO:0043009**	chordate embryonic development	183	116	4.45E-02
**GO:0010721**	negative regulation of cell development	109	74	4.53E-02
**GO:0045785**	positive regulation of cell adhesion	369	217	4.75E-02
**GO:0006887**	exocytosis	267	162	4.91E-02
**GO:0045216**	cell-cell junction organization	169	108	4.96E-02
**GO:0001764**	neuron migration	87	61	4.98E-02

Table S6. Significant GO:BP enrichments for all COSMIC G4 mutations.

GO ID	GO Description	Universe	COSMIC	Adjusted
**GO:0007399**	nervous system development	1648	1074	3.73E-48
**GO:0048856**	anatomical structure development	4152	2391	3.71E-47
**GO:0032502**	developmental process	4584	2606	2.53E-46
**GO:0048731**	system development	2976	1762	2.21E-41
**GO:0007275**	multicellular organism development	3266	1908	2.56E-40
**GO:0048699**	generation of neurons	969	663	7.68E-38
**GO:0009653**	anatomical structure morphogenesis	1871	1162	2.2E-37
**GO:0000902**	cell morphogenesis	718	515	2.79E-37
**GO:0030154**	cell differentiation	2860	1682	1.33E-36
**GO:0048869**	cellular developmental process	2879	1691	2.15E-36
**GO:0022008**	neurogenesis	1096	728	4.84E-35
**GO:0030182**	neuron differentiation	923	629	7.45E-35
**GO:0048858**	cell projection morphogenesis	459	349	9.15E-33
**GO:0032989**	cellular component morphogenesis	549	404	1.13E-32
**GO:0048468**	cell development	1355	865	1.38E-32
**GO:0032501**	multicellular organismal process	5319	2904	1.87E-32
**GO:0030030**	cell projection organization	1164	758	3.34E-32
**GO:0048666**	neuron development	729	510	3.54E-32
**GO:0120039**	plasma membrane bounded cell projection morphogenesis	455	345	6.29E-32
**GO:0120036**	plasma membrane bounded cell projection organization	1144	746	6.38E-32
**GO:0031175**	neuron projection development	656	466	1.03E-31
**GO:0032990**	cell part morphogenesis	469	352	4.87E-31
**GO:0048812**	neuron projection morphogenesis	441	334	1.2E-30
**GO:0023051**	regulation of signaling	2733	1582	7.93E-29
**GO:0010646**	regulation of cell communication	2727	1578	1.24E-28
**GO:0000904**	cell morphogenesis involved in differentiation	495	363	1.5E-28
**GO:0051128**	regulation of cellular component organization	1929	1154	5.21E-27
**GO:0061564**	axon development	323	253	1.39E-26
**GO:0030029**	actin filament-based process	717	489	1.87E-26
**GO:0048667**	cell morphogenesis involved in neuron differentiation	381	288	8.11E-26
**GO:0007409**	axonogenesis	298	235	2.6E-25
**GO:0050793**	regulation of developmental process	1810	1084	2.62E-25
**GO:0023052**	signaling	5190	2793	2.45E-24
**GO:0007154**	cell communication	5221	2801	3.47E-23
**GO:0016043**	cellular component organization	5370	2874	3.66E-23
**GO:0030036**	actin cytoskeleton organization	637	432	3E-22
**GO:0051239**	regulation of multicellular organismal process	2117	1227	3.02E-21
**GO:0048513**	animal organ development	2176	1257	4.1E-21
**GO:0007155**	cell adhesion	1216	749	6.57E-21
**GO:0035556**	intracellular signal transduction	2168	1250	1.57E-20
**GO:0009966**	regulation of signal transduction	2464	1398	1.28E-19
**GO:0007010**	cytoskeleton organization	1309	791	7.32E-19
**GO:0050804**	modulation of chemical synaptic transmission	252	194	3.06E-18
**GO:0071840**	cellular component organization or biogenesis	5539	2923	3.12E-18
**GO:0099177**	regulation of trans-synaptic signaling	253	194	6.91E-18
**GO:0048522**	positive regulation of cellular process	4704	2510	9.93E-18
**GO:0031344**	regulation of cell projection organization	467	322	1.09E-17
**GO:0099536**	synaptic signaling	522	353	2.18E-17
**GO:0120035**	regulation of plasma membrane bounded cell projection organization	453	313	2.39E-17
**GO:0034330**	cell junction organization	518	349	9.31E-17
**GO:0048523**	negative regulation of cellular process	3896	2101	2.01E-16
**GO:0048518**	positive regulation of biological process	5294	2788	3.67E-16
**GO:0050794**	regulation of cellular process	9523	4812	4.06E-16
**GO:0045595**	regulation of cell differentiation	1129	683	5.21E-16
**GO:0010975**	regulation of neuron projection development	288	212	5.22E-16
**GO:0099537**	trans-synaptic signaling	501	337	6.23E-16
**GO:0098916**	anterograde trans-synaptic signaling	495	333	9.95E-16
**GO:0007268**	chemical synaptic transmission	495	333	9.95E-16
**GO:0048583**	regulation of response to stimulus	3327	1812	1.08E-15
**GO:0007417**	central nervous system development	584	383	1.53E-15
**GO:0065007**	biological regulation	10721	5366	1.62E-15
**GO:0009987**	cellular process	14783	7189	2.34E-15
**GO:0022603**	regulation of anatomical structure morphogenesis	700	447	2.71E-15
**GO:0007165**	signal transduction	4776	2527	3.25E-15
**GO:0007267**	cell-cell signaling	1269	754	3.55E-15
**GO:0097485**	neuron projection guidance	169	136	3.85E-15

Table S6 (continued).

GO ID	GO Description	Universe	COSMIC	Adjusted
**GO:0007411**	axon guidance	169	136	3.85E-15
**GO:0051716**	cellular response to stimulus	5982	3114	4.88E-15
**GO:0050789**	regulation of biological process	10085	5066	5.3E-15
**GO:0032879**	regulation of localization	1615	935	5.33E-15
**GO:0065008**	regulation of biological quality	2937	1610	8.9E-15
**GO:0048519**	negative regulation of biological process	4381	2326	2.42E-14
**GO:0050770**	regulation of axonogenesis	100	88	7.4E-14
**GO:0051094**	positive regulation of developmental process	967	587	8.44E-14
**GO:0051049**	regulation of transport	1327	777	1.58E-13
**GO:0051179**	localization	4343	2298	3.85E-13
**GO:0050808**	synapse organization	275	197	1.2E-12
**GO:0051960**	regulation of nervous system development	257	186	1.51E-12
**GO:0048870**	cell motility	1362	790	1.94E-12
**GO:0007167**	enzyme-linked receptor protein signaling pathway	795	487	5.4E-12
**GO:0065009**	regulation of molecular function	2121	1178	7.37E-12
**GO:0009887**	animal organ morphogenesis	582	370	7.74E-12
**GO:0098660**	inorganic ion transmembrane transport	622	392	8.1E-12
**GO:0007015**	actin filament organization	395	264	1.69E-11
**GO:0003012**	muscle system process	305	212	1.71E-11
**GO:0051130**	positive regulation of cellular component organization	844	511	2.1E-11
**GO:0040011**	locomotion	1075	634	2.15E-11
**GO:0016477**	cell migration	1210	705	2.25E-11
**GO:0098655**	cation transmembrane transport	656	408	4.28E-11
**GO:0006810**	transport	3620	1924	5E-11
**GO:0055085**	transmembrane transport	1060	624	5.85E-11
**GO:0009888**	tissue development	1239	718	6.11E-11
**GO:0061061**	muscle structure development	407	269	7.1E-11
**GO:0016310**	phosphorylation	1444	824	8.45E-11
**GO:0034220**	ion transmembrane transport	816	493	1.05E-10
**GO:0042391**	regulation of membrane potential	319	218	1.17E-10
**GO:0098662**	inorganic cation transmembrane transport	572	360	1.47E-10
**GO:0050767**	regulation of neurogenesis	208	152	1.8E-10
**GO:1902531**	regulation of intracellular signal transduction	1429	814	2.13E-10
**GO:0045944**	positive regulation of transcription by RNA polymerase II	952	564	2.35E-10
**GO:0051234**	establishment of localization	3775	1995	2.45E-10
**GO:0007420**	brain development	384	254	3.26E-10
**GO:0030001**	metal ion transport	678	416	4.56E-10
**GO:0040012**	regulation of locomotion	842	504	4.98E-10
**GO:0097435**	supramolecular fiber organization	710	433	5.41E-10
**GO:0007264**	small GTPase mediated signal transduction	389	256	6.15E-10
**GO:0007169**	transmembrane receptor protein tyrosine kinase signaling pathway	511	324	6.85E-10
**GO:0060322**	head development	402	263	7.88E-10
**GO:0023057**	negative regulation of signaling	1067	622	8.51E-10
**GO:0010648**	negative regulation of cell communication	1060	618	9.87E-10
**GO:0051962**	positive regulation of nervous system development	147	113	1.03E-09
**GO:0045597**	positive regulation of cell differentiation	624	385	1.21E-09
**GO:0006811**	ion transport	1187	684	1.24E-09
**GO:0048167**	regulation of synaptic plasticity	122	97	1.24E-09
**GO:0006936**	muscle contraction	260	181	1.25E-09
**GO:0051173**	positive regulation of nitrogen compound metabolic process	2565	1387	1.3E-09
**GO:0030334**	regulation of cell migration	767	462	1.33E-09
**GO:0006468**	protein phosphorylation	1268	726	1.34E-09
**GO:2000145**	regulation of cell motility	818	489	1.52E-09
**GO:0022604**	regulation of cell morphogenesis	240	169	1.56E-09
**GO:0071805**	potassium ion transmembrane transport	185	136	1.7E-09
**GO:2000026**	regulation of multicellular organismal development	981	575	1.88E-09
**GO:0006812**	cation transport	886	524	2.72E-09
**GO:1903508**	positive regulation of nucleic acid-templated transcription	1297	739	3.11E-09
**GO:0045893**	positive regulation of DNA-templated transcription	1297	739	3.11E-09
**GO:0031346**	positive regulation of cell projection organization	247	172	4.48E-09
**GO:0048585**	negative regulation of response to stimulus	1287	732	6.54E-09
**GO:0031325**	positive regulation of cellular metabolic process	2522	1360	6.74E-09
**GO:0060560**	developmental growth involved in morphogenesis	135	104	6.79E-09
**GO:1902680**	positive regulation of RNA biosynthetic process	1303	740	7.32E-09
**GO:0048588**	developmental cell growth	129	100	9.07E-09
**GO:0032970**	regulation of actin filament-based process	330	219	9.35E-09

Table S6 (continued).

GO ID	GO Description	Universe	COSMIC	Adjusted
**GO:0009968**	negative regulation of signal transduction	1009	586	1.04E-08
**GO:0098609**	cell-cell adhesion	743	445	1.17E-08
**GO:0032535**	regulation of cellular component size	256	176	1.4E-08
**GO:0044087**	regulation of cellular component biogenesis	774	461	1.54E-08
**GO:0044057**	regulation of system process	392	253	1.88E-08
**GO:0051056**	regulation of small GTPase mediated signal transduction	238	165	2.2E-08
**GO:0098659**	inorganic cation import across plasma membrane	112	88	4.74E-08
**GO:0099587**	inorganic ion import across plasma membrane	112	88	4.74E-08
**GO:0031589**	cell-substrate adhesion	290	194	5.36E-08
**GO:0060284**	regulation of cell development	315	208	6.43E-08
**GO:0072359**	circulatory system development	729	434	7.08E-08
**GO:0050769**	positive regulation of neurogenesis	119	92	8.43E-08
**GO:0006813**	potassium ion transport	200	141	1.05E-07
**GO:0031324**	negative regulation of cellular metabolic process	1858	1016	1.24E-07
**GO:0060627**	regulation of vesicle-mediated transport	417	264	1.34E-07
**GO:0051254**	positive regulation of RNA metabolic process	1433	799	1.48E-07
**GO:0044093**	positive regulation of molecular function	1305	733	1.63E-07
**GO:0051240**	positive regulation of multicellular organismal process	1137	646	1.77E-07
**GO:1990138**	neuron projection extension	112	87	1.81E-07
**GO:0009893**	positive regulation of metabolic process	3177	1674	1.93E-07
**GO:0010604**	positive regulation of macromolecule metabolic process	2889	1531	2.03E-07
**GO:1905114**	cell surface receptor signaling pathway involved in cell-cell signaling	388	247	2.48E-07
**GO:0045935**	positive regulation of nucleobase-containing compound metabolic process	1620	893	2.49E-07
**GO:0042221**	response to chemical	2943	1557	2.51E-07
**GO:0035725**	sodium ion transmembrane transport	129	97	3.85E-07
**GO:0023056**	positive regulation of signaling	1380	769	4.04E-07
**GO:0030516**	regulation of axon extension	61	53	4.35E-07
**GO:0034329**	cell junction assembly	341	220	4.37E-07
**GO:0006793**	phosphorus metabolic process	2354	1261	4.9E-07
**GO:0051641**	cellular localization	2721	1444	5.04E-07
**GO:0033043**	regulation of organelle organization	1015	580	5.1E-07
**GO:0061387**	regulation of extent of cell growth	67	57	5.2E-07
**GO:0010647**	positive regulation of cell communication	1374	765	5.58E-07
**GO:0016049**	cell growth	358	229	6.06E-07
**GO:0032956**	regulation of actin cytoskeleton organization	296	194	7.37E-07
**GO:0006796**	phosphate-containing compound metabolic process	2336	1250	8.5E-07
**GO:0060828**	regulation of canonical Wnt signaling pathway	211	145	9.9E-07
**GO:0045892**	negative regulation of DNA-templated transcription	1051	597	1E-06
**GO:0051093**	negative regulation of developmental process	619	370	1.02E-06
**GO:0048638**	regulation of developmental growth	167	119	1.13E-06
**GO:0006996**	organelle organization	3147	1652	1.13E-06
**GO:0001667**	ameboidal-type cell migration	333	214	1.26E-06
**GO:0048589**	developmental growth	273	180	1.54E-06
**GO:1903507**	negative regulation of nucleic acid-templated transcription	1056	598	1.85E-06
**GO:0031327**	negative regulation of cellular biosynthetic process	1292	719	2.24E-06
**GO:1902679**	negative regulation of RNA biosynthetic process	1057	598	2.26E-06
**GO:0042127**	regulation of cell population proliferation	1218	681	2.26E-06
**GO:0051172**	negative regulation of nitrogen compound metabolic process	1927	1041	2.45E-06
**GO:0090066**	regulation of anatomical structure size	328	210	2.86E-06
**GO:0009890**	negative regulation of biosynthetic process	1315	730	2.92E-06
**GO:0051493**	regulation of cytoskeleton organization	450	277	2.94E-06
**GO:0003015**	heart process	193	133	3.77E-06
**GO:0048675**	axon extension	81	65	3.8E-06
**GO:0060070**	canonical Wnt signaling pathway	254	168	3.9E-06
**GO:0071495**	cellular response to endogenous stimulus	974	553	4.87E-06
**GO:0050772**	positive regulation of axonogenesis	47	42	5.03E-06
**GO:0003013**	circulatory system process	443	272	5.81E-06
**GO:0001508**	action potential	117	87	8.02E-06
**GO:0001558**	regulation of cell growth	320	204	8.08E-06
**GO:0010557**	positive regulation of macromolecule biosynthetic process	1492	817	8.38E-06
**GO:0010558**	negative regulation of macromolecule biosynthetic process	1251	694	8.82E-06
**GO:0009891**	positive regulation of biosynthetic process	1593	868	8.93E-06
**GO:0009719**	response to endogenous stimulus	1076	604	9.26E-06
**GO:0030111**	regulation of Wnt signaling pathway	274	178	1E-05
**GO:0050807**	regulation of synapse organization	124	91	1.07E-05
**GO:0050896**	response to stimulus	7117	3567	1.15E-05

Table S6 (continued).

GO ID	GO Description	Universe	COSMIC	Adjusted
**GO:0007265**	Ras protein signal transduction	278	180	1.19E-05
**GO:0036211**	protein modification process	2994	1566	1.32E-05
**GO:0007166**	cell surface receptor signaling pathway	2271	1207	1.39E-05
**GO:0060047**	heart contraction	187	128	1.41E-05
**GO:0007507**	heart development	338	213	1.5E-05
**GO:0045596**	negative regulation of cell differentiation	439	268	1.62E-05
**GO:0051253**	negative regulation of RNA metabolic process	1157	644	1.66E-05
**GO:0031328**	positive regulation of cellular biosynthetic process	1568	853	1.82E-05
**GO:0008283**	cell population proliferation	1383	759	1.9E-05
**GO:0003008**	system process	1358	746	2.09E-05
**GO:0000165**	MAPK cascade	599	353	2.24E-05
**GO:0048646**	anatomical structure formation involved in morphogenesis	751	433	2.29E-05
**GO:0071310**	cellular response to organic substance	1734	936	2.3E-05
**GO:0034762**	regulation of transmembrane transport	385	238	2.38E-05
**GO:0098657**	import into cell	216	144	2.44E-05
**GO:0040007**	growth	515	308	2.56E-05
**GO:0006814**	sodium ion transport	166	115	2.78E-05
**GO:0008361**	regulation of cell size	119	87	3.03E-05
**GO:0050803**	regulation of synapse structure or activity	129	93	3.1E-05
**GO:0030155**	regulation of cell adhesion	614	360	3.42E-05
**GO:0009790**	embryo development	505	302	3.52E-05
**GO:0098739**	import across plasma membrane	172	118	4.28E-05
**GO:0001505**	regulation of neurotransmitter levels	143	101	4.33E-05
**GO:0010033**	response to organic substance	2106	1120	4.51E-05
**GO:0034765**	regulation of ion transmembrane transport	325	204	4.69E-05
**GO:0009892**	negative regulation of metabolic process	2418	1275	4.96E-05
**GO:0090257**	regulation of muscle system process	157	109	5.34E-05
**GO:0060537**	muscle tissue development	218	144	5.78E-05
**GO:0050806**	positive regulation of synaptic transmission	89	68	5.99E-05
**GO:0046777**	protein autophosphorylation	199	133	6.67E-05
**GO:0060078**	regulation of postsynaptic membrane potential	61	50	7.28E-05
**GO:0009967**	positive regulation of signal transduction	1255	689	8.73E-05
**GO:0045934**	negative regulation of nucleobase-containing compound metabolic process	1273	698	9.18E-05
**GO:0022607**	cellular component assembly	2547	1336	0.000101
**GO:0007517**	muscle organ development	179	121	0.000103
**GO:0099003**	vesicle-mediated transport in synapse	121	87	0.000106
**GO:0035249**	synaptic transmission, glutamatergic	66	53	0.000106
**GO:0070887**	cellular response to chemical stimulus	2288	1207	0.000112
**GO:0016358**	dendrite development	155	107	0.000112
**GO:0030900**	forebrain development	155	107	0.000112
**GO:0035637**	multicellular organismal signaling	128	91	0.000126
**GO:0042692**	muscle cell differentiation	243	157	0.000127
**GO:0010632**	regulation of epithelial cell migration	204	135	0.000129
**GO:0010631**	epithelial cell migration	261	167	0.000131
**GO:0035295**	tube development	616	358	0.000132
**GO:0099565**	chemical synaptic transmission, postsynaptic	51	43	0.000132
**GO:0070727**	cellular macromolecule localization	1880	1002	0.000162
**GO:0140352**	export from cell	627	363	0.000185
**GO:0050905**	neuromuscular process	73	57	0.000203
**GO:0086001**	cardiac muscle cell action potential	73	57	0.000203
**GO:0010720**	positive regulation of cell development	177	119	0.000204
**GO:0008104**	protein localization	1874	998	0.000208
**GO:0050790**	regulation of catalytic activity	1498	809	0.000218
**GO:0099504**	synaptic vesicle cycle	114	82	0.000241
**GO:0043269**	regulation of ion transport	458	273	0.000241
**GO:0198738**	cell-cell signaling by wnt	343	211	0.000251
**GO:0051247**	positive regulation of protein metabolic process	1264	690	0.00026
**GO:0061337**	cardiac conduction	91	68	0.00026
**GO:0090132**	epithelium migration	263	167	0.000269
**GO:0035239**	tube morphogenesis	547	320	0.00027
**GO:0048639**	positive regulation of developmental growth	83	63	0.000281
**GO:0021537**	telencephalon development	101	74	0.000288
**GO:0040008**	regulation of growth	416	250	0.000312
**GO:1904062**	regulation of cation transmembrane transport	291	182	0.000353
**GO:0032409**	regulation of transporter activity	244	156	0.000361
**GO:0010605**	negative regulation of macromolecule metabolic process	2244	1180	0.000368

Table S6 (continued).

GO ID	GO Description	Universe	COSMIC	Adjusted
**GO:0048729**	tissue morphogenesis	348	213	0.000371
**GO:0006941**	striated muscle contraction	142	98	0.000389
**GO:0016055**	Wnt signaling pathway	339	208	0.000396
**GO:1902532**	negative regulation of intracellular signal transduction	436	260	0.000441
**GO:0014706**	striated muscle tissue development	144	99	0.000447
**GO:0040017**	positive regulation of locomotion	472	279	0.000478
**GO:0044089**	positive regulation of cellular component biogenesis	416	249	0.000511
**GO:0031399**	regulation of protein modification process	1259	685	0.000532
**GO:0090130**	tissue migration	267	168	0.000573
**GO:0008154**	actin polymerization or depolymerization	155	105	0.000608
**GO:0030335**	positive regulation of cell migration	443	263	0.000628
**GO:0051129**	negative regulation of cellular component organization	566	328	0.000642
**GO:0098900**	regulation of action potential	48	40	0.000679
**GO:0016192**	vesicle-mediated transport	1326	718	0.000692
**GO:0048738**	cardiac muscle tissue development	138	95	0.000696
**GO:0018193**	peptidyl-amino acid modification	1035	570	0.000741
**GO:0030100**	regulation of endocytosis	159	107	0.000773
**GO:0042325**	regulation of phosphorylation	1004	554	0.000779
**GO:0051174**	regulation of phosphorus metabolic process	1126	616	0.000825
**GO:0051050**	positive regulation of transport	709	402	0.000828
**GO:2000147**	positive regulation of cell motility	463	273	0.000841
**GO:0043085**	positive regulation of catalytic activity	960	531	0.000914
**GO:0030048**	actin filament-based movement	113	80	0.00094
**GO:0019220**	regulation of phosphate metabolic process	1125	615	0.000956
**GO:0031623**	receptor internalization	98	71	0.000992
**GO:0006836**	neurotransmitter transport	137	94	0.000993
**GO:0031345**	negative regulation of cell projection organization	125	87	0.000996
**GO:0010977**	negative regulation of neuron projection development	85	63	0.001143
**GO:0032412**	regulation of ion transmembrane transporter activity	222	142	0.001145
**GO:0030178**	negative regulation of Wnt signaling pathway	146	99	0.001179
**GO:0031532**	actin cytoskeleton reorganization	105	75	0.001196
**GO:0010243**	response to organonitrogen compound	613	351	0.001236
**GO:0048598**	embryonic morphogenesis	308	189	0.001311
**GO:0008016**	regulation of heart contraction	164	109	0.0015
**GO:0006937**	regulation of muscle contraction	119	83	0.001537
**GO:0051241**	negative regulation of multicellular organismal process	755	424	0.001593
**GO:0007416**	synapse assembly	126	87	0.00167
**GO:0007229**	integrin-mediated signaling pathway	104	74	0.001771
**GO:0099173**	postsynapse organization	99	71	0.001812
**GO:0046903**	secretion	638	363	0.001834
**GO:0007163**	establishment or maintenance of cell polarity	177	116	0.001975
**GO:0071526**	semaphorin-plexin signaling pathway	40	34	0.001997
**GO:0050771**	negative regulation of axonogenesis	43	36	0.00202
**GO:0022898**	regulation of transmembrane transporter activity	229	145	0.002021
**GO:0055001**	muscle cell development	118	82	0.002235
**GO:1903522**	regulation of blood circulation	188	122	0.002262
**GO:0043412**	macromolecule modification	3198	1641	0.002376
**GO:0043408**	regulation of MAPK cascade	534	308	0.002429
**GO:0043087**	regulation of GTPase activity	301	184	0.002542
**GO:0031401**	positive regulation of protein modification process	814	453	0.002569
**GO:0046578**	regulation of Ras protein signal transduction	139	94	0.002601
**GO:1901699**	cellular response to nitrogen compound	468	273	0.002746
**GO:0032940**	secretion by cell	571	327	0.002793
**GO:0051966**	regulation of synaptic transmission, glutamatergic	51	41	0.002884
**GO:0008015**	blood circulation	363	217	0.003065
**GO:0051246**	regulation of protein metabolic process	2072	1085	0.0031
**GO:0098703**	calcium ion import across plasma membrane	36	31	0.003323
**GO:0060079**	excitatory postsynaptic potential	45	37	0.003335
**GO:0048813**	dendrite morphogenesis	97	69	0.003994
**GO:0010594**	regulation of endothelial cell migration	154	102	0.004056
**GO:0071417**	cellular response to organonitrogen compound	413	243	0.004126
**GO:0051146**	striated muscle cell differentiation	170	111	0.004196
**GO:0070588**	calcium ion transmembrane transport	235	147	0.004516
**GO:0070848**	response to growth factor	516	297	0.004593
**GO:0008360**	regulation of cell shape	116	80	0.004638
**GO:0021953**	central nervous system neuron differentiation	74	55	0.004743

Table S6 (continued).

GO ID	GO Description	Universe	COSMIC	Adjusted
**GO:0045229**	external encapsulating structure organization	237	148	0.004792
**GO:0051649**	establishment of localization in cell	1698	897	0.004864
**GO:0060048**	cardiac muscle contraction	111	77	0.004996
**GO:1901888**	regulation of cell junction assembly	144	96	0.005129
**GO:0090090**	negative regulation of canonical Wnt signaling pathway	118	81	0.005334
**GO:0033036**	macromolecule localization	2245	1168	0.005414
**GO:0006816**	calcium ion transport	313	189	0.005544
**GO:0060341**	regulation of cellular localization	766	426	0.005581
**GO:0043542**	endothelial cell migration	194	124	0.005618
**GO:0040013**	negative regulation of locomotion	278	170	0.00585
**GO:0006898**	receptor-mediated endocytosis	214	135	0.005907
**GO:0030198**	extracellular matrix organization	234	146	0.005907
**GO:0031400**	negative regulation of protein modification process	409	240	0.006128
**GO:0110053**	regulation of actin filament organization	216	136	0.006298
**GO:0060429**	epithelium development	689	386	0.006326
**GO:0030041**	actin filament polymerization	129	87	0.007112
**GO:0031098**	stress-activated protein kinase signaling cascade	202	128	0.007442
**GO:0060291**	long-term synaptic potentiation	49	39	0.007819
**GO:0043062**	extracellular structure organization	235	146	0.008136
**GO:1901379**	regulation of potassium ion transmembrane transport	70	52	0.00878
**GO:1902903**	regulation of supramolecular fiber organization	311	187	0.008847
**GO:0032880**	regulation of protein localization	672	376	0.009649
**GO:0043549**	regulation of kinase activity	599	338	0.010664
**GO:2001257**	regulation of cation channel activity	153	100	0.011535
**GO:0000122**	negative regulation of transcription by RNA polymerase II	751	416	0.011781
**GO:0002009**	morphogenesis of an epithelium	275	167	0.012154
**GO:0051403**	stress-activated MAPK cascade	198	125	0.012154
**GO:1990573**	potassium ion import across plasma membrane	48	38	0.012766
**GO:0120031**	plasma membrane bounded cell projection assembly	483	277	0.013638
**GO:0051258**	protein polymerization	233	144	0.01366
**GO:0045664**	regulation of neuron differentiation	96	67	0.014735
**GO:0007160**	cell-matrix adhesion	195	123	0.015007
**GO:0061572**	actin filament bundle organization	136	90	0.015215
**GO:0042327**	positive regulation of phosphorylation	663	370	0.015238
**GO:0007612**	learning	53	41	0.015592
**GO:0070252**	actin-mediated cell contraction	86	61	0.016224
**GO:0051338**	regulation of transferase activity	718	398	0.016558
**GO:0098901**	regulation of cardiac muscle cell action potential	30	26	0.016851
**GO:0051963**	regulation of synapse assembly	58	44	0.017849
**GO:0086065**	cell communication involved in cardiac conduction	58	44	0.017849
**GO:0051017**	actin filament bundle assembly	133	88	0.018951
**GO:0050678**	regulation of epithelial cell proliferation	236	145	0.019404
**GO:1990778**	protein localization to cell periphery	279	168	0.021945
**GO:0042592**	homeostatic process	1249	666	0.022003
**GO:0009792**	embryo development ending in birth or egg hatching	198	124	0.022257
**GO:0071363**	cellular response to growth factor stimulus	499	284	0.022775
**GO:0007215**	glutamate receptor signaling pathway	44	35	0.023272
**GO:0048640**	negative regulation of developmental growth	60	45	0.023304
**GO:0051961**	negative regulation of nervous system development	85	60	0.023796
**GO:0010959**	regulation of metal ion transport	298	178	0.023909
**GO:0097553**	calcium ion transmembrane import into cytosol	146	95	0.024141
**GO:0048762**	mesenchymal cell differentiation	191	120	0.024283
**GO:0048863**	stem cell differentiation	155	100	0.02487
**GO:0050879**	multicellular organismal movement	41	33	0.025765
**GO:0086091**	regulation of heart rate by cardiac conduction	41	33	0.025765
**GO:0050881**	musculoskeletal movement	41	33	0.025765
**GO:0032878**	regulation of establishment or maintenance of cell polarity	26	23	0.026744
**GO:0030031**	cell projection assembly	498	283	0.027121
**GO:0010232**	vascular transport	87	61	0.027857
**GO:0150104**	transport across blood-brain barrier	87	61	0.027857
**GO:0050919**	negative chemotaxis	35	29	0.029641
**GO:0050773**	regulation of dendrite development	62	46	0.029645
**GO:0030517**	negative regulation of axon extension	32	27	0.030265
**GO:0042982**	amyloid precursor protein metabolic process	67	49	0.030628
**GO:0007423**	sensory organ development	271	163	0.030834
**GO:0045785**	positive regulation of cell adhesion	369	215	0.032573

Table S6 (continued).

GO ID	GO Description	Universe	COSMIC	Adjusted
**GO:0009611**	response to wounding	392	227	0.034051
**GO:0050890**	cognition	156	100	0.035799
**GO:0001932**	regulation of protein phosphorylation	882	479	0.035846
**GO:0031323**	regulation of cellular metabolic process	4966	2482	0.036928
**GO:0051899**	membrane depolarization	64	47	0.037241
**GO:0001944**	vasculature development	492	279	0.037414
**GO:0002090**	regulation of receptor internalization	43	34	0.037523
**GO:0099643**	signal release from synapse	91	63	0.037593
**GO:0007269**	neurotransmitter secretion	91	63	0.037593
**GO:0010634**	positive regulation of epithelial cell migration	133	87	0.038739
**GO:0043254**	regulation of protein-containing complex assembly	332	195	0.039459
**GO:0045773**	positive regulation of axon extension	22	20	0.039621
**GO:0018108**	peptidyl-tyrosine phosphorylation	246	149	0.040743
**GO:0090596**	sensory organ morphogenesis	119	79	0.040966
**GO:0021954**	central nervous system neuron development	40	32	0.042039
**GO:2000249**	regulation of actin cytoskeleton reorganization	40	32	0.042039
**GO:0001933**	negative regulation of protein phosphorylation	261	157	0.042119
**GO:0018212**	peptidyl-tyrosine modification	248	150	0.042279
**GO:0010562**	positive regulation of phosphorus metabolic process	722	397	0.042279
**GO:0045937**	positive regulation of phosphate metabolic process	722	397	0.042279
**GO:0051668**	localization within membrane	570	319	0.042433
**GO:0048592**	eye morphogenesis	81	57	0.042492
**GO:0033554**	cellular response to stress	1640	858	0.043396
**GO:0045055**	regulated exocytosis	162	103	0.044095
**GO:1901021**	positive regulation of calcium ion transmembrane transporter activity	37	30	0.046133
**GO:0051347**	positive regulation of transferase activity	447	255	0.046166
**GO:0044092**	negative regulation of molecular function	758	415	0.046963
**GO:0006935**	chemotaxis	480	272	0.049239
**GO:0042330**	taxis	480	272	0.049239

Table S7. Significant GO:BP enrichments for all CLINVAR G4 mutations.

GO ID	GO Description	Universe	CLINVAR	Adjusted P-value
**GO:0006941**	striated muscle contraction	142	32	1.1E-11
**GO:0060048**	cardiac muscle contraction	111	27	2.1E-10
**GO:0050905**	neuromuscular process	73	22	4.5E-10
**GO:0035637**	multicellular organismal signaling	128	28	1.1E-09
**GO:0001508**	action potential	117	26	5.2E-09
**GO:0030048**	actin filament-based movement	113	25	1.5E-08
**GO:0070252**	actin-mediated cell contraction	86	20	7.0E-07
**GO:0061337**	cardiac conduction	91	20	1.9E-06
**GO:0086001**	cardiac muscle cell action potential	73	18	2.0E-06
**GO:0006937**	regulation of muscle contraction	119	22	7.3E-06
**GO:0086003**	cardiac muscle cell contraction	62	15	5.8E-05
**GO:0051899**	membrane depolarization	64	15	8.9E-05
**GO:1903115**	regulation of actin filament-based movement	32	11	9.0E-05
**GO:0060415**	muscle tissue morphogenesis	47	13	9.1E-05
**GO:0048644**	muscle organ morphogenesis	47	13	9.1E-05
**GO:0002027**	regulation of heart rate	84	17	1.1E-04
**GO:0098900**	regulation of action potential	48	13	1.2E-04
**GO:0086002**	cardiac muscle cell action potential involved in contraction	48	13	1.2E-04
**GO:0050954**	sensory perception of mechanical stimulus	106	18	5.7E-04
**GO:0086065**	cell communication involved in cardiac conduction	58	13	1.0E-03
**GO:0006942**	regulation of striated muscle contraction	78	15	1.1E-03
**GO:0007605**	sensory perception of sound	100	17	1.1E-03
**GO:0086091**	regulation of heart rate by cardiac conduction	41	11	1.2E-03
**GO:0050881**	musculoskeletal movement	41	11	1.2E-03
**GO:0050879**	multicellular organismal movement	41	11	1.2E-03
**GO:0019226**	transmission of nerve impulse	42	11	1.5E-03
**GO:0055008**	cardiac muscle tissue morphogenesis	42	11	1.5E-03
**GO:0048738**	cardiac muscle tissue development	138	20	1.5E-03
**GO:0055001**	muscle cell development	118	18	2.5E-03
**GO:0003009**	skeletal muscle contraction	36	10	2.5E-03
**GO:0014706**	striated muscle tissue development	144	20	2.8E-03
**GO:0086005**	ventricular cardiac muscle cell action potential	29	9	3.1E-03
**GO:0050885**	neuromuscular process controlling balance	16	7	3.4E-03
**GO:0098901**	regulation of cardiac muscle cell action potential	30	9	4.2E-03
**GO:0003229**	ventricular cardiac muscle tissue development	39	10	5.3E-03
**GO:0048592**	eye morphogenesis	81	14	8.1E-03
**GO:0055010**	ventricular cardiac muscle tissue morphogenesis	33	9	9.3E-03
**GO:0055117**	regulation of cardiac muscle contraction	61	12	9.8E-03
**GO:0086019**	cell-cell signaling involved in cardiac conduction	34	9	1.2E-02
**GO:0008306**	associative learning	19	7	1.2E-02
**GO:0086010**	membrane depolarization during action potential	35	9	1.5E-02
**GO:0050953**	sensory perception of light stimulus	138	18	1.9E-02
**GO:0086004**	regulation of cardiac muscle cell contraction	28	8	2.0E-02
**GO:0043589**	skin morphogenesis	5	4	2.6E-02
**GO:0002028**	regulation of sodium ion transport	68	12	2.7E-02
**GO:0048661**	positive regulation of smooth muscle cell proliferation	49	10	3.6E-02
**GO:0048483**	autonomic nervous system development	31	8	4.1E-02
**GO:0060348**	bone development	108	15	4.5E-02

Table S8. Significant GO:BP enrichments for COSMIC and CLINVAR G4 mutations leading to the loss of a G4.

GO ID	GO Description	Universe	COSMIC and CLINVAR	Adjusted P-value
**GO:0032502**	developmental process	4584	1138	3.23E-24
**GO:0048856**	anatomical structure development	4152	1043	2.82E-23
**GO:0007399**	nervous system development	1648	488	1.15E-22
**GO:0009653**	anatomical structure morphogenesis	1871	539	2.18E-22
**GO:0048731**	system development	2976	784	9.30E-22
**GO:0007275**	multicellular organism development	3266	842	1.00E-20
**GO:0032501**	multicellular organismal process	5319	1270	1.40E-20
**GO:0030154**	cell differentiation	2860	742	3.10E-18
**GO:0048699**	generation of neurons	969	309	3.31E-18
**GO:0048869**	cellular developmental process	2879	745	5.24E-18
**GO:0016043**	cellular component organization	5370	1259	6.78E-17
**GO:0022008**	neurogenesis	1096	335	1.06E-16
**GO:0030182**	neuron differentiation	923	292	1.76E-16
**GO:0071840**	cellular component organization or biogenesis	5539	1277	2.38E-14
**GO:0023051**	regulation of signaling	2733	694	3.06E-14
**GO:0010646**	regulation of cell communication	2727	692	4.16E-14
**GO:0048666**	neuron development	729	236	4.22E-14
**GO:0000904**	cell morphogenesis involved in differentiation	495	175	7.97E-14
**GO:0048468**	cell development	1355	385	9.16E-14
**GO:0048513**	animal organ development	2176	567	3.84E-13
**GO:0023052**	signaling	5190	1196	9.50E-13
**GO:0032989**	cellular component morphogenesis	549	186	1.02E-12
**GO:0007154**	cell communication	5221	1200	2.02E-12
**GO:0031175**	neuron projection development	656	212	2.53E-12
**GO:0048667**	cell morphogenesis involved in neuron differentiation	381	140	3.85E-12
**GO:0034330**	cell junction organization	518	176	4.81E-12
**GO:0000902**	cell morphogenesis	718	226	6.91E-12
**GO:0048812**	neuron projection morphogenesis	441	155	9.70E-12
**GO:0007010**	cytoskeleton organization	1309	365	1.42E-11
**GO:0009966**	regulation of signal transduction	2464	621	1.59E-11
**GO:0009887**	animal organ morphogenesis	582	190	2.87E-11
**GO:0007155**	cell adhesion	1216	342	3.26E-11
**GO:0120036**	plasma membrane bounded cell projection organization	1144	325	3.84E-11
**GO:0120039**	plasma membrane bounded cell projection morphogenesis	455	157	3.85E-11
**GO:0048858**	cell projection morphogenesis	459	157	8.90E-11
**GO:0030030**	cell projection organization	1164	328	9.33E-11
**GO:0032990**	cell part morphogenesis	469	159	1.42E-10
**GO:0030029**	actin filament-based process	717	219	5.92E-10
**GO:0061564**	axon development	323	118	1.06E-09
**GO:0007409**	axonogenesis	298	111	1.25E-09
**GO:0050808**	synapse organization	275	104	2.31E-09
**GO:0050793**	regulation of developmental process	1810	465	4.44E-09
**GO:0051128**	regulation of cellular component organization	1929	490	6.77E-09
**GO:0007165**	signal transduction	4776	1078	4.27E-08
**GO:0016477**	cell migration	1210	325	7.79E-08
**GO:0048870**	cell motility	1362	359	8.30E-08
**GO:0098609**	cell-cell adhesion	743	217	8.39E-08
**GO:0051716**	cellular response to stimulus	5982	1316	9.92E-08
**GO:0035556**	intracellular signal transduction	2168	534	1.26E-07
**GO:0048583**	regulation of response to stimulus	3327	777	1.74E-07
**GO:0051239**	regulation of multicellular organismal process	2117	522	1.84E-07
**GO:0099537**	trans-synaptic signaling	501	157	2.02E-07
**GO:0099536**	synaptic signaling	522	162	2.28E-07
**GO:0009888**	tissue development	1239	329	2.43E-07
**GO:0098916**	anterograde trans-synaptic signaling	495	155	2.81E-07
**GO:0007268**	chemical synaptic transmission	495	155	2.81E-07
**GO:0065007**	biological regulation	10721	2221	3.01E-07
**GO:0072359**	circulatory system development	729	211	3.69E-07
**GO:0065008**	regulation of biological quality	2937	692	5.65E-07
**GO:0050794**	regulation of cellular process	9523	1993	7.40E-07
**GO:0007267**	cell-cell signaling	1269	333	7.84E-07
**GO:0003008**	system process	1358	351	1.64E-06
**GO:0007417**	central nervous system development	584	173	2.68E-06
**GO:0009987**	cellular process	14783	2936	4.03E-06

Table S8 (continued).

GO ID	GO Description	Universe	COSMIC and CLINVAR	Adjusted P-value
**GO:0040011**	locomotion	1075	285	5.65E-06
**GO:0050789**	regulation of biological process	10085	2089	8.77E-06
**GO:1905114**	cell surface receptor signaling pathway involved in cell-cell signaling	388	123	1.03E-05
**GO:0030036**	actin cytoskeleton organization	637	183	1.15E-05
**GO:0050804**	modulation of chemical synaptic transmission	252	88	1.18E-05
**GO:0030048**	actin filament-based movement	113	49	1.44E-05
**GO:0099177**	regulation of trans-synaptic signaling	253	88	1.46E-05
**GO:0006812**	cation transport	886	240	1.55E-05
**GO:0030001**	metal ion transport	678	192	1.58E-05
**GO:0010975**	regulation of neuron projection development	288	97	1.61E-05
**GO:0060047**	heart contraction	187	70	1.80E-05
**GO:0006811**	ion transport	1187	307	1.99E-05
**GO:0048518**	positive regulation of biological process	5294	1158	2.05E-05
**GO:0034329**	cell junction assembly	341	110	2.35E-05
**GO:0032879**	regulation of localization	1615	400	2.54E-05
**GO:0044057**	regulation of system process	392	122	3.78E-05
**GO:0007411**	axon guidance	169	64	4.59E-05
**GO:0097485**	neuron projection guidance	169	64	4.59E-05
**GO:0051179**	localization	4343	964	4.59E-05
**GO:0048522**	positive regulation of cellular process	4704	1036	5.30E-05
**GO:0007507**	heart development	338	108	5.46E-05
**GO:0006936**	muscle contraction	260	88	6.00E-05
**GO:0055085**	transmembrane transport	1060	276	6.09E-05
**GO:0034220**	ion transmembrane transport	816	221	6.22E-05
**GO:0042391**	regulation of membrane potential	319	103	6.34E-05
**GO:0048523**	negative regulation of cellular process	3896	872	6.65E-05
**GO:0003015**	heart process	193	70	7.53E-05
**GO:0007166**	cell surface receptor signaling pathway	2271	536	8.29E-05
**GO:0003012**	muscle system process	305	99	8.73E-05
**GO:1902531**	regulation of intracellular signal transduction	1429	356	9.28E-05
**GO:0061061**	muscle structure development	407	124	1.07E-04
**GO:0040012**	regulation of locomotion	842	225	1.46E-04
**GO:0098655**	cation transmembrane transport	656	182	1.81E-04
**GO:0048646**	anatomical structure formation involved in morphogenesis	751	203	2.65E-04
**GO:0048519**	negative regulation of biological process	4381	964	2.73E-04
**GO:0048729**	tissue morphogenesis	348	108	2.78E-04
**GO:0007416**	synapse assembly	126	50	2.85E-04
**GO:0022603**	regulation of anatomical structure morphogenesis	700	191	3.10E-04
**GO:0051960**	regulation of nervous system development	257	85	3.10E-04
**GO:0030334**	regulation of cell migration	767	206	3.51E-04
**GO:0050905**	neuromuscular process	73	34	3.51E-04
**GO:0031589**	cell-substrate adhesion	290	93	3.99E-04
**GO:0050896**	response to stimulus	7117	1503	4.02E-04
**GO:0031344**	regulation of cell projection organization	467	136	4.37E-04
**GO:0003013**	circulatory system process	443	130	5.12E-04
**GO:0065009**	regulation of molecular function	2121	498	5.42E-04
**GO:0006810**	transport	3620	807	5.65E-04
**GO:0120035**	regulation of plasma membrane bounded cell projection organization	453	132	6.34E-04
**GO:0008015**	blood circulation	363	110	7.79E-04
**GO:0030111**	regulation of Wnt signaling pathway	274	88	7.79E-04
**GO:0007420**	brain development	384	115	8.24E-04
**GO:0051130**	positive regulation of cellular component organization	844	221	9.67E-04
**GO:2000145**	regulation of cell motility	818	215	1.04E-03
**GO:0006996**	organelle organization	3147	708	1.04E-03
**GO:0070252**	actin-mediated cell contraction	86	37	1.15E-03
**GO:0051056**	regulation of small GTPase mediated signal transduction	238	78	1.44E-03
**GO:0050877**	nervous system process	755	200	1.46E-03
**GO:0051234**	establishment of localization	3775	834	1.55E-03
**GO:0060048**	cardiac muscle contraction	111	44	1.57E-03
**GO:0051094**	positive regulation of developmental process	967	247	1.64E-03
**GO:0098662**	inorganic cation transmembrane transport	572	158	1.65E-03
**GO:0051963**	regulation of synapse assembly	58	28	1.69E-03
**GO:0060322**	head development	402	118	1.74E-03
**GO:0001667**	ameboidal-type cell migration	333	101	2.14E-03

Table S8 (continued).

GO ID	GO Description	Universe	COSMIC and CLINVAR	Adjusted P-value
**GO:0048638**	regulation of developmental growth	167	59	2.18E-03
**GO:0007167**	enzyme-linked receptor protein signaling pathway	795	208	2.19E-03
**GO:0008016**	regulation of heart contraction	164	58	2.57E-03
**GO:0051049**	regulation of transport	1327	324	2.69E-03
**GO:0006941**	striated muscle contraction	142	52	2.76E-03
**GO:0045595**	regulation of cell differentiation	1129	281	2.82E-03
**GO:0097435**	supramolecular fiber organization	710	188	3.17E-03
**GO:0035637**	multicellular organismal signaling	128	48	3.20E-03
**GO:0098660**	inorganic ion transmembrane transport	622	168	3.22E-03
**GO:0001505**	regulation of neurotransmitter levels	143	52	3.47E-03
**GO:0048598**	embryonic morphogenesis	308	94	3.66E-03
**GO:0010647**	positive regulation of cell communication	1374	333	3.80E-03
**GO:0006816**	calcium ion transport	313	95	4.16E-03
**GO:0009967**	positive regulation of signal transduction	1255	307	4.32E-03
**GO:0099565**	chemical synaptic transmission, postsynaptic	51	25	4.41E-03
**GO:0009719**	response to endogenous stimulus	1076	268	4.62E-03
**GO:0099587**	inorganic ion import across plasma membrane	112	43	5.31E-03
**GO:0098659**	inorganic cation import across plasma membrane	112	43	5.31E-03
**GO:0009790**	embryo development	505	140	5.37E-03
**GO:0098742**	cell-cell adhesion via plasma-membrane adhesion molecules	195	65	5.93E-03
**GO:0007169**	transmembrane receptor protein tyrosine kinase signaling pathway	511	141	6.44E-03
**GO:0023057**	negative regulation of signaling	1067	265	6.57E-03
**GO:1903522**	regulation of blood circulation	188	63	6.84E-03
**GO:0050807**	regulation of synapse organization	124	46	7.05E-03
**GO:0001508**	action potential	117	44	7.54E-03
**GO:0023056**	positive regulation of signaling	1380	332	7.60E-03
**GO:0051962**	positive regulation of nervous system development	147	52	8.38E-03
**GO:0198738**	cell-cell signaling by wnt	343	101	8.47E-03
**GO:0010631**	epithelial cell migration	261	81	8.86E-03
**GO:0043269**	regulation of ion transport	458	128	9.01E-03
**GO:0016358**	dendrite development	155	54	9.18E-03
**GO:0050803**	regulation of synapse structure or activity	129	47	9.61E-03
**GO:1903115**	regulation of actin filament-based movement	32	18	9.75E-03
**GO:1901888**	regulation of cell junction assembly	144	51	9.80E-03
**GO:0040008**	regulation of growth	416	118	9.86E-03
**GO:0007612**	learning	53	25	1.00E-02
**GO:0022607**	cellular component assembly	2547	575	1.03E-02
**GO:0010648**	negative regulation of cell communication	1060	262	1.06E-02
**GO:0042221**	response to chemical	2943	656	1.07E-02
**GO:0086001**	cardiac muscle cell action potential	73	31	1.16E-02
**GO:0071495**	cellular response to endogenous stimulus	974	243	1.17E-02
**GO:0090130**	tissue migration	267	82	1.17E-02
**GO:0090132**	epithelium migration	263	81	1.19E-02
**GO:0090596**	sensory organ morphogenesis	119	44	1.21E-02
**GO:0048738**	cardiac muscle tissue development	138	49	1.34E-02
**GO:0007269**	neurotransmitter secretion	91	36	1.45E-02
**GO:0099643**	signal release from synapse	91	36	1.45E-02
**GO:0016055**	Wnt signaling pathway	339	99	1.47E-02
**GO:0040007**	growth	515	140	1.52E-02
**GO:0048589**	developmental growth	273	83	1.55E-02
**GO:0098703**	calcium ion import across plasma membrane	36	19	1.76E-02
**GO:0099003**	vesicle-mediated transport in synapse	121	44	1.91E-02
**GO:0014706**	striated muscle tissue development	144	50	2.13E-02
**GO:0016310**	phosphorylation	1444	342	2.13E-02
**GO:0007517**	muscle organ development	179	59	2.24E-02
**GO:0034762**	regulation of transmembrane transport	385	109	2.30E-02
**GO:0006836**	neurotransmitter transport	137	48	2.39E-02
**GO:0044087**	regulation of cellular component biogenesis	774	197	2.41E-02
**GO:0086003**	cardiac muscle cell contraction	62	27	2.50E-02
**GO:0007611**	learning or memory	115	42	2.61E-02
**GO:0009968**	negative regulation of signal transduction	1009	248	2.74E-02
**GO:0090257**	regulation of muscle system process	157	53	2.86E-02
**GO:0035249**	synaptic transmission, glutamatergic	66	28	3.09E-02
**GO:0060560**	developmental growth involved in morphogenesis	135	47	3.44E-02

Table S8 (continued).

GO ID	GO Description	Universe	COSMIC and CLINVAR	Adjusted P-value
**GO:0002009**	morphogenesis of an epithelium	275	82	3.61E-02
**GO:0007264**	small GTPase mediated signal transduction	389	109	3.61E-02
**GO:0002027**	regulation of heart rate	84	33	3.80E-02
**GO:0043542**	endothelial cell migration	194	62	3.82E-02
**GO:0051668**	localization within membrane	570	150	4.30E-02
**GO:0086091**	regulation of heart rate by cardiac conduction	41	20	4.31E-02
**GO:0060828**	regulation of canonical Wnt signaling pathway	211	66	4.44E-02
**GO:0051965**	positive regulation of synapse assembly	35	18	4.46E-02
**GO:0048588**	developmental cell growth	129	45	4.73E-02
**GO:0099504**	synaptic vesicle cycle	114	41	4.75E-02
**GO:0042127**	regulation of cell population proliferation	1218	291	4.82E-02
**GO:0050890**	cognition	156	52	4.83E-02
**GO:0007158**	neuron cell-cell adhesion	16	11	4.94E-02

Table S9. Significant GO:BP enrichments for COSMIC G4 mutations leading to the loss of a G4.

GO ID	GO Description	Universe	COSMIC	Adjusted P-value
**GO:0009653**	anatomical structure morphogenesis	1871	381	1.50E-10
**GO:0016043**	cellular component organization	5370	931	4.07E-10
**GO:0032502**	developmental process	4584	804	5.37E-09
**GO:0071840**	cellular component organization or biogenesis	5539	945	1.77E-08
**GO:0048856**	anatomical structure development	4152	734	1.85E-08
**GO:0048699**	generation of neurons	969	216	1.86E-08
**GO:0007399**	nervous system development	1648	333	2.19E-08
**GO:0030182**	neuron differentiation	923	204	1.65E-07
**GO:0032501**	multicellular organismal process	5319	904	2.10E-07
**GO:0048731**	system development	2976	543	2.40E-07
**GO:0022008**	Neurogenesis	1096	233	3.56E-07
**GO:0007275**	multicellular organism development	3266	587	4.31E-07
**GO:0048666**	neuron development	729	167	5.80E-07
**GO:0030154**	cell differentiation	2860	521	8.72E-07
**GO:0048869**	cellular developmental process	2879	523	1.24E-06
**GO:0048468**	cell development	1355	273	2.92E-06
**GO:0010646**	regulation of cell communication	2727	493	9.55E-06
**GO:0023051**	regulation of signaling	2733	492	1.83E-05
**GO:0034330**	cell junction organization	518	122	2.98E-05
**GO:0031175**	neuron projection development	656	147	3.28E-05
**GO:0009966**	regulation of signal transduction	2464	447	4.09E-05
**GO:0048513**	animal organ development	2176	401	4.17E-05
**GO:0051128**	regulation of cellular component organization	1929	360	6.48E-05
**GO:0000904**	cell morphogenesis involved in differentiation	495	116	9.06E-05
**GO:0007010**	cytoskeleton organization	1309	256	0.000164105
**GO:0023052**	Signaling	5190	860	0.000200273
**GO:0007154**	cell communication	5221	863	0.000302145
**GO:0007155**	cell adhesion	1216	239	0.000305302
**GO:0050808**	synapse organization	275	72	0.000421197
**GO:0009887**	animal organ morphogenesis	582	129	0.000483951
**GO:0032989**	cellular component morphogenesis	549	123	0.000504882
**GO:0048667**	cell morphogenesis involved in neuron differentiation	381	91	0.001066523
**GO:0061564**	axon development	323	80	0.001103984
**GO:0065007**	biological regulation	10721	1650	0.001653583
**GO:0048812**	neuron projection morphogenesis	441	101	0.002100476
**GO:0120036**	plasma membrane bounded cell projection organization	1144	222	0.002358738
**GO:0048583**	regulation of response to stimulus	3327	568	0.002363352
**GO:0007409**	Axonogenesis	298	74	0.002622931
**GO:0003008**	system process	1358	257	0.002627434
**GO:0030030**	cell projection organization	1164	225	0.002676372
**GO:0035556**	intracellular signal transduction	2168	387	0.002733973
**GO:0032879**	regulation of localization	1615	298	0.003442374
**GO:0051239**	regulation of multicellular organismal process	2117	378	0.003636668
**GO:0000902**	cell morphogenesis	718	149	0.003637049
**GO:0007165**	signal transduction	4776	786	0.003677398
**GO:0098609**	cell-cell adhesion	743	153	0.004206573
**GO:0120039**	plasma membrane bounded cell projection morphogenesis	455	102	0.00520267
**GO:0006811**	ion transport	1187	227	0.005379723
**GO:0051179**	Localization	4343	718	0.007233245
**GO:0044057**	regulation of system process	392	90	0.007238929
**GO:0051716**	cellular response to stimulus	5982	962	0.007709961
**GO:0048858**	cell projection morphogenesis	459	102	0.007839606
**GO:0065008**	regulation of biological quality	2937	503	0.00859953
**GO:0097485**	neuron projection guidance	169	47	0.009226901
**GO:0007411**	axon guidance	169	47	0.009226901
**GO:0009888**	tissue development	1239	234	0.009565902
**GO:0050794**	regulation of cellular process	9523	1473	0.010601198
**GO:0006812**	cation transport	886	175	0.011607738
**GO:0032990**	cell part morphogenesis	469	103	0.012164938
**GO:0055085**	transmembrane transport	1060	203	0.017138186
**GO:0048598**	embryonic morphogenesis	308	73	0.018555392
**GO:0007417**	central nervous system development	584	122	0.022813937
**GO:0009790**	embryo development	505	108	0.02527435
**GO:0031589**	cell-substrate adhesion	290	69	0.028200129

Table S9 (continued).

GO ID	GO Description	Universe Count	COSMIC Count	Adjusted P-value
**GO:0040011**	Locomotion	1075	204	0.029753799
**GO:0050793**	regulation of developmental process	1810	322	0.033576014
**GO:0050905**	neuromuscular process	73	25	0.036424405
**GO:0048523**	negative regulation of cellular process	3896	643	0.039903379
**GO:0010975**	regulation of neuron projection development	288	68	0.042350124
**GO:0052697**	xenobiotic glucuronidation	11	8	0.042820644
**GO:0016477**	cell migration	1210	225	0.043542025
**GO:0003013**	circulatory system process	443	96	0.043990329
**GO:0048646**	anatomical structure formation involved in morphogenesis	751	149	0.045881349
**GO:0003012**	muscle system process	305	71	0.046497064
**GO:0048519**	negative regulation of biological process	4381	715	0.049876587

Table S10. Significant GO:BP enrichments for CLINVAR G4 mutations leading to the loss of a G4.

GO ID	GO Description	Universe	CLINVAR	Adjusted P-value
**GO:0006936**	muscle contraction	260	21	1.07E-06
**GO:0003012**	muscle system process	305	22	1.86E-06
**GO:0060048**	cardiac muscle contraction	111	14	5.31E-06
**GO:0006941**	striated muscle contraction	142	15	9.47E-06
**GO:0060047**	heart contraction	187	16	3.82E-05
**GO:0003015**	heart process	193	16	4.82E-05
**GO:0086003**	cardiac muscle cell contraction	62	10	6.95E-05
**GO:0086002**	cardiac muscle cell action potential involved in contraction	48	9	9.09E-05
**GO:0070252**	actin-mediated cell contraction	86	11	1.12E-04
**GO:0030048**	actin filament-based movement	113	12	1.84E-04
**GO:0086001**	cardiac muscle cell action potential	73	10	2.19E-04
**GO:0008016**	regulation of heart contraction	164	13	8.37E-04
**GO:0001508**	action potential	117	11	1.31E-03
**GO:0035637**	multicellular organismal signaling	128	11	2.48E-03
**GO:0008015**	blood circulation	363	18	2.56E-03
**GO:0003013**	circulatory system process	443	20	2.56E-03
**GO:1903522**	regulation of blood circulation	188	13	2.64E-03
**GO:0060348**	bone development	108	10	3.32E-03
**GO:0086005**	ventricular cardiac muscle cell action potential	29	6	4.45E-03
**GO:0061337**	cardiac conduction	91	9	5.54E-03
**GO:0044057**	regulation of system process	392	17	1.65E-02
**GO:0002027**	regulation of heart rate	84	8	2.02E-02
**GO:0001501**	skeletal system development	289	14	2.76E-02
**GO:0030279**	negative regulation of ossification	26	5	2.93E-02
**GO:0060537**	muscle tissue development	218	12	3.16E-02

Table S11: Significant GO:BP enrichments for COSMIC and CLINVAR G4 mutations leading to the gain of a G4.

GO ID	GO Description	Universe	COSMIC And CLINVAR	Adjusted P-value
**GO:0048856**	anatomical structure development	4152	588	5.78E-18
**GO:0032502**	developmental process	4584	634	2.01E-17
**GO:0048731**	system development	2976	448	5.64E-17
**GO:0007275**	multicellular organism development	3266	480	1.76E-16
**GO:0007399**	nervous system development	1648	279	1.92E-15
**GO:0009653**	anatomical structure morphogenesis	1871	303	2.95E-14
**GO:0048869**	cellular developmental process	2879	419	7.27E-13
**GO:0030154**	cell differentiation	2860	416	1.07E-12
**GO:0007155**	cell adhesion	1216	209	2.10E-11
**GO:0032501**	multicellular organismal process	5319	684	2.28E-11
**GO:0030029**	actin filament-based process	717	138	2.88E-10
**GO:1903508**	positive regulation of nucleic acid-templated transcription	1297	215	3.71E-10
**GO:0045893**	positive regulation of DNA-templated transcription	1297	215	3.71E-10
**GO:1902680**	positive regulation of RNA biosynthetic process	1303	215	5.95E-10
**GO:0045944**	positive regulation of transcription by RNA polymerase II	952	167	2.58E-09
**GO:0010646**	regulation of cell communication	2727	384	3.03E-09
**GO:0000902**	cell morphogenesis	718	135	3.17E-09
**GO:0023051**	regulation of signaling	2733	384	4.14E-09
**GO:0050793**	regulation of developmental process	1810	274	5.89E-09
**GO:0032989**	cellular component morphogenesis	549	110	7.78E-09
**GO:0051254**	positive regulation of RNA metabolic process	1433	227	7.96E-09
**GO:0030036**	actin cytoskeleton organization	637	121	2.50E-08
**GO:0048858**	cell projection morphogenesis	459	95	3.88E-08
**GO:0032990**	cell part morphogenesis	469	96	5.73E-08
**GO:0045935**	positive regulation of nucleobase-containing compound metabolic process	1620	245	1.21E-07
**GO:0010557**	positive regulation of macromolecule biosynthetic process	1492	229	1.43E-07
**GO:0048522**	positive regulation of cellular process	4704	594	2.02E-07
**GO:0031328**	positive regulation of cellular biosynthetic process	1568	237	2.77E-07
**GO:0048812**	neuron projection morphogenesis	441	90	2.81E-07
**GO:0007267**	cell-cell signaling	1269	200	2.89E-07
**GO:0120039**	plasma membrane bounded cell projection morphogenesis	455	92	2.89E-07
**GO:0048513**	animal organ development	2176	309	3.69E-07
**GO:0009891**	positive regulation of biosynthetic process	1593	239	4.60E-07
**GO:0009887**	animal organ morphogenesis	582	109	6.23E-07
**GO:0023052**	signaling	5190	642	8.45E-07
**GO:0048699**	generation of neurons	969	160	8.71E-07
**GO:0000904**	cell morphogenesis involved in differentiation	495	96	1.14E-06
**GO:0048468**	cell development	1355	208	1.18E-06
**GO:0007154**	cell communication	5221	644	1.30E-06
**GO:0030182**	neuron differentiation	923	153	1.64E-06
**GO:0022008**	neurogenesis	1096	175	1.72E-06
**GO:0030030**	cell projection organization	1164	183	2.34E-06
**GO:0120036**	plasma membrane bounded cell projection organization	1144	180	3.05E-06
**GO:0007010**	cytoskeleton organization	1309	199	6.53E-06
**GO:0099536**	synaptic signaling	522	96	1.86E-05
**GO:0048667**	cell morphogenesis involved in neuron differentiation	381	76	2.12E-05
**GO:0048518**	positive regulation of biological process	5294	642	2.88E-05
**GO:0031175**	neuron projection development	656	113	3.77E-05
**GO:0050804**	modulation of chemical synaptic transmission	252	56	4.32E-05
**GO:0099177**	regulation of trans-synaptic signaling	253	56	4.97E-05
**GO:0009966**	regulation of signal transduction	2464	330	5.42E-05
**GO:0022603**	regulation of anatomical structure morphogenesis	700	118	6.09E-05
**GO:0051173**	positive regulation of nitrogen compound metabolic process	2565	341	6.44E-05
**GO:0051239**	regulation of multicellular organismal process	2117	289	8.50E-05
**GO:0016043**	cellular component organization	5370	646	9.41E-05
**GO:0048666**	neuron development	729	121	9.62E-05
**GO:0031325**	positive regulation of cellular metabolic process	2522	335	9.64E-05
**GO:0099537**	trans-synaptic signaling	501	90	1.58E-04
**GO:0048589**	developmental growth	273	57	2.93E-04
**GO:0048523**	negative regulation of cellular process	3896	484	2.94E-04
**GO:0048870**	cell motility	1362	197	3.32E-04
**GO:0007268**	chemical synaptic transmission	495	88	3.49E-04
**GO:0098916**	anterograde trans-synaptic signaling	495	88	3.49E-04

Table S11 (continued).

GO ID	GO Description	Universe	COSMIC And CLINVAR	Adjusted P-value
**GO:0009987**	cellular process	14783	1556	6.39E-04
**GO:0034330**	cell junction organization	518	90	7.04E-04
**GO:0009888**	tissue development	1239	180	8.89E-04
**GO:0035556**	intracellular signal transduction	2168	288	1.05E-03
**GO:0007417**	central nervous system development	584	98	1.09E-03
**GO:0010604**	positive regulation of macromolecule metabolic process	2889	369	1.09E-03
**GO:0045595**	regulation of cell differentiation	1129	166	1.22E-03
**GO:0051128**	regulation of cellular component organization	1929	260	1.31E-03
**GO:0099587**	inorganic ion import across plasma membrane	112	30	1.40E-03
**GO:0098659**	inorganic cation import across plasma membrane	112	30	1.40E-03
**GO:0060322**	head development	402	73	1.69E-03
**GO:0071840**	cellular component organization or biogenesis	5539	653	1.79E-03
**GO:0048519**	negative regulation of biological process	4381	529	2.36E-03
**GO:0007409**	axonogenesis	298	58	2.40E-03
**GO:0009893**	positive regulation of metabolic process	3177	398	2.59E-03
**GO:0016477**	cell migration	1210	174	2.61E-03
**GO:0051716**	cellular response to stimulus	5982	697	3.27E-03
**GO:0060560**	developmental growth involved in morphogenesis	135	33	3.51E-03
**GO:0007420**	brain development	384	69	4.70E-03
**GO:0048729**	tissue morphogenesis	348	64	4.93E-03
**GO:0007165**	signal transduction	4776	568	5.32E-03
**GO:0061564**	axon development	323	60	7.13E-03
**GO:0014074**	response to purine-containing compound	75	22	7.97E-03
**GO:0048670**	regulation of collateral sprouting	9	7	8.28E-03
**GO:0048588**	developmental cell growth	129	31	9.81E-03
**GO:1990138**	neuron projection extension	112	28	1.23E-02
**GO:0050794**	regulation of cellular process	9523	1052	1.23E-02
**GO:0007167**	enzyme-linked receptor protein signaling pathway	795	120	1.26E-02
**GO:0098657**	import into cell	216	44	1.26E-02
**GO:0009719**	response to endogenous stimulus	1076	154	1.31E-02
**GO:0032970**	regulation of actin filament-based process	330	60	1.36E-02
**GO:0031344**	regulation of cell projection organization	467	78	1.63E-02
**GO:0071495**	cellular response to endogenous stimulus	974	141	1.80E-02
**GO:0120035**	regulation of plasma membrane bounded cell projection organization	453	76	1.80E-02
**GO:0098739**	import across plasma membrane	172	37	1.85E-02
**GO:0034329**	cell junction assembly	341	61	1.88E-02
**GO:0051094**	positive regulation of developmental process	967	140	1.91E-02
**GO:0048583**	regulation of response to stimulus	3327	407	1.94E-02
**GO:0007156**	homophilic cell adhesion via plasma membrane adhesion molecules	63	19	2.15E-02
**GO:0007015**	actin filament organization	395	68	2.20E-02
**GO:0032535**	regulation of cellular component size	256	49	2.21E-02
**GO:0048638**	regulation of developmental growth	167	36	2.28E-02
**GO:0007169**	transmembrane receptor protein tyrosine kinase signaling pathway	511	83	2.39E-02
**GO:0098742**	cell-cell adhesion via plasma-membrane adhesion molecules	195	40	2.62E-02
**GO:0051093**	negative regulation of developmental process	619	96	3.43E-02
**GO:0050789**	regulation of biological process	10085	1103	3.55E-02
**GO:0010243**	response to organonitrogen compound	613	95	3.86E-02
**GO:0098609**	cell-cell adhesion	743	111	4.07E-02
**GO:0048639**	positive regulation of developmental growth	83	22	4.10E-02
**GO:1905114**	cell surface receptor signaling pathway involved in cell-cell signaling	388	66	4.13E-02
**GO:0051172**	negative regulation of nitrogen compound metabolic process	1927	249	4.44E-02
**GO:0072359**	circulatory system development	729	109	4.60E-02

Table S12. Significant GO:BP enrichments for COSMIC G4 mutations leading to the gain of a G4.

GO ID	GO Description	Universe	COSMIC	Adjusted P-value
**GO:1903508**	positive regulation of nucleic acid-templated transcription	1297	121	2.51E-05
**GO:0045893**	positive regulation of DNA-templated transcription	1297	121	2.51E-05
**GO:1902680**	positive regulation of RNA biosynthetic process	1303	121	3.28E-05
**GO:0051254**	positive regulation of RNA metabolic process	1433	129	6.57E-05
**GO:0007155**	cell adhesion	1216	111	0.00034137
**GO:0045944**	positive regulation of transcription by RNA polymerase II	952	92	0.00035644
**GO:0045935**	positive regulation of nucleobase-containing compound metabolic process	1620	137	0.00095303
**GO:0046907**	intracellular transport	1373	118	0.00307086
**GO:0010557**	positive regulation of macromolecule biosynthetic process	1492	126	0.00321361
**GO:0031328**	positive regulation of cellular biosynthetic process	1568	130	0.00562818
**GO:0048856**	anatomical structure development	4152	292	0.00721462
**GO:0048522**	positive regulation of cellular process	4704	325	0.00728079
**GO:0009891**	positive regulation of biosynthetic process	1593	130	0.01262319
**GO:0010604**	positive regulation of macromolecule metabolic process	2889	213	0.01326194
**GO:0007275**	multicellular organism development	3266	236	0.01514072
**GO:0048731**	system development	2976	218	0.01552671
**GO:0051649**	establishment of localization in cell	1698	136	0.01953263
**GO:0009653**	anatomical structure morphogenesis	1871	147	0.02240799
**GO:0051173**	positive regulation of nitrogen compound metabolic process	2565	191	0.02564038
**GO:0032502**	developmental process	4584	314	0.02643181
**GO:0030036**	actin cytoskeleton organization	637	62	0.03246111
**GO:0051641**	cellular localization	2721	200	0.03443678

Table S13. Significant GO:BP enrichments for CLINVAR G4 mutations leading to the gain of a G4.

GO ID	GO Description	Universe	CLINVAR	Adjusted P-value
**GO:0001508**	action potential	117	6	4.30E-02
**GO:0086001**	cardiac muscle cell action potential	73	5	4.96E-02

Table S14. Significant GO:CC enrichments for COSMIC and CLINVAR G4 mutations.

GO ID	GO Description	Universe	COSMIC and CLINVAR	Adjusted P-value
**GO:0030054**	cell junction	1385	893	2.16E-34
**GO:0071944**	cell periphery	5199	2884	2.79E-34
**GO:0042995**	cell projection	1567	979	3.89E-30
**GO:0005886**	plasma membrane	4762	2634	7.87E-29
**GO:0120025**	plasma membrane bounded cell projection	1540	959	1.11E-28
**GO:0098590**	plasma membrane region	794	529	4.09E-24
**GO:0043005**	neuron projection	839	553	1.42E-23
**GO:0045202**	synapse	652	445	2.85E-23
**GO:0016020**	membrane	7611	3986	9.36E-21
**GO:0098797**	plasma membrane protein complex	540	364	2.72E-17
**GO:0030424**	axon	339	243	4.48E-16
**GO:0031226**	intrinsic component of plasma membrane	1632	951	2.93E-15
**GO:0005887**	integral component of plasma membrane	1560	913	2.97E-15
**GO:0015629**	actin cytoskeleton	441	300	7.75E-15
**GO:0036477**	somatodendritic compartment	444	300	3.31E-14
**GO:0005856**	cytoskeleton	1812	1033	5.15E-13
**GO:0031252**	cell leading edge	289	206	5.23E-13
**GO:0097060**	synaptic membrane	160	126	9.52E-13
**GO:0070161**	anchoring junction	707	442	2.07E-12
**GO:0043235**	receptor complex	395	266	3.53E-12
**GO:0098794**	postsynapse	293	204	3.32E-11
**GO:0005911**	cell-cell junction	369	247	8.94E-11
**GO:0097447**	dendritic tree	341	230	1.79E-10
**GO:0030425**	dendrite	338	228	2.23E-10
**GO:0005737**	cytoplasm	10633	5335	1.52E-09
**GO:0005912**	adherens junction	144	110	1.96E-09
**GO:0043228**	non-membrane-bounded organelle	4394	2308	4.06E-09
**GO:0043232**	intracellular non-membrane-bounded organelle	4393	2307	4.57E-09
**GO:0031224**	intrinsic component of membrane	2600	1406	1.40E-08
**GO:0005938**	cell cortex	209	147	2.90E-08
**GO:0099572**	postsynaptic specialization	163	119	4.37E-08
**GO:0005829**	cytosol	5137	2662	6.95E-08
**GO:0099081**	supramolecular polymer	659	396	1.20E-07
**GO:0098793**	presynapse	241	164	1.37E-07
**GO:0016021**	integral component of membrane	2485	1339	1.60E-07
**GO:0014069**	postsynaptic density	151	110	2.64E-07
**GO:0045211**	postsynaptic membrane	106	82	3.26E-07
**GO:0098984**	neuron to neuron synapse	163	117	3.38E-07
**GO:0099512**	supramolecular fiber	650	389	3.45E-07
**GO:0031982**	vesicle	3535	1861	4.51E-07
**GO:0042383**	sarcolemma	77	63	5.11E-07
**GO:0098978**	glutamatergic synapse	82	66	7.67E-07
**GO:0012505**	endomembrane system	3988	2083	8.67E-07
**GO:0032279**	asymmetric synapse	158	113	9.15E-07
**GO:0043025**	neuronal cell body	209	143	1.00E-06
**GO:0098588**	bounding membrane of organelle	1644	905	1.02E-06
**GO:0031253**	cell projection membrane	215	146	1.50E-06
**GO:0097708**	intracellular vesicle	1967	1068	1.67E-06
**GO:0031410**	cytoplasmic vesicle	1966	1067	1.92E-06
**GO:0043226**	organelle	12740	6294	1.95E-06
**GO:0019898**	extrinsic component of membrane	282	184	1.95E-06
**GO:0034702**	ion channel complex	247	164	2.08E-06
**GO:0044297**	cell body	237	158	2.57E-06
**GO:0034703**	cation channel complex	218	147	2.71E-06
**GO:0150034**	distal axon	144	103	4.39E-06
**GO:0005884**	actin filament	105	79	5.83E-06
**GO:0012506**	vesicle membrane	881	504	8.55E-06
**GO:0042734**	presynaptic membrane	53	45	1.25E-05
**GO:0030027**	lamellipodium	135	96	2.09E-05
**GO:0045177**	apical part of cell	267	172	2.18E-05
**GO:0005622**	intracellular anatomical structure	13666	6714	2.42E-05
**GO:0030659**	cytoplasmic vesicle membrane	868	494	2.79E-05
**GO:0019897**	extrinsic component of plasma membrane	156	108	3.06E-05
**GO:0099080**	supramolecular complex	978	549	6.16E-05

Table S14 (continued).

GO ID	GO Description	Universe	COSMIC and CLINVAR	Adjusted P-value
**GO:0009925**	basal plasma membrane	177	119	8.22E-05
**GO:0045178**	basal part of cell	184	123	8.39E-05
**GO:0042641**	actomyosin	64	51	8.50E-05
**GO:0031256**	leading edge membrane	111	80	1.00E-04
**GO:0098802**	plasma membrane signaling receptor complex	176	118	1.14E-04
**GO:0030863**	cortical cytoskeleton	82	62	1.36E-04
**GO:0044304**	main axon	39	34	1.57E-04
**GO:0048786**	presynaptic active zone	39	34	1.57E-04
**GO:0099634**	postsynaptic specialization membrane	45	38	1.83E-04
**GO:0016323**	basolateral plasma membrane	158	107	1.85E-04
**GO:0008328**	ionotropic glutamate receptor complex	32	29	1.85E-04
**GO:0098839**	postsynaptic density membrane	35	31	2.38E-04
**GO:0030055**	cell-substrate junction	395	238	2.60E-04
**GO:0030426**	growth cone	88	65	2.96E-04
**GO:0030427**	site of polarized growth	93	68	3.11E-04
**GO:0099513**	polymeric cytoskeletal fiber	482	284	3.57E-04
**GO:0005901**	caveola	61	48	3.61E-04
**GO:0005925**	focal adhesion	387	233	3.62E-04
**GO:0098796**	membrane protein complex	1115	614	3.62E-04
**GO:0016324**	apical plasma membrane	234	149	4.01E-04
**GO:0097517**	contractile actin filament bundle	58	46	4.01E-04
**GO:0001725**	stress fiber	58	46	4.01E-04
**GO:0000785**	chromatin	1214	663	5.89E-04
**GO:0098858**	actin-based cell projection	133	91	6.17E-04
**GO:0005604**	basement membrane	65	50	7.08E-04
**GO:0009898**	cytoplasmic side of plasma membrane	160	106	9.53E-04
**GO:0032432**	actin filament bundle	64	49	1.11E-03
**GO:0043292**	contractile fiber	173	113	1.26E-03
**GO:0005794**	Golgi apparatus	1365	737	1.26E-03
**GO:0045121**	membrane raft	209	133	1.45E-03
**GO:0005768**	endosome	810	452	1.57E-03
**GO:0030016**	myofibril	165	108	1.74E-03
**GO:0098862**	cluster of actin-based cell projections	96	68	1.83E-03
**GO:0098857**	membrane microdomain	210	133	2.05E-03
**GO:0044309**	neuron spine	90	64	2.72E-03
**GO:0044853**	plasma membrane raft	87	62	3.30E-03
**GO:0070382**	exocytic vesicle	151	99	3.62E-03
**GO:0043197**	dendritic spine	89	63	3.94E-03
**GO:0030017**	sarcomere	146	96	4.06E-03
**GO:0043229**	intracellular organelle	11898	5852	4.14E-03
**GO:0034705**	potassium channel complex	91	64	4.68E-03
**GO:0044291**	cell-cell contact zone	50	39	4.80E-03
**GO:0048471**	perinuclear region of cytoplasm	440	255	5.80E-03
**GO:0032589**	neuron projection membrane	38	31	7.16E-03
**GO:0031674**	I band	97	67	7.44E-03
**GO:0016342**	catenin complex	32	27	7.57E-03
**GO:0005769**	early endosome	295	177	7.71E-03
**GO:0030864**	cortical actin cytoskeleton	62	46	8.23E-03
**GO:0001726**	ruffle	118	79	8.51E-03
**GO:1902495**	transmembrane transporter complex	331	196	8.99E-03
**GO:0098878**	neurotransmitter receptor complex	37	30	1.18E-02
**GO:0030018**	Z disc	86	60	1.19E-02
**GO:0032420**	stereocilium	31	26	1.29E-02
**GO:0008021**	synaptic vesicle	128	84	1.42E-02
**GO:0031234**	extrinsic component of cytoplasmic side of plasma membrane	95	65	1.48E-02
**GO:0015630**	microtubule cytoskeleton	1159	622	1.58E-02
**GO:0030315**	T-tubule	36	29	1.92E-02
**GO:0016010**	dystrophin-associated glycoprotein complex	17	16	2.25E-02
**GO:0032281**	AMPA glutamate receptor complex	20	18	3.07E-02
**GO:0031941**	filamentous actin	29	24	3.62E-02
**GO:0005815**	microtubule organizing center	724	397	3.66E-02
**GO:0043194**	axon initial segment	12	12	4.29E-02
**GO:0008076**	voltage-gated potassium channel complex	82	56	4.75E-02
**GO:0034399**	nuclear periphery	96	64	5.00E-02

Table S15. Significant GO:CC enrichments for COSMIC G4 mutations.

GO ID	GO Description	Universe	COSMIC	Adjusted P-value
**GO:0071944**	cell periphery	5199	2843	5.77E-34
**GO:0030054**	cell junction	1385	879	3.22E-33
**GO:0042995**	cell projection	1567	967	4.83E-30
**GO:0005886**	plasma membrane	4762	2598	7.93E-29
**GO:0120025**	plasma membrane bounded cell projection	1540	947	1.55E-28
**GO:0045202**	synapse	652	440	3.61E-23
**GO:0098590**	plasma membrane region	794	520	5.03E-23
**GO:0043005**	neuron projection	839	545	5.77E-23
**GO:0016020**	membrane	7611	3925	2.66E-20
**GO:0030424**	axon	339	242	1.07E-16
**GO:0098797**	plasma membrane protein complex	540	358	1.46E-16
**GO:0005887**	integral component of plasma membrane	1560	900	5.34E-15
**GO:0015629**	actin cytoskeleton	441	297	6.5E-15
**GO:0031226**	intrinsic component of plasma membrane	1632	936	9.92E-15
**GO:0036477**	somatodendritic compartment	444	296	6.2E-14
**GO:0097060**	synaptic membrane	160	126	2.17E-13
**GO:0031252**	cell leading edge	289	204	4.97E-13
**GO:0005856**	cytoskeleton	1812	1018	8.96E-13
**GO:0070161**	anchoring junction	707	436	3.3E-12
**GO:0098794**	postsynapse	293	202	3.02E-11
**GO:0043235**	receptor complex	395	259	8.91E-11
**GO:0097447**	dendritic tree	341	227	2.74E-10
**GO:0030425**	dendrite	338	225	3.47E-10
**GO:0005911**	cell-cell junction	369	242	5.32E-10
**GO:0005912**	adherens junction	144	110	5.79E-10
**GO:0043228**	non-membrane-bounded organelle	4394	2279	1.51E-09
**GO:0043232**	intracellular non-membrane-bounded organelle	4393	2278	1.7E-09
**GO:0005737**	cytoplasm	10633	5250	4.16E-09
**GO:0099572**	postsynaptic specialization	163	119	1.28E-08
**GO:0005938**	cell cortex	209	146	1.83E-08
**GO:0031224**	intrinsic component of membrane	2600	1385	2.07E-08
**GO:0014069**	postsynaptic density	151	110	8.66E-08
**GO:0016021**	integral component of membrane	2485	1322	1.01E-07
**GO:0045211**	postsynaptic membrane	106	82	1.31E-07
**GO:0005829**	cytosol	5137	2619	1.67E-07
**GO:0098793**	presynapse	241	162	1.7E-07
**GO:0098984**	neuron to neuron synapse	163	116	2.92E-07
**GO:0099081**	supramolecular polymer	659	389	3.35E-07
**GO:0098978**	glutamatergic synapse	82	66	3.5E-07
**GO:0019898**	extrinsic component of membrane	282	184	4.45E-07
**GO:0034702**	ion channel complex	247	164	5.27E-07
**GO:0034703**	cation channel complex	218	147	7.57E-07
**GO:0032279**	asymmetric synapse	158	112	8.12E-07
**GO:0099512**	supramolecular fiber	650	382	9.7E-07
**GO:0042383**	sarcolemma	77	62	1.08E-06
**GO:0043025**	neuronal cell body	209	141	1.5E-06
**GO:0043226**	organelle	12740	6199	1.51E-06
**GO:0012505**	endomembrane system	3988	2050	1.58E-06
**GO:0031253**	cell projection membrane	215	144	2.15E-06
**GO:0005884**	actin filament	105	79	2.5E-06
**GO:0098588**	bounding membrane of organelle	1644	889	3E-06
**GO:0044297**	cell body	237	156	3.16E-06
**GO:0031982**	vesicle	3535	1825	3.48E-06
**GO:0097708**	intracellular vesicle	1967	1050	3.97E-06
**GO:0150034**	distal axon	144	102	4.31E-06
**GO:0031410**	cytoplasmic vesicle	1966	1049	4.54E-06
**GO:0042734**	presynaptic membrane	53	45	7.12E-06
**GO:0012506**	vesicle membrane	881	497	9.91E-06
**GO:0019897**	extrinsic component of plasma membrane	156	108	1.15E-05
**GO:0030027**	lamellipodium	135	95	2.2E-05
**GO:0005622**	intracellular anatomical structure	13666	6611	2.35E-05
**GO:0030659**	cytoplasmic vesicle membrane	868	487	3.36E-05
**GO:0042641**	actomyosin	64	51	4.76E-05
**GO:0099080**	supramolecular complex	978	542	5.51E-05
**GO:0030863**	cortical cytoskeleton	82	62	7.1E-05

Table S15 (continued).

GO ID	GO Description	Universe	COSMIC	Adjusted P-value
**GO:0048786**	presynaptic active zone	39	34	0.000103
**GO:0044304**	main axon	39	34	0.000103
**GO:0099634**	postsynaptic specialization membrane	45	38	0.000116
**GO:0031256**	leading edge membrane	111	79	0.000126
**GO:0008328**	ionotropic glutamate receptor complex	32	29	0.000128
**GO:0045177**	apical part of cell	267	167	0.000152
**GO:0030426**	growth cone	88	65	0.000155
**GO:0030427**	site of polarized growth	93	68	0.000159
**GO:0098839**	postsynaptic density membrane	35	31	0.000161
**GO:0001725**	stress fiber	58	46	0.000242
**GO:0097517**	contractile actin filament bundle	58	46	0.000242
**GO:0030055**	cell-substrate junction	395	235	0.000271
**GO:0045178**	basal part of cell	184	120	0.000291
**GO:0009925**	basal plasma membrane	177	116	0.000304
**GO:0098796**	membrane protein complex	1115	606	0.000328
**GO:0005925**	focal adhesion	387	230	0.000389
**GO:0009898**	cytoplasmic side of plasma membrane	160	106	0.000405
**GO:0005604**	basement membrane	65	50	0.000416
**GO:0098802**	plasma membrane signaling receptor complex	176	115	0.000416
**GO:0098858**	actin-based cell projection	133	90	0.000646
**GO:0099513**	polymeric cytoskeletal fiber	482	279	0.000661
**GO:0032432**	actin filament bundle	64	49	0.000663
**GO:0000785**	chromatin	1214	653	0.000752
**GO:0016323**	basolateral plasma membrane	158	104	0.000776
**GO:0005901**	caveola	61	47	0.000777
**GO:0016324**	apical plasma membrane	234	146	0.000888
**GO:0043292**	contractile fiber	173	112	0.001057
**GO:0005768**	endosome	810	447	0.001123
**GO:0030016**	myofibril	165	107	0.001521
**GO:0070382**	exocytic vesicle	151	99	0.001679
**GO:0098862**	cluster of actin-based cell projections	96	67	0.002472
**GO:0034705**	potassium channel complex	91	64	0.002626
**GO:0043229**	intracellular organelle	11898	5765	0.002825
**GO:1902495**	transmembrane transporter complex	331	196	0.002936
**GO:0045121**	membrane raft	209	130	0.003563
**GO:0030017**	sarcomere	146	95	0.003962
**GO:0005769**	early endosome	295	176	0.004405
**GO:0001726**	ruffle	118	79	0.004484
**GO:0044853**	plasma membrane raft	87	61	0.004808
**GO:0098857**	membrane microdomain	210	130	0.00492
**GO:0032589**	neuron projection membrane	38	31	0.005071
**GO:0030864**	cortical actin cytoskeleton	62	46	0.005237
**GO:0016342**	catenin complex	32	27	0.005544
**GO:0005794**	Golgi apparatus	1365	720	0.006418
**GO:0008021**	synaptic vesicle	128	84	0.007447
**GO:0098878**	neurotransmitter receptor complex	37	30	0.008507
**GO:0031234**	extrinsic component of cytoplasmic side of plasma membrane	95	65	0.008596
**GO:0044309**	neuron spine	90	62	0.009397
**GO:0031674**	I band	97	66	0.009766
**GO:0048471**	perinuclear region of cytoplasm	440	250	0.011477
**GO:0043197**	dendritic spine	89	61	0.013481
**GO:0030315**	T-tubule	36	29	0.014156
**GO:0030018**	Z disc	86	59	0.016795
**GO:0032281**	AMPA glutamate receptor complex	20	18	0.02496
**GO:0015630**	microtubule cytoskeleton	1159	611	0.027121
**GO:1990351**	transporter complex	356	204	0.02757
**GO:0031941**	filamentous actin	29	24	0.027799
**GO:0008076**	voltage-gated potassium channel complex	82	56	0.029877
**GO:0044291**	cell-cell contact zone	50	37	0.033809
**GO:0043194**	axon initial segment	12	12	0.036977
**GO:0016363**	nuclear matrix	79	54	0.037414
**GO:0099240**	intrinsic component of synaptic membrane	34	27	0.037971
**GO:0032420**	stereocilium	31	25	0.042583
**GO:0005815**	microtubule organizing center	724	391	0.043147

Table S16. Significant GO:CC enrichments for CLINVAR G4 mutations.

GO ID	GO Description	Universe	CLINVAR	Adjusted P-value
**GO:0030017**	sarcomere	146	26	9.1E-08
**GO:0031674**	I band	97	16	5.6E-04
**GO:0042383**	sarcolemma	77	14	7.7E-04
**GO:0036379**	myofilament	25	8	1.6E-03
**GO:0030315**	T-tubule	36	9	3.3E-03
**GO:0005865**	striated muscle thin filament	21	7	4.4E-03
**GO:0014704**	intercalated disc	32	8	9.8E-03
**GO:0030018**	Z disc	86	13	1.1E-02
**GO:0043202**	lysosomal lumen	87	13	1.2E-02
**GO:0033268**	node of Ranvier	12	5	2.1E-02
**GO:0043194**	axon initial segment	12	5	2.1E-02
**GO:1990584**	cardiac Troponin complex	3	3	2.2E-02
**GO:0044291**	cell-cell contact zone	50	9	4.0E-02
**GO:0005861**	troponin complex	8	4	4.8E-02

Table S17. Significant KEGG enrichments for COSMIC and CLINVAR G4 mutations.

KEGG ID	KEGG Description	Universe	COSMIC and CLINVAR	Adjusted P-value
**KEGG:04360**	Axon guidance	181	137	9.52E-13
**KEGG:04921**	Oxytocin signaling pathway	154	120	1.19E-12
**KEGG:05412**	Arrhythmogenic right ventricular cardiomyopathy	77	68	1.05E-11
**KEGG:04010**	MAPK signaling pathway	294	201	2.43E-11
**KEGG:04724**	Glutamatergic synapse	114	92	4.31E-11
**KEGG:04015**	Rap1 signaling pathway	210	151	5.83E-11
**KEGG:05200**	Pathways in cancer	529	330	1.01E-10
**KEGG:04510**	Focal adhesion	200	143	5.00E-10
**KEGG:04929**	GnRH secretion	64	57	5.44E-10
**KEGG:04728**	Dopaminergic synapse	132	100	4.36E-09
**KEGG:04261**	Adrenergic signaling in cardiomyocytes	150	111	4.46E-09
**KEGG:04072**	Phospholipase D signaling pathway	147	109	5.47E-09
**KEGG:04014**	Ras signaling pathway	234	159	1.91E-08
**KEGG:01522**	Endocrine resistance	95	75	4.97E-08
**KEGG:04725**	Cholinergic synapse	113	86	7.34E-08
**KEGG:04020**	Calcium signaling pathway	239	160	9.32E-08
**KEGG:04912**	GnRH signaling pathway	93	73	1.37E-07
**KEGG:05414**	Dilated cardiomyopathy	95	74	1.96E-07
**KEGG:04713**	Circadian entrainment	97	75	2.75E-07
**KEGG:04720**	Long-term potentiation	67	55	9.00E-07
**KEGG:05215**	Prostate cancer	97	74	9.96E-07
**KEGG:04934**	Cushing syndrome	153	107	1.96E-06
**KEGG:04750**	Inflammatory mediator regulation of TRP channels	98	74	2.12E-06
**KEGG:05410**	Hypertrophic cardiomyopathy	90	69	2.26E-06
**KEGG:04810**	Regulation of actin cytoskeleton	216	143	2.26E-06
**KEGG:04012**	ErbB signaling pathway	84	65	3.13E-06
**KEGG:04925**	Aldosterone synthesis and secretion	98	73	6.95E-06
**KEGG:04151**	PI3K-Akt signaling pathway	353	216	1.11E-05
**KEGG:04722**	Neurotrophin signaling pathway	119	85	1.35E-05
**KEGG:04911**	Insulin secretion	86	65	1.49E-05
**KEGG:04022**	cGMP-PKG signaling pathway	166	112	1.84E-05
**KEGG:05213**	Endometrial cancer	58	47	2.40E-05
**KEGG:04370**	VEGF signaling pathway	59	47	6.10E-05
**KEGG:04730**	Long-term depression	59	47	6.10E-05
**KEGG:04660**	T cell receptor signaling pathway	103	74	6.17E-05
**KEGG:05214**	Glioma	75	57	6.46E-05
**KEGG:04927**	Cortisol synthesis and secretion	64	50	7.72E-05
**KEGG:04070**	Phosphatidylinositol signaling system	97	70	9.54E-05
**KEGG:04919**	Thyroid hormone signaling pathway	121	84	1.09E-04
**KEGG:04662**	B cell receptor signaling pathway	79	59	1.11E-04
**KEGG:01521**	EGFR tyrosine kinase inhibitor resistance	79	59	1.11E-04
**KEGG:05165**	Human papillomavirus infection	331	200	1.17E-04
**KEGG:04928**	Parathyroid hormone synthesis, secretion and action	106	75	1.33E-04
**KEGG:05166**	Human T-cell leukemia virus 1 infection	219	139	1.37E-04
**KEGG:04330**	Notch signaling pathway	59	46	2.38E-04
**KEGG:05224**	Breast cancer	147	98	2.42E-04
**KEGG:05031**	Amphetamine addiction	69	52	3.10E-04
**KEGG:04727**	GABAergic synapse	89	64	3.38E-04
**KEGG:04380**	Osteoclast differentiation	125	85	3.47E-04
**KEGG:04390**	Hippo signaling pathway	157	103	3.97E-04
**KEGG:05225**	Hepatocellular carcinoma	166	108	4.11E-04
**KEGG:05205**	Proteoglycans in cancer	205	129	6.14E-04
**KEGG:05222**	Small cell lung cancer	92	65	7.28E-04
**KEGG:04935**	Growth hormone synthesis, secretion and action	120	81	8.95E-04
**KEGG:04658**	Th1 and Th2 cell differentiation	89	63	9.14E-04
**KEGG:04666**	Fc gamma R-mediated phagocytosis	96	67	1.01E-03
**KEGG:04611**	Platelet activation	124	83	1.13E-03
**KEGG:04971**	Gastric acid secretion	76	55	1.22E-03
**KEGG:05220**	Chronic myeloid leukemia	76	55	1.22E-03
**KEGG:04512**	ECM-receptor interaction	88	62	1.36E-03
**KEGG:05032**	Morphine addiction	90	63	1.60E-03
**KEGG:04310**	Wnt signaling pathway	170	108	1.92E-03
**KEGG:04926**	Relaxin signaling pathway	129	85	2.15E-03
**KEGG:04371**	Apelin signaling pathway	138	90	2.24E-03

Table S17 (continued).

KEGG ID	KEGG Description	Universe	COSMIC and CLINVAR	Adjusted P-value
**KEGG:05235**	PD-L1 expression and PD-1 checkpoint pathway in cancer	89	62	2.34E-03
**KEGG:05231**	Choline metabolism in cancer	98	67	2.83E-03
**KEGG:04910**	Insulin signaling pathway	137	89	3.06E-03
**KEGG:04922**	Glucagon signaling pathway	107	72	3.22E-03
**KEGG:04144**	Endocytosis	251	151	3.24E-03
**KEGG:05226**	Gastric cancer	148	95	3.33E-03
**KEGG:04659**	Th17 cell differentiation	105	70	6.43E-03
**KEGG:05210**	Colorectal cancer	86	59	7.15E-03
**KEGG:04540**	Gap junction	88	60	8.16E-03
**KEGG:05135**	Yersinia infection	136	87	8.37E-03
**KEGG:04520**	Adherens junction	71	50	9.16E-03
**KEGG:04930**	Type II diabetes mellitus	46	35	9.21E-03
**KEGG:00562**	Inositol phosphate metabolism	73	51	1.07E-02
**KEGG:04931**	Insulin resistance	108	71	1.10E-02
**KEGG:04916**	Melanogenesis	101	67	1.14E-02
**KEGG:04024**	cAMP signaling pathway	220	132	1.16E-02
**KEGG:04917**	Prolactin signaling pathway	70	49	1.35E-02
**KEGG:05230**	Central carbon metabolism in cancer	70	49	1.35E-02
**KEGG:04960**	Aldosterone-regulated sodium reabsorption	37	29	1.51E-02
**KEGG:05223**	Non-small cell lung cancer	72	50	1.56E-02
**KEGG:05218**	Melanoma	72	50	1.56E-02
**KEGG:05163**	Human cytomegalovirus infection	223	133	1.57E-02
**KEGG:04664**	Fc epsilon RI signaling pathway	67	47	1.68E-02
**KEGG:04625**	C-type lectin receptor signaling pathway	104	68	1.90E-02
**KEGG:04721**	Synaptic vesicle cycle	78	53	2.32E-02
**KEGG:04071**	Sphingolipid signaling pathway	119	76	2.41E-02
**KEGG:04152**	AMPK signaling pathway	121	77	2.59E-02
**KEGG:04726**	Serotonergic synapse	112	72	2.62E-02
**KEGG:05219**	Bladder cancer	41	31	2.74E-02
**KEGG:04270**	Vascular smooth muscle contraction	134	84	2.83E-02
**KEGG:04933**	AGE-RAGE signaling pathway in diabetic complications	100	65	3.26E-02
**KEGG:05221**	Acute myeloid leukemia	67	46	4.10E-02

Table S18. Significant KEGG enrichments for COSMIC G4 mutations.

GO ID	GO Description	Universe	COSMIC	Adjusted P-value
**KEGG:04611**	Platelet activation	124	81	0.002339
**KEGG:05226**	Gastric cancer	148	94	0.002757
**KEGG:04144**	Endocytosis	251	149	0.002928
**KEGG:05166**	Human T-cell leukemia virus 1 infection	219	132	0.003096
**KEGG:04926**	Relaxin signaling pathway	129	83	0.00414
**KEGG:04024**	cAMP signaling pathway	220	132	0.004174
**KEGG:04935**	Growth hormone synthesis, secretion and action	120	78	0.004212
**KEGG:04520**	Adherens junction	71	50	0.005182
**KEGG:04930**	Type II diabetes mellitus	46	35	0.005861
**KEGG:00562**	Inositol phosphate metabolism	73	51	0.006052
**KEGG:04666**	Fc gamma R-mediated phagocytosis	96	64	0.006737
**KEGG:04658**	Th1 and Th2 cell differentiation	89	60	0.007009
**KEGG:05231**	Choline metabolism in cancer	98	65	0.007447
**KEGG:04960**	Aldosterone-regulated sodium reabsorption	37	29	0.010324
**KEGG:04910**	Insulin signaling pathway	137	86	0.010713
**KEGG:04916**	Melanogenesis	101	66	0.012641
**KEGG:04721**	Synaptic vesicle cycle	78	53	0.013364
**KEGG:05135**	Yersinia infection	136	85	0.014406
**KEGG:05235**	PD-L1 expression and PD-1 checkpoint pathway in cancer	89	59	0.015883
**KEGG:04922**	Glucagon signaling pathway	107	69	0.015977
**KEGG:05230**	Central carbon metabolism in cancer	70	48	0.019532
**KEGG:05210**	Colorectal cancer	86	57	0.020522
**KEGG:05218**	Melanoma	72	49	0.022161
**KEGG:04931**	Insulin resistance	108	69	0.023655
**KEGG:04152**	AMPK signaling pathway	121	76	0.025197
**KEGG:04270**	Vascular smooth muscle contraction	134	83	0.025742
**KEGG:05163**	Human cytomegalovirus infection	223	130	0.026284
**KEGG:05220**	Chronic myeloid leukemia	76	51	0.027645
**KEGG:04659**	Th17 cell differentiation	105	67	0.030128
**KEGG:04625**	C-type lectin receptor signaling pathway	104	66	0.041098
**KEGG:05030**	Cocaine addiction	49	35	0.043077
**KEGG:04071**	Sphingolipid signaling pathway	119	74	0.045066
**KEGG:04917**	Prolactin signaling pathway	70	47	0.04558
**KEGG:04540**	Gap junction	88	57	0.047383

Table S19. Significant KEGG enrichments for CLINVAR G4 mutations.

KEGG ID	KEGG Description	Universe	CLINVAR	Adjusted P-value
**KEGG:05414**	Dilated cardiomyopathy	95	21	6.7E-07
**KEGG:05410**	Hypertrophic cardiomyopathy	90	20	1.4E-06
**KEGG:05412**	Arrhythmogenic right ventricular cardiomyopathy	77	18	3.4E-06
**KEGG:04261**	Adrenergic signaling in cardiomyocytes	150	23	1.0E-04
**KEGG:04512**	ECM-receptor interaction	88	16	5.1E-04
**KEGG:04260**	Cardiac muscle contraction	87	15	1.8E-03
**KEGG:05221**	Acute myeloid leukemia	67	12	7.9E-03
**KEGG:05230**	Central carbon metabolism in cancer	70	12	1.2E-02
**KEGG:04919**	Thyroid hormone signaling pathway	121	16	2.0E-02
**KEGG:05220**	Chronic myeloid leukemia	76	12	2.4E-02
**KEGG:04912**	GnRH signaling pathway	93	13	4.1E-02

Table S20. Significant KEGG enrichments for COSMIC and CLINVAR G4 mutations leading to a G4 loss.

KEGG ID	KEGG Description	Universe	COSMIC and CLINVAR	Adjusted P-value
**KEGG:05412**	Arrhythmogenic right ventricular cardiomyopathy	77	44	4.37E-11
**KEGG:05414**	Dilated cardiomyopathy	95	50	7.37E-11
**KEGG:05410**	Hypertrophic cardiomyopathy	90	48	1.15E-10
**KEGG:04261**	Adrenergic signaling in cardiomyocytes	150	61	1.83E-07
**KEGG:04921**	Oxytocin signaling pathway	154	60	1.62E-06
**KEGG:04010**	MAPK signaling pathway	294	95	7.23E-06
**KEGG:04015**	Rap1 signaling pathway	210	73	1.11E-05
**KEGG:04510**	Focal adhesion	200	70	1.52E-05
**KEGG:04020**	Calcium signaling pathway	239	80	1.63E-05
**KEGG:04725**	Cholinergic synapse	113	44	1.41E-04
**KEGG:04151**	PI3K-Akt signaling pathway	353	104	2.35E-04
**KEGG:04514**	Cell adhesion molecules	153	53	6.96E-04
**KEGG:05200**	Pathways in cancer	529	142	9.19E-04
**KEGG:04022**	cGMP-PKG signaling pathway	166	56	9.35E-04
**KEGG:04024**	cAMP signaling pathway	220	69	1.41E-03
**KEGG:04512**	ECM-receptor interaction	88	34	2.64E-03
**KEGG:04810**	Regulation of actin cytoskeleton	216	67	2.74E-03
**KEGG:04925**	Aldosterone synthesis and secretion	98	36	5.22E-03
**KEGG:04360**	Axon guidance	181	57	6.73E-03
**KEGG:04929**	GnRH secretion	64	26	8.78E-03
**KEGG:04713**	Circadian entrainment	97	35	9.76E-03
**KEGG:04911**	Insulin secretion	86	32	1.00E-02
**KEGG:04662**	B cell receptor signaling pathway	79	30	1.07E-02
**KEGG:04934**	Cushing syndrome	153	49	1.34E-02
**KEGG:04724**	Glutamatergic synapse	114	39	1.42E-02
**KEGG:05165**	Human papillomavirus infection	331	91	1.55E-02
**KEGG:04014**	Ras signaling pathway	234	68	1.95E-02
**KEGG:04728**	Dopaminergic synapse	132	43	2.21E-02
**KEGG:05032**	Morphine addiction	90	32	2.49E-02
**KEGG:04390**	Hippo signaling pathway	157	49	2.56E-02
**KEGG:05224**	Breast cancer	147	46	3.61E-02
**KEGG:04730**	Long-term depression	59	23	4.14E-02
**KEGG:04727**	GABAergic synapse	89	31	4.49E-02

Table S21. Significant KEGG enrichments for COSMIC G4 mutations leading to a G4 loss.

KEGG ID	KEGG Description	Universe	COSMIC	Adjusted P-value
**KEGG:05410**	Hypertrophic cardiomyopathy	90	37	9.29E-08
**KEGG:05414**	Dilated cardiomyopathy	95	38	1.37E-07
**KEGG:05412**	Arrhythmogenic right ventricular cardiomyopathy	77	32	9.49E-07
**KEGG:04261**	Adrenergic signaling in cardiomyocytes	150	43	5.39E-04
**KEGG:04020**	Calcium signaling pathway	239	59	1.65E-03
**KEGG:04921**	Oxytocin signaling pathway	154	42	2.51E-03
**KEGG:04360**	Axon guidance	181	45	1.33E-02
**KEGG:04022**	cGMP-PKG signaling pathway	166	42	1.47E-02
**KEGG:04015**	Rap1 signaling pathway	210	50	1.76E-02
**KEGG:04510**	Focal adhesion	200	48	1.92E-02
**KEGG:04024**	cAMP signaling pathway	220	51	2.94E-02
**KEGG:04151**	PI3K-Akt signaling pathway	353	74	3.78E-02

Table S22. Significant KEGG enrichments for CLINVAR G4 mutations leading to a G4 loss.

KEGG ID	KEGG Description	Universe	CLINVAR	Adjusted P-value
**KEGG:05410**	Hypertrophic cardiomyopathy	90	10	5.17E-04
**KEGG:05414**	Dilated cardiomyopathy	95	10	7.44E-04
**KEGG:05412**	Arrhythmogenic right ventricular cardiomyopathy	77	9	9.61E-04
**KEGG:04261**	Adrenergic signaling in cardiomyocytes	150	9	4.67E-02
**KEGG:05221**	Acute myeloid leukemia	67	6	5.00E-02

Table S23: Significant GO:CC enrichments for COSMIC and CLINVAR G4 mutations leading to a G4 gain.

KEGG ID	KEGG Description	Universe	COSMIC and CLINVAR	Adjusted P-value
**KEGG:04929**	GnRH secretion	64	22	1.99E-05
**KEGG:05200**	Pathways in cancer	529	89	5.62E-05
**KEGG:04724**	Glutamatergic synapse	114	30	8.27E-05
**KEGG:04725**	Cholinergic synapse	113	28	6.58E-04
**KEGG:04919**	Thyroid hormone signaling pathway	121	29	8.96E-04
**KEGG:04015**	Rap1 signaling pathway	210	42	1.20E-03
**KEGG:04261**	Adrenergic signaling in cardiomyocytes	150	33	1.45E-03
**KEGG:05202**	Transcriptional misregulation in cancer	192	39	1.70E-03
**KEGG:04713**	Circadian entrainment	97	24	3.03E-03
**KEGG:04930**	Type II diabetes mellitus	46	15	3.49E-03
**KEGG:05210**	Colorectal cancer	86	22	3.76E-03
**KEGG:04010**	MAPK signaling pathway	294	52	3.76E-03
**KEGG:04928**	Parathyroid hormone synthesis, secretion and action	106	25	4.76E-03
**KEGG:04072**	Phospholipase D signaling pathway	147	31	5.89E-03
**KEGG:04730**	Long-term depression	59	17	5.93E-03
**KEGG:04921**	Oxytocin signaling pathway	154	32	6.02E-03
**KEGG:05218**	Melanoma	72	19	7.97E-03
**KEGG:04728**	Dopaminergic synapse	132	28	1.16E-02
**KEGG:04934**	Cushing syndrome	153	31	1.22E-02
**KEGG:04512**	ECM-receptor interaction	88	21	1.52E-02
**KEGG:05213**	Endometrial cancer	58	16	1.62E-02
**KEGG:04961**	Endocrine and other factor-regulated calcium reabsorption	53	15	1.89E-02
**KEGG:04510**	Focal adhesion	200	37	1.91E-02
**KEGG:04974**	Protein digestion and absorption	103	23	2.09E-02
**KEGG:04810**	Regulation of actin cytoskeleton	216	39	2.15E-02
**KEGG:05030**	Cocaine addiction	49	14	2.72E-02
**KEGG:04720**	Long-term potentiation	67	17	2.91E-02
**KEGG:04911**	Insulin secretion	86	20	3.02E-02
**KEGG:04151**	PI3K-Akt signaling pathway	353	56	3.34E-02
**KEGG:05165**	Human papillomavirus infection	331	53	3.80E-02
**KEGG:04540**	Gap junction	88	20	4.06E-02

Table S24. Significant KEGG enrichments for COSMIC G4 mutations leading to a G4 gain.

KEGG ID	KEGG Description	Universe	COSMIC	Adjusted P-value
**KEGG:05218**	Melanoma	72	13	1.45E-02
**KEGG:04072**	Phospholipase D signaling pathway	147	20	1.59E-02
**KEGG:05030**	Cocaine addiction	49	10	2.90E-02

Table S25. Significant INTERPRO enrichments for COSMIC and CLINVAR G4 mutations.

INTERPRO ID	COSMIC and CLINVAR	UNIVERSE	FDR
**IPR011993:Pleckstrin homology-like domain**	150	446	1.41E-10
**IPR001849:Pleckstrin homology domain**	95	277	1.4E-06
**IPR011009:Protein kinase-like domain**	158	547	4.88E-06
**IPR000719:Protein kinase, catalytic domain**	146	502	9.19E-06
**IPR013098:Immunoglobulin I-set**	52	140	0.000295
**IPR008271:Serine/threonine-protein kinase, active site**	96	316	0.000295
**IPR017441:Protein kinase, ATP binding site**	113	390	0.000331
**IPR017970:Homeobox, conserved site**	65	193	0.000418
**IPR001781:Zinc finger, LIM-type**	32	75	0.001657
**IPR001452:Src homology-3 domain**	72	230	0.001677
**IPR002219:Protein kinase C-like, phorbol ester/diacylglycerol binding**	29	67	0.003026
**IPR003598:Immunoglobulin subtype 2**	76	254	0.004198
**IPR013164:Cadherin, N-terminal**	28	65	0.004198
**IPR008936:Rho GTPase activation protein**	35	95	0.013732
**IPR015425:Actin-binding FH2**	11	15	0.013732
**IPR000008:C2 calcium-dependent membrane targeting**	48	148	0.018196
**IPR020479:Homeodomain, metazoa**	34	93	0.018196
**IPR013088:Zinc finger, NHR/GATA-type**	24	57	0.021614
**IPR001025:Bromo adjacent homology (BAH) domain**	9	11	0.025203
**IPR000536:Nuclear hormone receptor, ligand-binding, core**	21	48	0.031914
**IPR009057:Homeodomain-like**	95	360	0.04254
**IPR001478:PDZ domain**	50	163	0.04254
**IPR001628:Zinc finger, nuclear hormone receptor-type**	20	46	0.045727

Table S26. Significant INTERPRO enrichments for COSMIC G4 mutations.

INTERPRO ID	COSMIC	Universe	FDR
**IPR011993:Pleckstrin homology-like domain**	147	446	1.30E-10
**IPR001849:Pleckstrin homology domain**	93	277	1.46E-06
**IPR011009:Protein kinase-like domain**	153	547	1.13E-05
**IPR000719:Protein kinase, catalytic domain**	141	502	2.55E-05
**IPR013098:Immunoglobulin I-set**	50	140	7.98E-04
**IPR017441:Protein kinase, ATP binding site**	109	390	7.98E-04
**IPR017970:Homeobox, conserved site**	63	193	7.98E-04
**IPR008271:Serine/threonine-protein kinase, active site**	91	316	1.39E-03
**IPR002219:Protein kinase C-like, phorbol ester/diacylglycerol binding**	29	67	1.98E-03
**IPR001781:Zinc finger, LIM-type**	31	75	2.42E-03
**IPR013164:Cadherin, N-terminal**	28	65	2.72E-03
**IPR001452:Src homology-3 domain**	69	230	3.73E-03
**IPR008936:Rho GTPase activation protein**	35	95	7.92E-03
**IPR015425:Actin-binding FH2**	11	15	1.09E-02
**IPR020479:Homeodomain, metazoa**	34	93	1.09E-02
**IPR003598:Immunoglobulin subtype 2**	72	254	1.39E-02
**IPR000008:C2 calcium-dependent membrane targeting**	47	148	1.68E-02
**IPR001025:Bromo adjacent homology (BAH) domain**	9	11	2.07E-02
**IPR001478:PDZ domain**	50	163	2.28E-02
**IPR013088:Zinc finger, NHR/GATA-type**	23	57	3.61E-02
**IPR009057:Homeodomain-like**	93	360	3.71E-02
**IPR015919:Cadherin-like**	39	121	4.08E-02
**IPR002126:Cadherin**	38	118	4.83E-02

Table S27. Significant INTERPRO enrichments for CLINVAR G4 mutations.

INTERPRO ID	CLINVAR	UNIVERSE	FDR
**IPR000595:Cyclic nucleotide-binding domain**	7	36	0.009358
**IPR018490:Cyclic nucleotide-binding-like**	7	39	0.009358

Table S28: Top 50 significant transcription factor enrichments for COSMIC and CLINVAR G4.

Transcription Factor ID	Transcription Factor Description	Universe	COSMIC and CLINVAR	FDR
**TF:M09636_1**	Factor: MAZ; motif: GGGMGGGGSSGGGGGGGGGGG; match class: 1	14379	7641	6.97E-262
**TF:M09973_1**	Factor: CPBP; motif: GNNRGGGHGGGGNNGGGRN; match class: 1	6788	4243	1.01E-254
**TF:M09826_1**	Factor: BTEB3; motif: CCNNSCCNSCCCCKCCCCC; match class: 1	7694	4675	1.41E-249
**TF:M07289_1**	Factor: GKLF; motif: NNNRGGNGNGGSN; match class: 1	10800	6111	2.57E-248
**TF:M07039_1**	Factor: ETF; motif: CCCCGCCCCYN; match class: 1	13890	7403	5.24E-241
**TF:M09973**	Factor: CPBP; motif: GNNRGGGHGGGGNNGGGRN	11087	6214	2.70E-238
**TF:M09984**	Factor: MAZ; motif: GGGGGAGGGGGNGRGRRRGNRG	9762	5614	4.46E-236
**TF:M12351_1**	Factor: TIEG1; motif: NCCCNSNCCCCGCCCCC; match class: 1	8412	4966	9.71E-228
**TF:M09723**	Factor: BTEB1; motif: GGGGGCGGGGCNGSGGGNGS	10228	5801	4.64E-226
**TF:M09826**	Factor: BTEB3; motif: CCNNSCCNSCCCCKCCCCC	11731	6462	1.50E-224
**TF:M09984_1**	Factor: MAZ; motif: GGGGGAGGGGGNGRGRRRGNRG; match class: 1	5696	3615	3.02E-220
**TF:M10026**	Factor: PATZ; motif: GGGGNGGGGGMKGGRRNGGNRN	8607	5037	2.42E-219
**TF:M07040_1**	Factor: GKLF; motif: NNRRGRRNGNSNNN; match class: 1	8337	4909	3.67E-219
**TF:M00986_1**	Factor: Churchill; motif: CGGGNN; match class: 1	10609	5947	4.14E-216
**TF:M09723_1**	Factor: BTEB1; motif: GGGGGCGGGGCNGSGGGNGS; match class: 1	6131	3819	2.47E-213
**TF:M12160_1**	Factor: KLF15; motif: RCCMCRCCCMCN; match class: 1	8212	4823	1.49E-208
**TF:M10432_1**	Factor: MAZ; motif: GGGMGGGGS; match class: 1	4484	2948	2.74E-203
**TF:M12351**	Factor: TIEG1; motif: NCCCNSNCCCCGCCCCC	12580	6766	2.59E-200
**TF:M10432**	Factor: MAZ; motif: GGGMGGGGS	9496	5399	2.34E-199
**TF:M00933**	Factor: Sp1; motif: CCCCGCCCCN	9913	5589	4.40E-199
**TF:M10529**	Factor: Sp1; motif: RGGGMGGRGSNGGGG	7039	4230	1.45E-197
**TF:M04953**	Factor: Sp1; motif: GGNDGGRGGCGGGG	8852	5093	1.56E-196
**TF:M02089_1**	Factor: E2F-3; motif: GGCGGGN; match class: 1	9606	5440	8.00E-196
**TF:M10112**	Factor: Miz-1; motif: NNRGGWGGGGGAGGGGMRR	8878	5103	9.30E-196
**TF:M12160**	Factor: KLF15; motif: RCCMCRCCCMCN	12959	6914	1.26E-195
**TF:M09636**	Factor: MAZ; motif: GGGMGGGGSSGGGGGGGGGGG	16533	8315	1.88E-194
**TF:M10026_1**	Factor: PATZ; motif: GGGGNGGGGGMKGGRRNGGNRN; match class: 1	5053	3219	9.11E-192
**TF:M01104_1**	Factor: MOVO-B; motif: GNGGGGG; match class: 1	5798	3599	1.13E-191
**TF:M00932_1**	Factor: Sp1; motif: NNGGGGCGGGGNN; match class: 1	6212	3805	5.40E-191
**TF:M07395_1**	Factor: Sp1; motif: NGGGGCGGGGN; match class: 1	6529	3956	1.81E-188
**TF:M00931**	Factor: Sp1; motif: GGGGCGGGGC	10524	5832	8.08E-187
**TF:M00933_1**	Factor: Sp1; motif: CCCCGCCCCN; match class: 1	5316	3340	7.30E-186
**TF:M09834**	Factor: ZNF148; motif: NNNNNNCCNNCCCCTCCCCCACCCN	7099	4227	3.40E-185
**TF:M00932**	Factor: Sp1; motif: NNGGGGCGGGGNN	10669	5892	5.01E-185
**TF:M01303**	Factor: SP1; motif: GGGGYGGGGNS	8089	4697	5.64E-183
**TF:M03876_1**	Factor: Kaiso; motif: GCMGGGRGCRGS; match class: 1	9311	5267	3.63E-182
**TF:M07436**	Factor: WT1; motif: NNGGGNGGGSGN	6637	3990	4.64E-181
**TF:M07226**	Factor: SP1; motif: NCCCCKCCCCC	8460	4865	2.87E-180
**TF:M07397**	Factor: ZBP89; motif: CCCCKCCCCCNN	7289	4306	2.95E-180
**TF:M07289**	Factor: GKLF; motif: NNNRGGNGNGGSN	14918	7675	3.00E-180
**TF:M00196**	Factor: Sp1; motif: NGGGGGCGGGGYN	10479	5792	1.13E-179

Table S28 (continued).

Transcription Factor ID	Transcription Factor Description	Universe	COSMIC and CLINVAR	FDR
**TF:M10071**	Factor: Sp1; motif: NGGGGGCGGGGCCNGGGGGGGG	8705	4978	1.41E-179
**TF:M00931_1**	Factor: Sp1; motif: GGGGCGGGGC; match class: 1	6075	3707	2.15E-179
**TF:M11529_1**	Factor: E2F-2; motif: GCGCGCGCNCS; match class: 1	14789	7621	5.14E-179
**TF:M07395**	Factor: Sp1; motif: NGGGGCGGGGN	10901	5975	1.19E-177
**TF:M00196_1**	Factor: Sp1; motif: NGGGGGCGGGGYN; match class: 1	6084	3703	4.14E-176
**TF:M09970**	Factor: KLF3; motif: NNNNNNGGGCGGGGCNNGN	7907	4589	3.12E-175
**TF:M07039**	Factor: ETF; motif: CCCCGCCCCYN	16656	8320	3.08E-174
**TF:M01104**	Factor: MOVO-B; motif: GNGGGGG	10486	5777	3.21E-173
**TF:M12703_1**	Factor: ZNF383; motif: SSNGGGMGGNGSNGGS; match class: 1	4453	2863	3.94E-173

Table S29. Count and percentage of effect of SNV calculated by thermodynamic MFE and ED changes in the G quadruplex sequence.

**Change in Stability by SNV**	**Change in multiconfirm by SNV**	**Frequency**	**Percentage**
**Further stabilized**	less diversity	6,417	17.105
**no change**	less diversity	3,835	10.222
**Destabilized**	less diversity	5,383	14.349
**Further stabilized**	no change	34	0.091
**no change**	no change	5,378	14.335
**Destabilized**	no change	39	0.104
**Further stabilized**	more diversity	3,984	10.619
**no change**	more diversity	2,849	7.594
**Destabilized**	more diversity	9,597	25.581

Table S30. Effect of transition mutation G→A in chr10:122,143,482 on potential binding for multiple transcription factors. All effects are strong.

Motif Position	Gene Symbol	Transcription factor binding match	Reference P-value	Alternate P-value	Allele Difference	Allele Effect Size
-3|6	NHLH1	tgtgtgggcAggtgggttg	0.0018	2.86E-05	2.1672	0.1850
-8|2	FOXO3	atgtgtgggcAggtgggttgg	0.0033	0.0001	2.3166	0.1431
-3|6	TAL1	tgtgtgggcAggtgggttg	0.0045	0.0001	2.3166	0.1814
-12|7	TP53	gtccattccatgtgtgggcAggtgggttgggtggggtga	0.0031	0.0001	2.3166	0.1149
-4|7	HES5	catgtgtgggcAggtgggttggg	0.0040	0.0001	2.2697	0.1547
-4|7	HES7	catgtgtgggcAggtgggttggg	0.0041	0.0002	2.2817	0.14880
1|8	USF2	gtgtgggcAggtgggtt	0.0026	0.0002	1.8992	0.1282
-11|3	EGR3	ttccatgtgtgggcGggtgggttgggtgg	3.47E-06	0.0002	-1.8709	-0.1170
-12|2	EGR3	ttccatgtgtgggcGggtgggttgggtgg	5.21E-06	0.0002	-1.8638	-0.1082
-11|2	EGR1	tccatgtgtgggcGggtgggttgggtg	6.63E-06	0.0002	-1.8447	-0.1058
-9|1	EGR2	atgtgtgggcGggtgggttgg	5.96E-06	0.0002	-1.4785	-0.1112
-11|2	EGR1	tccatgtgtgggcGggtgggttgggtg	4.59E-06	0.0003	-1.6940	-0.1215
-11|3	EGR2	ttccatgtgtgggcGggtgggttgggtgg	5.46E-06	0.0003	-1.7277	-0.1245
-12|3	EGR1	attccatgtgtgggcGggtgggttgggtggg	1.35E-05	0.0004	-1.9116	-0.1130
-8|1	EGR1	tgtgtgggcGggtgggttg	1.62E-05	0.0006	-1.9401	-0.1347
-6|3	ZNF740	tgtgtgggcGggtgggttg	4.86E-05	0.0011	-1.6642	-0.1248
-6|3	SP1	tgtgtgggcGggtgggttg	0.0001	0.0017	-1.0496	-0.1072
-6|4	KLF16	atgtgtgggcGggtgggttgg	0.0001	0.0021894	-1.5426264	-0.12738754
-7|9	SP4	cattccatgtgtgggcGggtgggttgggtgggg	0.0002	0.0023592	-1.76102043	-0.10166014
-6|4	SP1	atgtgtgggcGggtgggttgg	0.0002	0.00291348	-1.15354231	-0.11339262
-3|6	SP1	tgtgtgggcGggtgggttg	0.0002	0.00323868	-1.21127823	-0.11785306
-6|3	ZNF740	tgtgtgggcGggtgggttg	0.0002	0.00376701	-1.71699463	-0.13807642
-9|2	ZBTB7A	catgtgtgggcGggtgggttggg	0.0004	0.0038684	-1.3008656	-0.11661897
-11|5	SP4	cattccatgtgtgggcGggtgggttgggtgggg	0.0002	0.0047031	-1.86702905	-0.14590419
-6|4	SP3	atgtgtgggcGggtgggttgg	0.0003	0.00499582	-1.29081923	-0.13319603
